# Adjuvant aspirin for colorectal cancer with *PIK3CA*-mutated and COX-2 overexpressed tumours: the ASCOLT translational research study and meta-analysis

**DOI:** 10.1016/j.ebiom.2026.106389

**Published:** 2026-07-20

**Authors:** Eva Segelov, Shan Li, Isabel Li, Dmitri Mouradov, Sonia Yip, Daphne Day, Mark Jeffery, Rob Zielinski, Louise Nott, Yuntian Sun, Michael Christie, Gwo Fuang Ho, Tsu-Yi Chao, Nabilah Rahman, Estelle Foo, John Chia, Val Gebski, Han Chong Toh, Oliver M. Sieber, John Simes

**Affiliations:** a Department of Clinical Research, Faculty of Medicine and Department of Radiation Oncology, Inselspital, Bern University Hospital, University of Bern, Switzerland; b Personalised Oncology Division, Walter and Eliza Hall Institute of Medical Research, Parkville, Victoria, Australia; c NHMRC Clinical Trials Centre, University of Sydney, NSW, Australia; d Department of Medical Biology, The University of Melbourne, Parkville, Victoria, Australia; e Department of Oncology, Monash Health and Monash University, Victoria, Australia; f Oncology Department, Christchurch Hospital, New Zealand; g Medical Oncology Department, Orange Health Service, Orange, NSW, Australia; h Royal Hobart Hospital, Hobart, Tasmania, Australia; i Department of Pathology, National Cancer Center, Chinese Academy of Medical Sciences, Beijing, China; j Department of Pathology, Royal Melbourne Hospital, Melbourne, Victoria, Australia; k Clinical Oncology Department, Faculty of Medicine, Universiti Malaya, Kuala Lumpur, Malaysia; l Hematology/oncology Department, Shuang Ho Hospital, New Taipei City, Taiwan; m Biostatistics, Singapore Clinical Research Institute, Consortium for Clinical Research and Innovation Singapore, Singapore; n Division of Medical Oncology, National Cancer Centre Singapore, Singapore, Singapore; o Medical Oncology, Curie Oncology, 329563, Singapore, Singapore

**Keywords:** Colorectal cancer, Aspirin, Adjuvant treatment, Randomised, *PIK3CA* mutation, COX-2 overexpression

## Abstract

**Background:**

ASCOLT compared adjuvant aspirin versus (vs) placebo in 1587 patients with colorectal cancer (CRC) and reported no significant improvement in disease-free survival (DFS). Subsequently, two randomised controlled trials (RCTs) reported benefit of adjuvant aspirin in CRC patients with somatic PI3K-related mutations.

**Methods:**

The ASCOLT Translational Research (TR) study was a preplanned subgroup analysis by *PIK3CA* mutation status and COX-2 overexpression, and an exploratory analysis including *PTEN* mutation status. Among 778 participants who commenced study medication, tissue was evaluable for targeted next generation sequencing of *PIK3CA* and *PTEN* in 289 tumours and by Sanger sequencing for *PIK3CA* exon 9 or 20 (9/20) mutations in a further 108. PTGS2/COX-2 expression was assessed by immunohistochemistry in 450. Hazard ratio (HR) and 95% confidence intervals (CI) for DFS of aspirin vs placebo in subgroups were assessed in Cox models. A systematic review of completed RCTs of adjuvant aspirin in CRC patients with *PIK3CA* mutations was also undertaken. Registration: NCT00565708 and ACTRN12614000513617.

**Findings:**

Among 397 patients with tumour assessable for *PIK3CA*, there were 80 recurrences, 40 deaths and 86 DFS events after 5 years follow-up. Among 69 (17%) with *PIK3CA* mutations (any exon), there were 8 vs 8 DFS events on placebo vs aspirin (HR 0.93 (95% CI 0.35–2.47); in 45 (11%) with exon 9/20 mutations, 7 vs 4 events (HR 0.72 (95% CI 0.21–2.46) and in 84 (21%) with *PIK3CA* or *PTEN* mutations, 8 vs 9 events (HR 1.23 (95% CI 0.47–3.19). Tumours were positive for COX-2 overexpression in 307 (69%) with 28 vs 34 events (HR 0.99 (95% CI 0.60–1.63). A meta-analysis of trials in patients with *PIK3CA* exon 9/20 mutations showed reduced DFS events with HR 0.61 (95% CI 0.39–0.96).

**Interpretation:**

In ASCOLT TR, adjuvant aspirin was not associated with significantly improved DFS in CRC with PI3K-related mutations or COX-2 overexpression, albeit with wide confidence intervals. Combined results of the three published trials showed improved DFS among patients with *PIK3CA* exon 9/20 mutations. Moderate, but important, effects of aspirin in other patient groups have not been excluded.

**Funding:**

10.13039/501100000925National Health and Medical Research Council, Australia; Cancer Australia; National Cancer Centre Singapore Cancer Fund; 10.13039/501100016982Rising Tide Foundation; SingHealth Duke-NUS Academic Clinical Programme; 10.13039/100007419Lee Foundation; Lee Kim Tah Foundation; 10.13039/100013751Silent Foundation; 10.13039/501100025236Australasian Gastro-Intestinal Trials Group.

**Research in context**

**Evidence before this study**

Prospective randomised trials have shown that aspirin is effective in reducing recurrent adenomatous polyps in healthy individuals. Post-trial follow-up of randomised trials of aspirin in primary or secondary prevention of vascular events showed that aspirin reduced the long-term risk of colorectal cancer, as well as the risk of distant metastasis in patients who developed cancer during the trials. A possible adjuvant benefit of aspirin after definitive treatment is supported by large cohort and cancer registry studies, particularly among patients with colorectal tumours harbouring *PIK3CA* mutations or cyclooxygenase (COX)-2 overexpression. ASCOLT was the first large, randomised placebo-controlled trial of adjuvant aspirin in unselected patients with early (curable) colorectal cancer (CRC). While there was no significant improvement in disease-free survival (DFS) or overall survival with aspirin for the whole group, it prespecified *PIK3CA* mutation and COX-2 overexpression as molecular subgroups of interest for subsequent reporting in the ASCOLT translational research (TR) study.

**Added value of this study**

The ASCOLT TR study did not show any DFS benefit of adjuvant treatment in the prespecified subgroups of patients with somatic *PIK3CA* mutations or PTGS2/COX-2 overexpression, albeit with wide confidence intervals, which do not exclude a smaller effect.

**Implications of all the available evidence**

Two randomised trials (SAKK 41/13 and ALASCCA) comparing adjuvant aspirin with placebo, in patients with CRC with PI3K-related mutations recently reported reduced rates of CRC recurrences. A meta-analysis of these trials combined with ASCOLT TR shows good evidence of improved DFS among patients with somatic *PIK3CA* exon 9 or 20 mutations, but more uncertain evidence for other PI3K-related mutations, where further evidence from ongoing trials will be crucial to guide routine clinical practice. Moderate but important effects of the aspirin in other groups beyond PI3K are also not yet excluded and await evidence from ongoing trials.

## Introduction

Colorectal cancer (CRC) is the third most common cancer worldwide, with nearly 2 million diagnoses. It is the second leading cause of cancer-related deaths, accounting for almost 1 million deaths in 2022.^1^ Despite advances in therapy, outcomes for metastatic disease are often poor and there has been little progress in adjuvant therapy for many years.

Aspirin is being studied in many trials as an accessible, affordable repurposed therapy for primary and secondary cancer prevention. Purported mechanisms of action include irreversible inhibition of platelet cyclooxygenase (COX)-1 at low doses and suppression of cyclooxygenase-2 (COX-2), aka prostaglandin-endoperoxide synthase 2 (PTGS2), at higher doses, as well as multiple potential anti-inflammatory and anti-thrombotic effects.^2^, ^3^, ^4^ Observational cohort and cancer registry studies have suggested that aspirin might improve CRC survival when given in addition to standard adjuvant therapies.^5^^,^^6^

The ASCOLT trial was the first completed large randomised clinical trial (RCT) of adjuvant aspirin in CRC. It reported that 200 mg aspirin daily compared with placebo, given after standard adjuvant treatment, did not significantly improve disease-free survival (DFS).^7^ However, more moderate benefits of treatment overall or within particular subgroups (up to 25% reduction in events) were still plausible and contained within the confidence intervals (CI) of the results.

Epidemiological studies have shown potentially large benefits of aspirin in patients with tumours harbouring *PIK3CA* mutations,^8^^,^^9^ albeit with mixed results in other studies.^10^^,^^11^ Analysis of the near 1000 participants enrolled in the Nurses’ Health Study and the Health Professionals Follow-up Study, where the multivariate hazard ratio (HR) for cancer-related death was 0.18 in patients with somatic *PIK3CA* exon 9 or 20 (9/20) mutations, compared with no discernible effect in patients with *PIK3CA* wild-type tumours.^8^ Aberrations in the PI3K/AKT/mTOR pathway have been shown to play a central role in many cancers including CRC, with pathway dysregulation most commonly associated with *PIK3CA* mutations, *PIK3R1* mutations or a loss of *PTEN*.^12^

Recently, two RCTs in patients with somatic mutations of the PI3K pathway reported improvements in DFS, with a reduction in recurrences from adjuvant low dose aspirin.^13^^,^^14^ In the SAKK 41/13 trial, published in 2025, a trend to a DFS benefit was seen among patients harbouring *PIK3CA* 9/20 mutations.^13^ The more recently reported ALASCCA trial also showed improved DFS in this subgroup, and additionally in patients with other somatic alterations of the PI3K pathway (including mutations in other *PIK3CA* exons, *PIK3R1* and *PTEN*).^14^

Previous epidemiological studies reported an association of a reduced risk of new CRC with overexpression of tumour COX-2 protein among aspirin users vs non-users, where no such association was seen for cancers without overexpression.^15^ This may further extend to CRC recurrence when aspirin is given adjuvantly, given that COX-2 overexpression in CRC is generally linked to poorer outcomes,^16^ and because aspirin has a biological plausible role in reducing recurrence, particularly as PTGS2/COX-2 overexpression drives prostaglandin E2 (PGE2) production and activates the PI3K pathway.^17^^,^^18^

The ASCOLT translational substudy (ASCOLT TR) was planned to explore subgroups where larger effects of aspirin were anticipated based on the aforementioned epidemiological studies. ASCOLT TR prespecified two subgroups of special interest: somatic *PIK3CA* mutations and COX-2 overexpression by immunohistochemical assay (IHC). Additionally, we performed an exploratory analysis of other somatic alterations of the PI3K pathway, by analysing *PTEN* mutations. To put the results of ASCOLT TR in context, we report a systematic review of all completed placebo-controlled RCTs of adjuvant aspirin in patients with CRC harbouring *PIK3CA* and wider *PI3K* pathway mutations.

## Methods

### Study design and participants

Details of the ASCOLT trial have been published.^7^ In brief, ASCOLT was a double-blind, placebo-controlled RCT in 1587 patients with Dukes' C colon cancer, high-risk Dukes' B colon cancer, or Dukes’ B or C rectal cancer who had completed standard adjuvant therapy, comprising at least 3 months of chemotherapy. Patients with contraindications to aspirin, familial CRC syndromes, recent other cancers, and clinically significant history of cardiovascular disease or stroke were excluded. Patients were recruited from 11 countries and territories across Asia and the Pacific.

Eligible patients who had completed standard therapy within the past 120 days were stratified according to study centre, tumour type and stage and prior adjuvant oxaliplatin, gave written consent and were then randomised to receive aspirin 200 mg per day or placebo for 3 years, with 5 years follow-up. Recruitment occurred from February 2009 to June 2021. Patients were followed up for all events until March 31st, 2023, except in Australia and New Zealand (ANZ) where treatment and follow-up continued until June 30th, 2024.

ASCOLT TR was a preplanned sub-study among patients from ANZ plus those from sites in Singapore, Malaysia and Taiwan that were able to provide tumour tissue. The primary aim was to assess the effects of aspirin on DFS in subgroups according to the presence of *PIK3CA* mutation and PTGS2/COX-2 overexpression. Additional aims were to examine the association of these, and other molecular profiles related to the *PI3K* pathway, specifically *PTEN* status, with recurrence and death, overall and by randomised treatment.

Registration: NCT00565708 and ACTRN12614000513617.

### Tissue sampling and molecular analysis

Formalin-fixed paraffin-embedded tumour and normal tissue were retrieved from hospital archives for patients consenting to biospecimen collection. Haematoxylin and eosin (H&E)-stained tissue sections were used to identify tumour areas comprising >50% neoplastic cells. Expression of the prostaglandin-endoperoxide synthase 2 (PTGS2) or cyclooxygenase-2 (COX-2) protein, was measured by (IHC) of sections stained with antibody against PTGS2/COX-2 (clone SP21, Thermo Fisher Scientific), and classified into four categories: absent, weak, moderate and strong staining by an experienced CRC histopathologist blinded to trial data. As described by Chan et al., samples with moderate or strong staining were classified as positive for COX-2 overexpression; absent or weak staining were designated COX-2 negative.^15^ For cases with heterogeneous expression, the average intensity was recorded (Figure S1).

Genomic DNA was extracted from tumour areas using the Isolate II Genomic DNA Kit (Meridian Bioscience) and quantified using the Qubit dsDNA Broad Range DNA Assay Kit (Thermo Fisher Scientific). Next-Generation Sequencing (NGS) was undertaken using the Agilent SureSelect system. *PIK3CA* (NM_006218) and *PTEN* (NM_000314) mutations (all exons) were profiled using matched tumour and normal DNA as part of a SureSelect custom gene panel (Agilent). Library preparation, hybridisation, and target enrichment were conducted using the SureSelect XT HS2 DNA Regent Kit (Agilent), following manufacturer's instructions (Figure S2).

For tumour samples that did not pass quality control for targeted sequencing (see Appendix), mutations in *PIK3CA* 9/20 were profiled by Sanger sequencing using the BigDye Terminator v3.1 Ready Reaction Mix (Applied Biosystems) as described.^19^ Sequencing reaction products were analysed on a 3730xl DNA Analyser (Applied Biosystems), and detected mutations confirmed by resequencing of tumour and matched normal DNA from new PCR product. *PIK3CA* and *PTEN* mutations were annotated for evidence of *in vivo* or *in vitro* functional significance based on the JAX Clinical Knowledgebase (CKB CORE, https://ckb.genomenon.com/; accessed 31.03.2026) (Tables S1 and S2).^20^ Consequently, tumours were classified as having mutations by targeted NGS for *PIK3CA* or *PTEN* in 52 of 289 tumours and by Sanger sequencing for *PIK3CA* 9/20 in 17 of 108 tumours. A subset of these were further classified as known or likely oncogenic variants based on evidence for gain-of-function for *PIK3CA* and loss of function for *PTEN* (Tables S1 and S2).^21^ In post-hoc sensitivity analyses, a further classification variation of *PI3K*-related mutations was undertaken using the criteria applied in the ALASCCA trial with *PIK3CA* exon 9,20 (ALASCCA group A) limited to hot spot mutations but with other *PI3K*-related mutations (ALASCCA group B) broadened to include nonsense and splice site.^14^ *PIK3R1* mutations were not assessed in the ASCOLT TR study and so are not part of group B for this study. Exploratory analyses of additional molecular markers from NGS including HLA typing will be presented separately.

### Study outcomes and subgroups

ASCOLT TR was a preplanned subgroup analysis by *PIK3CA* mutational status and COX-2 expression, as outlined in the main protocol and main statistical analysis plan (SAP).^7^ The primary endpoint was DFS, defined as time from randomisation to documented CRC recurrence, new CRC primary or death from any cause. The primary subgroup for *PIK3CA* compared documented mutation in any exon. Additional exploratory subgroups related to *PI3K* alterations included a subgroup limited to *PIK3CA* exon 9/20 and an expanded group including other *PIK3CA* and/or *PTEN* mutations.^14^ The primary subgroup for PTGS2/COX-2 was overexpression vs no overexpression. COX-2 expression was also examined in four categories based on staining strength.

### Statistics

The detailed translational SAP was finalised on August 15th 2025 after review of emerging external trial evidence but prior to any unblinding of translational results in ASCOLT TR by randomised treatment.

Primary efficacy was assessed in the modified intention-to-treat population (mITT), defined as those who were randomly assigned and had commenced study drug. Patients who had not had an event (recurrence or death) were censored on June 30, 2024 (ANZ) or March 31, 2023 (other countries), or on the date of loss to follow-up, consent withdrawal or study completion (truncated at 5 years from randomisation).

The effects of treatment on the primary outcome of DFS and on time to recurrence were estimated using HRs with 95% CIs from Cox models. P values were obtained from log-rank tests. Sensitivity analyses were undertaken stratified by variables used for randomisation in a manner similar to that done in the main trial. Tests for interaction between treatment assignment and prespecified subgroups were performed for subgroup differences in DFS. Statistical analyses were performed using R software version 4.2.3.^22^

### Updated combined analysis of adjuvant aspirin trials in patients with somatic *PI3K* pathway mutations

A post-hoc synthesis using all relevant evidence was performed to place our results in context. This primary meta-analysis was planned after a review of emerging external evidence from other trials but prior to any unblinding of translational results from ASCOLT TR. Using standard methods for searching and meta-analysis, trials were identified comparing adjuvant aspirin vs placebo in patients with CRC with somatic *PIK3CA* mutations and other mutations associated with the PI3K pathway. The primary objective of the combined analysis was to estimate the effect of aspirin vs placebo on disease-free survival among patients with PIK3CA exon 9,20 mutations and among patients with other PI3K-related mutations. Effects of treatment on time to recurrence were also estimated as additional post-hoc sensitivity analyses. For each subgroup, a meta-analysis of HRs of DFS or time to recurrence for aspirin vs placebo was performed. Full details on the meta-analysis methods are outlined in the Supplementary Appendix.

### Ethics

Ethics approval was obtained from the Centralised Institutional Review Board of the National Cancer Centre Singapore, where the study was initiated, from the Sydney Local Ethics Committee, Australia and from ethics committees for all relevant study sites for tissue collection and translational studies.^7^ Written informed consent was obtained from each participant from whom tissue was collected. This study was conducted in accordance to the principles of ICH E6.

### Role of funders

Aspirin and placebo tablets were provided by Bayer. The Australasian Gastro-Intestinal Trials Group provided trial oversight as sponsor in Australia and New Zealand. Other funders of the study and Bayer had no role in study design, data collection analysis and interpretation or writing of the report.

## Results

**Fig. 1:**
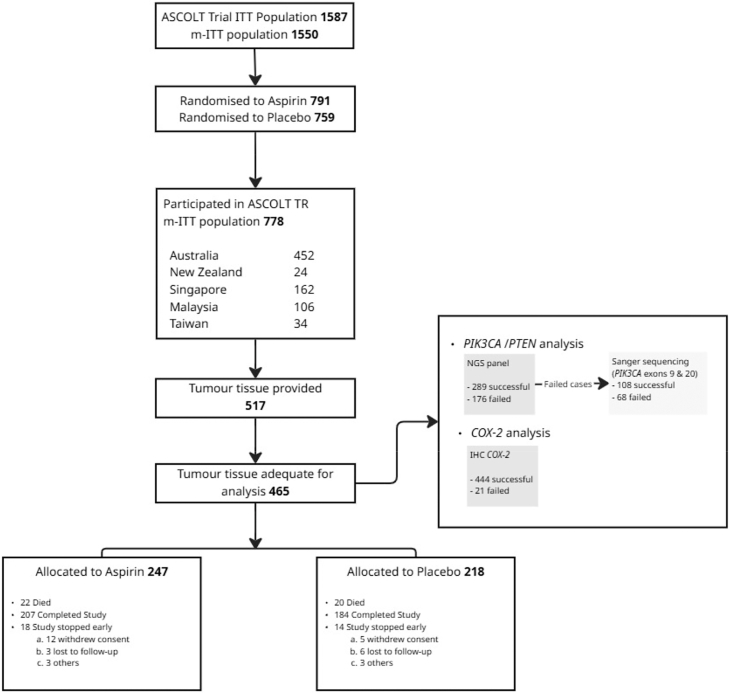
**ASCOLT TR consort diagram**. mITT, modified intention-to-treat; NGS, Next-Generation Sequencing; IHC, Immunohistochemistry.

Among 1587 people with CRC randomised on the ASCOLT trial, 778 were invited to participate and commenced study medication in the mITT population. Of 517 where tissue was accessible, 465 had sufficient tumour content upon histology review for molecular marker analysis, with 218 assigned placebo and 247 aspirin (Fig. 1).

Baseline characteristics and demographics of patients are shown in Table 1 by randomised group and were generally well balanced. Characteristics were similar to the main trial, apart for a greater predominance of Caucasian background (71% (325/465) vs 28% (432/1550)), as most participants in ASCOLT TR were enrolled in ANZ (70% (360/465) vs 30% (466/1550)).

**Table 1:** Baseline characteristics by randomised group for ASCOLT TR and ASCOLT (modified ITT populations).

	ASCOLT-TR with available tissue			ASCOLT
	Placebo N = 218	Aspirin N = 247	Total N = 465	Total N = 1550
Age				
Median (Q1–Q3)	61 (52–67)	61 (53–69)	61 (53–68)	57 (48–65)
Sex				
Female	83 (38)	117 (47)	200 (43)	653 (42)
Male	135 (62)	130 (53)	265 (57)	897 (58)
Ethnicity				
Asian	52 (24)	64 (26)	116 (25)	1035 (66)
White	156 (73)	170 (69)	326 (71)	432 (28)
Others	7 (3.3)	11 (4.5)	18 (3.9)	76 (5)
Region				
Australia & New Zealand	169 (78)	191 (77)	360 (77)	466 (30)
Singapore, Malaysia, Taiwan	49 (22)	56 (23)	105 (23)	356 (23)
ECOG				
0	185 (85)	212 (86)	397 (85)	1157 (74)
1	32 (15)	34 (14)	66 (14)	386 (25)
2	1 (0.5)	1 (0.4)	2 (0.4)	7 (1)
Time from surgery to randomisation (months)				
Median (Q1–Q3)	8.5 (7.4–9.6)	8.6 (7.5–9.9)	8.6 (7.5–9.7)	8.2 (7.1–9.5)
Disease site				
Right side of colon	72 (33)	88 (36)	160 (34)	460 (30)
Left side of colon	103 (47)	105 (43)	208 (45)	583 (37)
Rectum	42 (19)	54 (22)	96 (21)	509 (33)
T stage				
T1	6 (2.8)	9 (3.6)	15 (3.2)	41 (2.6)
T2	23 (11)	20 (8.1)	43 (9.2)	138 (9)
T3	133 (61)	164 (66)	297 (64)	976 (63)
T4	53 (24)	51 (21)	104 (22)	375 (24)
Unknown	3 (1.4)	3 (1.2)	6 (1.3)	20
N stage				
0	68 (33)	61 (26)	129 (29)	61 (26)
1	100 (48)	122 (52)	222 (50)	122 (52)
2	39 (19)	53 (22)	92 (21)	53 (22)
Unknown	11	11	22	11
Tumour type				
High risk Dukes' B colon cancer	44 (20)	38 (15)	82 (18)	271 (17)
Dukes' C colon cancer	130 (60)	150 (61)	280 (60)	770 (50)
Rectal cancer	44 (20)	59 (24)	103 (22)	509 (33)
Initial adjuvant chemotherapy				
Oxaliplatin containing	154 (71)	170 (69)	324 (70)	1183 (76)
Oxaliplatin not containing	64 (29)	77 (31)	141 (30)	366 (24)
Radiotherapy given	32 (74)	42 (70)	74 (72)	343 (67)
Chemotherapy duration				
≤6 months	158 (73)	165 (67)	323 (70)	924 (60)
>6 months	59 (27)	80 (33)	139 (30)	625 (40)

Data are n (%) or median (quartile (Q) Q1–Q3). Percent for radiotherapy given is for rectal cancer patients only. Sex data was collected as self-reported by study participants or as in the medical record.

*PIK3CA* status was able to be analysed by targeted NGS for 289 tumours, of which 52 (18%) were found to have mutations. For the subset of 176 tumours where NGS failed, exons 9/20 were able to be assessed using Sanger sequencing in 108, with mutations identified in 17 (16%). Among the total 397 patients assessable for PIK3CA by either method there were 69 patients with *PIK3CA* mutations identified, of whom 55 (80%) were classified as harbouring known or likely oncogenic variants (Table S3).

The associations of molecular marker status with baseline characteristics are shown in Table 2. *PIK3CA* mutations were more frequent in patients with right sided tumours (25% (35/140)), compared to left-sided (15% (26/175)) or rectal cancers (10% (8/81)) (p = 0.008). *PTEN* mutations were present in 20 tumours (9%) and were also more commonly seen in right sided (12% (13/112)), than left-sided (4% (5/117)) or rectal cancers (3% (2/59)) (p = 0.06). Functionally, 14 (70%) tumours were classified as harbouring known or likely oncogenic *PTEN* variants (Table S3). DNA mismatched repair/microsatellite instability (MMR/MSI) status was classified as proficient MMR/microsatellite stable (pMMR/MSS) in 87% (389/465), with deficient MMR/microsatellite instability (dMMR/MSI) in 59 (13%), being more common among those with PIK3CA mutations (25% (17/69) vs 10% (32/328)) (p = 0.001) (Table S4). PTGS2/COX-2 overexpression occurred in 69% (307/444) and was more commonly detected in ANZ patients (75% (256/340)) compared to other regions (49% 51/104)) (p < 0.001) potentially related to differences in sample fixation, storage or the use of different IHC slide providers, but there was no association with other clinicopathologic features. PI3K signalling has been shown to play a role in stabilising PTGS2/COX-2 mRNA to promote protein expression in various cancer models,^14^ however *PIK3CA* and/or *PTEN* mutation status were not associated with COX-2 overexpression in our cohort (Table S5).

**Table 2:** Baseline Characteristics by biomarker status (modified ITT population with analysable tissue).

	*PIK3CA* mutation^b^		*PIK3CA* mutation Exon 9/20		*PTEN* mutation^c^		COX-2 Overexpression	
	No^d^ N = 328	Yes N = 69	No N = 352	Yes N = 45	No N = 269	Yes N = 20	Negative N = 137	Positive N = 307
Age								
Median (Q1–Q3)	61 (53–68)	62 (53–70)	61 (53–68)	62 (56–67)	62 (54–69)	61 (49–70)	61 (52–66)	61 (53–69)
Sex								
Female	141 (43)	30 (43)	153 (43)	18 (40)	120 (45)	9 (45)	60 (44)	129 (42)
Male	187 (57)	39 (57)	199 (57)	27 (60)	149 (55)	11 (55)	77 (56)	178 (58)
Ethnicity								
Asian	79 (24)	21 (31)	86 (25)	14 (32)	26 (9.8)	4 (20)	55 (41)	60 (20)
White	234 (72)	42 (62)	249 (72)	27 (61)	228 (86)	15 (75)	78 (58)	231 (76)
Others	11 (3.4)	5 (7.4)	13 (3.7)	3 (6.8)	11 (4.2)	1 (5.0)	2 (1.5)	13 (4.3)
Unknown	4	1	4	1	4	0	2	3
Region								
Australia & New Zealand	252 (77)	51 (74)	271 (77)	32 (71)	250 (93)	17 (85)	84 (61)	256 (83)
Singapore, Malaysia, Taiwan	76 (23)	18 (26)	81 (23)	13 (29)	19 (7.1)	3 (15)	53 (39)	51 (17)
ECOG								
0	283 (86)	57 (83)	301 (86)	39 (87)	227 (84)	18 (90)	121 (88)	261 (85)
1	43 (13)	12 (17)	49 (14)	6 (13)	40 (15)	2 (10)	15 (11)	46 (15)
2	2 (0.6)	0 (0)	2 (0.6)	0 (0)	2 (0.7)	0 (0)	1 (0.7)	0 (0)
Time from surgery to randomisation (months)								
Median (Q1–Q3)	8.6 (7.5–9.9)	8.7 (7.5–9.5)	8.6 (7.5–9.9)	8.5 (7.4–9.4)	8.5 (7.4–9.8)	8.9 (7.9–10.0)	8.5 (7.5–9.7)	8.6 (7.5–9.8)
Disease site								
Right side of colon	105 (32)	35 (51)	121 (34)	19 (42)	99 (37)	13 (65)	45 (33)	103 (34)
Left side of colon	149 (46)	26 (38)	154 (44)	21 (47)	112 (42)	5 (25)	64 (47)	139 (45)
Rectum	73 (22)	8 (12)	76 (22)	5 (11)	57 (21)	2 (10)	28 (20)	64 (21)
Unknown	1	0	1	0	1	0	0	1
T stage								
T1	13 (4.0)	1 (1.4)	13 (3.7)	1 (2.2)	10 (3.7)	0 (0)	3 (2.2)	12 (3.9)
T2	29 (8.8)	7 (10)	30 (8.5)	6 (13)	24 (8.9)	2 (10)	5 (3.6)	34 (11)
T3	209 (64)	45 (65)	228 (65)	26 (58)	172 (64)	10 (50)	88 (64)	195 (64)
T4	74 (23)	14 (20)	77 (22)	11 (24)	58 (22)	8 (40)	39 (28)	63 (21)
Unknown	3 (0.9)	2 (2.9)	4 (1.1)	1 (2.2)	5 (1.9)	0 (0)	2 (1.5)	3 (1.0)
N stage								
0	87 (28)	23 (34)	97 (29)	13 (30)	74 (28)	4 (22)	41 (32)	81 (28)
1	150 (48)	41 (61)	164 (49)	27 (63)	128 (49)	13 (72)	61 (47)	154 (52)
2	77 (25)	3 (4.5)	77 (23)	3 (7.0)	58 (22)	1 (5.6)	28 (22)	59 (20)
Unknown	14	2	14	2	9	2	7	13
Tumour type								
Dukes' C colon cancer	200 (61)	40 (58)	212 (60)	28 (62)	166 (62)	12 (60)	79 (58)	187 (61)
High risk Dukes' B colon cancer	52 (16)	18 (26)	61 (17)	9 (20)	42 (16)	6 (30)	29 (21)	51 (17)
Rectal cancer (Dukes' B or Dukes' C)	76 (23)	11 (16)	79 (22)	8 (18)	61 (23)	2 (10)	29 (21)	69 (22)
Initial adjuvant chemotherapy								
Oxaliplatin containing	237 (72)	43 (62)	250 (71)	30 (67)	189 (70)	13 (65)	93 (68)	217 (71)
Oxaliplatin not containing	91 (28)	26 (38)	102 (29)	15 (33)	80 (30)	7 (35)	44 (32)	90 (29)
Radiotherapy given^a^	51 (67)	10 (91)	53 (67)	8 (100)	42 (69)	0 (0)	22 (76)	49 (71)
Chemotherapy duration								
≤6 months	223 (69)	53 (77)	243 (70)	33 (73)	189 (71)	13 (65)	92 (68)	220 (72)
>6 months	102 (31)	16 (23)	106 (30)	12 (27)	77 (29)	7 (35)	43 (32)	86 (28)
Unknown	3	0	3	0	3	0	2	1

Data are n (%) or median (Q1-Q3).

a Percent for radiotherapy given is for rectal cancer patients only.

b *PIK3CA* mutation documented in 69 (17%) of patients: for non-exon 9,20 via NGS in 289 and for exon 9,20 in 397 via NGS or Sanger. *PIK3CA* exon 9,20 mutation in 45 (11%) of 397.

c *PTEN* mutation documented in 20 (7%) of 289 via NGS.

d No documented mutation refers to no exon 9,20 mutation for all 328 and no other exon *PIK3CA* mutation for 237 or failed test for 91 via NGS.

Eighty of the 397 patients with tumours analysed for *PIK3CA* had a recurrence of CRC, with 40 deaths and 86 DFS events. DFS event rates were similar among those with and without mutations (23% vs 21%; Table 3). Likewise, rates of recurrence and DFS events (20% vs 22%) were similar among those with and without COX-2 overexpression.

**Table 3:** Clinical events by biomarker status and intervention.

Subgroup	Patients N	Recurrence n (%)	Death n	Recurrence or death n (%)
*PIK3CA* Mutation^a^				
Yes				
Placebo	34	8 (24)	3	8 (24)
Aspirin	35	6 (17)	3	8 (13)
Total	69^a^	14 (20)	6	16 (23)
No^b^				
Placebo	153	31 (20)	16	33 (22)
Aspirin	175	35 (20)	18	37 (21)
Total	328	66 (20)	34	70 (21)
*PIK3CA* Exon 9/20				
Yes				
Placebo	26	7 (27)	2	7 (27)
Aspirin	19	3 (16)	1	4 (21)
Total	45	10 (22)	3	11 (24)
No				
Placebo	161	32 (20)	17	34 (21)
Aspirin	191	38 (20)	20	41 (21)
Total	352	70 (20)	37	75 (21)
COX-2 Overexpression				
Yes				
Placebo	137	28 (20)	8	28 (20)
Aspirin	170	32 (19)	13	34 (20)
Total	307^a^	60 (20)	21	62 (20)
No				
Placebo	69	14 (20)	10	16 (23)
Aspirin	68	13 (19)	7	14 (21)
Total	137	27 (20)	17	30 (22)

a *PIK3CA* mutations documented in 69 (17%) of patients and PTGS2/COX-2 overexpression was positive in 307 (69%).

b No mutation or failed test via NGS.

Estimated effects of aspirin on DFS are shown in Table 4. Overall, among patients with available tissue for *PIK3CA* analysis, 5-year DFS was 77% (95% CI: 71%–84%) and 78% (95% CI: 72%–84%) for placebo vs aspirin respectively [HR 0.97 (95% CI 0.64–1.49)]. Among 69 patients with tumours harbouring a *PIK3CA* mutation (17%; any exon) there were 8 vs 8 events [HR 0.93 (95% CI 0.35–2.47)]. When adjusted for stratification variables this hazard ratio was 0.96 (95% CI 0.33–2.77). In 45 (11%) patients with exon 9/20 mutations, there were 7 vs 4 events [HR 0.72 (95% CI 0.21–2.46)]. For the expanded group of 84 (21%) with *PIK3CA* or *PTEN* mutations, there were 8 vs 9 events [HR 1.23 (95% CI 0.47–3.19)]. Similar results were found for additional analyses among those with known or likely oncogenic variants of *PIK3CA* and *PTEN*, and when analysed according to criteria used in the ALASCCA trial (Table S3). Sensitivity analyses of main results excluding patients with failed NGS results for *PIK3CA* mutations did not change conclusions (Table S6).

**Table 4:** Disease-free survival by biomarker status and randomised group.

Patient group	Patients N		Events n		HR (95% CI)	% DFS – 5 years (95% CI)	
	Placebo	Aspirin	Placebo	Aspirin		Placebo	Aspirin
All patients with available tissues	218	247	46	50	0.96 (0.65, 1.44)	78 (73, 84)	79 (74, 84)
Patients with *PIK3CA* Mutation Status	187	210	41	45	0.97 (0.64, 1.49)	77 (71, 84)	78 (72, 84)
*PIK3CA* Mutation^a^							
Yes	34	35	8	8	0.93 (0.35, 2.47)	76 (63, 92)	76 (63, 92)
No^b^	153	175	33	37	0.99 (0.62, 1.58)	78 (71, 85)	78 (72, 85)
*PIK3CA* Exon 9, 20 mutation							
Yes	26	19	7	4	0.72 (0.21, 2.46)	73 (58, 92)	78 (61, 100)
No	161	191	34	41	1.03 (0.65, 1.63)	78 (72, 85)	78 (72, 84)
*PTEN* mutation							
Yes	12	8	0	1	Undefined	100 (100, 100)	86 (63, 100)
No	129	140	33	32	0.86 (0.53, 1.4)	73 (66, 82)	77 (70, 84)
*PIK3CA* or *PTEN*^a^							
Yes	44	40	8	9	1.23 (0.47, 3.19)	82 (71, 94)	77 (64, 91)
No^b^	143	170	33	36	0.92 (0.57, 1.47)	76 (69, 84)	78 (72, 85)
COX-2 Overexpression							
Yes	137	170	28	34	0.99 (0.60, 1.63)	79 (72, 86)	79 (73, 86)
No	69	68	16	14	0.89 (0.43, 1.81)	77 (67, 87)	79 (69, 89)

HR, Hazard ratio; CI, Confidence Interval; DFS, Disease-free survival.

a *PIK3CA* mutation documented for non-exon 9,20 via NGS in 289 and for exon 9,20 in 397 NGS or Sanger. *PTEN* mutation documented for 289 via NGS.

b No mutation or failed test via NGS.

Estimates effects of treatment on time to recurrence were similar to DFS. For patients with *PIK3CA* mutations there were 8 vs 6 events [HR 0.70 (95% CI 0.24–2.01)]; for those with exon 9/20 mutations there were 7 v 3 events [HR 0.55 (95% CI 0.14–2.11)] (Table 3, Figure S4).

Tumours were positive for PTGS2/COX-2 overexpression in 307 (69%) with 28 vs 34 events for aspirin vs placebo [HR 0.99 (95% CI 0.60–1.63)]. When adjusted for stratification variables (including region (ANZ vs other) this hazard ratio was 1.05 (95% CI 0.63–1.74). COX-2 expression in four categories by strength of staining showed no significant differences in estimated treatment effects (Table S7).

**Fig. 2:**
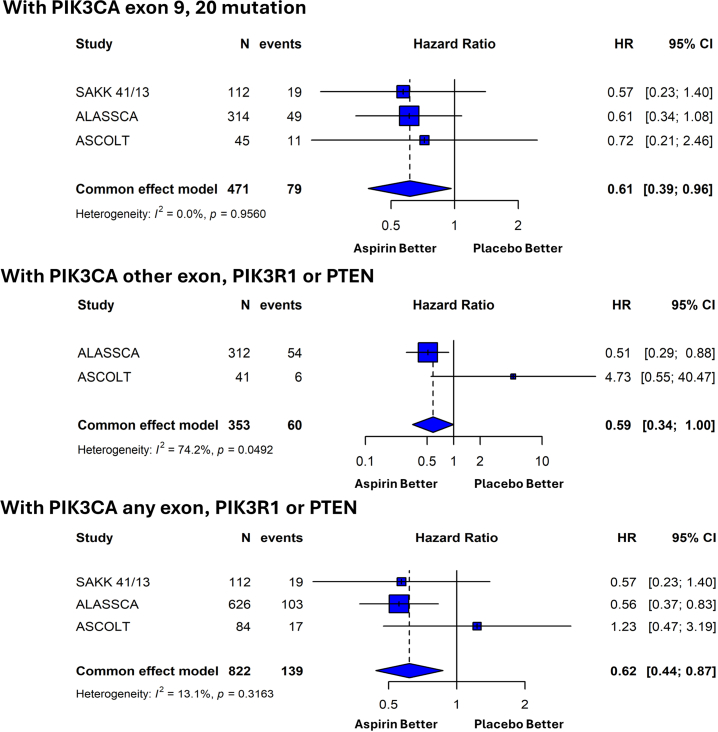
**Treatment effects of adjuvant aspirin vs placebo on disease-free survival** in patients with CRC with somatic mutations in *PI3K*-related genes. Meta-analysis of completed randomised trials: SAKK 41/13,^13^ ALASCCA,^14^ ASCOLT.^7^

A systematic review identified three randomised trials which reported data comparing adjuvant aspirin vs placebo in patients with CRC where *PIK3CA* mutations were analysed: SAKK 41/13, ALASCCA and ASCOLT-TR. Design features of the trials are shown in Table S8. A meta-analysis of estimated effects of aspirin effects on DFS according to *PI3K* mutational status is shown in Fig. 2.

For the subpopulation with *PIK3CA* exon 9/20 mutations, there were a total of 471 patients and 79 events. Combined results showed an estimated 39% reduction on DFS events, with HR 0.61 (95% CI 0.39–0.96). Among those with other *PI3K* related mutations (*PIK3CA* other exon, *PIK3R1*, *PTEN*) there were 312 patients from ALASCCA and 41 patients from ASCOLT. The combined results for this grouping showed a non-significant reduction in DFS events [HR 0.59 (95% CI 0.34–1.00)], but with evidence of heterogeneity across the two trials (interaction p = 0.049). Considering all patients with any *PI3K* mutation (*PIK3CA, PIK3R1, PTEN*), there was a significant reduction in events [HR 0.62 (95% CI 0.44–0.87)].

Similar results were obtained when ASCOLT TR results were included using the criteria described in the ALASCCA trial for classifying *PI3K*-related alterations: group A and B (Figure S3). In this case, group A included any patients with PIK3CA exon 9/20 hot spot mutations with an estimated 38% reduction in DFS events (HR 0.62 (0.39–0.97), while group B included a broader category of other PI3K related mutations from PIK3CA, PIK3R1 or PTEN with a HR of 0.59 (0.35–1.02) and evidence of heterogeneity across the two trials (interaction p = 0.03). The effects of treatment on time to recurrence in the combined analysis were also not dissimilar to the conclusions for DFS (Figures S4 and S5). These results support a clear reduction in DFS events where a somatic *PIK3CA* exon 9/20 is present, but less clear for other PI3K-related mutations.

## Discussion

Although the main ASCOLT trial did not achieve a significant reduction in DFS events, more moderate treatment effects of aspirin in the overall population, or even larger effects within important subgroups, were not excluded. Here the results from the ASCOLT TR study in prespecified subgroups of interest are reported, showing no significant effect of aspirin on DFS in patients with *PIK3CA* mutations or COX-2 overexpression. No effect was seen also in additional exploratory groups, including somatic mutations limited to exon 9 and 20 of *PIK3CA* or an expanded group including *PTEN* mutations. However, despite the overall size of the ASCOLT trial with about 30% participation in ASCOLT TR, there were a relatively small number of events. Hence the estimated treatment effect in each subgroup was associated with wide confidence intervals, which still contain possible important treatment benefits.

The epidemiological studies sparking interest in validation of aspirin benefit by RCT could not be confirmed in ASCOLT TR. By contrast, there are now two published RCTs with low dose aspirin which provide different interpretations^13^^,^^14^ The SAKK 41/13 trial, which only included patients with proven *PIK3CA* exon 9/20 mutations, randomised 112 patients but closed early due to slow recruitment. The trend to improved DFS with aspirin was not statistically significant.^13^ In the larger ALASCCA trial which reported 314 patients with *PIK3CA* exon 9/20 mutations, a statistically significant reduction in the primary endpoint of recurrence was seen, with a similar trend to improved DFS.^14^ When combining evidence from ASCOLT TR with these two RCT's, there is evidence of treatment benefit in terms of improved DFS for those patients harbouring somatic *PIK3CA* exon 9/20 mutations.

The ALASCCA trial additionally had the opportunity to more widely explore somatic alterations in relation to the PI3K pathway; this was therefore added in an exploratory analysis to ASCOLT TR. The SAKK study could not be included in this combined analysis, as eligibility was restricted to preidentified *PIK3CA* exon 9 or 20 mutations only. The combined results of ALASCCA and ASCOLT TR for patients with one of these other pathway mutations are less clear, with discordant findings, albeit based on a small number of events in ASCOLT TR. This was the case both when using the original classification of other *PIK3CA, PTEN* mutations in ASCOLT TR, and when including an expanded set of patients using the same criteria defined in the ALASCCA study. Furthermore, the same conclusions were seen in a sensitivity using a random effects model for the meta-analysis where reductions in DFS were noted only for patients with exon 9,20 mutations. A further finding in the ALASCCA trial was an apparent larger effect of aspirin treatment seen among women. While no apparent trend was seen in ASCOLT TR (Table S9), it is based on smaller numbers and this issue should still be explored further in ongoing trials.

While much of the apparent differences in results may simply be chance variation, other factors in trial design may have impacted. For example, patients commenced aspirin later in ASCOLT (at the end of adjuvant chemotherapy), as opposed to soon after surgery. The doses of aspirin also varied somewhat between studies. In addition, SAKK 41/13 did not include patients with rectal cancer, and ASCOLT commenced a decade prior to the other trials (which may have impacted staging as better imaging techniques developed and the use of adjuvant oxaliplatin was newer). There are also differences in duration of follow-up and primary trial endpoints. Finally, the study populations came from different countries/ethnicities and health systems.

The evidence in support of aspirin for a specific subset of patients would be strengthened if there were a single unifying biological rationale. While anticancer effects of aspirin are still not well understood, its primary effect is considered to occur through inhibition of the COX-2 enzyme, which is linked to tumorigenesis through activation of the PI3K pathway^14^^,^^23^ At low doses, less direct COX-2 inhibition occurs, but PTGS2/COX-2 may still be downregulated through reduction of inflammation and irreversible antiplatelet effects.^24^, ^25^, ^26^

The role of COX-2 inhibition in the adjuvant setting for *PIK3CA* mutated CRC is supported by evidence from CALGB/SWOG 80702 trial evaluating celecoxib, a direct COX-2 inhibitor.^21^ A significant reduction in DFS events in patients with somatic *PIK3CA* mutations was observed, compared with those without mutations. In this trial, the analysis was restricted to oncogenic variants more clearly related to gain-of-function and *PI3K* activation. An exploratory analysis in ASCOLT TR limited to this subset of mutations showed similar HRs to the primary ASCOLT analysis, with no change to conclusions.

Regarding PTGS2/COX-2 overexpression, the previous data from the Nurses' Health Study suggesting benefit of aspirin use in patients with CRC and COX-2 overexpression was not replicated in ASCOLT-TR. Similar to our results, no significant association was found in a retrospective analysis of aspirin use in the VICTOR study,^27^ and rapid COX-2 (and COX-1) gene turnover has been shown to limit the ability of once-daily low-dose aspirin to induce persistent COX-2 inhibition via irreversible COX-2 acetylation in nucleated cells.^28^

One limitation in comparing existing studies of PTGS2/COX-2 expression and aspirin benefit in CRC is the use of different anti-COX-2 antibodies (NHS/HPFS: clone CX229; ASCOLT-TR: clone SP21; VICTOR: polyclonal antibody). In particular, a previous study comparing specificity of SP21 and CX229 showed that SP21 preferentially recognises the S-nitrosylated form of COX-2 while CX229 detects COX-2 independent of this S-nitrosylation state, and reported that these antibodies produced substantially different staining patterns.^29^ However, a detailed follow-up study on 421 breast cancer tissues from the NHS and the Nurses’ Health Study II (NHS II) cohorts, utilising double staining techniques, subsequently demonstrated high concordance.^30^

While the additional randomised evidence from ASCOLT TR is of value in informing potential benefit of aspirin in specific molecularly defined subpopulations there are a number of limitations from these analyses. ASCOLT TR was underpowered to provide reliable estimates of treatment efficacy alone and was not designed to do so. Its relative contribution to the combined results is only modest but is appropriately weighted in the combined results. Differences between the trials described above are unlikely to have had material impact but cannot entirely be excluded. In particular, the later commencement of aspirin after the completion of other adjuvant therapies in ASCOLT TR may have contributed. Further there were different definitions for which PI3K- related mutations should be included. With respect to group A patients (based on the ALASCCA criteria) the findings for evidence of efficacy appear robust, whereas the findings for group B patients are less certain whichever criteria are applied and despite ASCOLT TR not contributing to all group B categories. Further the choice of the primary outcome of disease-free survival which included some deaths from causes unrelated to CRC may have diluted effects, but similar conclusions were also reached when examining the combined results of aspirin on recurrence rates.

There have been many other potential mechanisms by which aspirin may have anticancer effects, especially in the primary prevention setting^24^^,^^25^^,^^31^^,^^32^ though often requiring many years to emerge.^33^^,^^34^ These include immunomodulatory effects, modulation of cell metabolism modulation, effects on DNA repair, anti-inflammatory effects and antiplatelet effects potentially linked to reducing metastasis.^31^ Additional antiplatelet effects of low-dose aspirin may relate to platelet-derived lipid mediators that are inhibited by low-dose aspirin as well as prostaglandin-containing oxidised phospholipids and sphingosine-1-phosphate that may contribute to the crosstalk among platelets, cancer cells, and other cells of the tumour microenvironment.^24^ Recently Yang et al. showed that aspirin and other COX-1 inhibitors can enhance immunity to metastasis by releasing T cells from suppression by platelet-derived thromboxane A2 providing a potential mechanism for an anti-metastatic activity of aspirin.^35^^,^^36^ However, it is unclear whether each of these mechanisms would be limited to patients with *PIK3CA* mutations. In their editorial, Drew, Downie and Chan outlined a number of potential mechanisms by which aspirin may affect the PI3K/AKT pathway including inhibition of PTGS-1 (COX-1) in platelets and mechanisms independent of antiplatelet effects.^37^ Among possible mechanisms those mediated through antiplatelet effects may be of greater relevance when low doses of aspirin are used, partly due to the short half-life of aspirin limiting extra-platelet effects.

Importantly, none of the three trials discussed here have ruled out moderate but important effects of the aspirin in other groups nor ruled out that any effect of aspirin must be limited to *PI3K* related driver mutations. SAKK 41/13 and ALASCCA did not enrol such patients whilst ASCOLT was not powered to test for such interactions. Importantly the prospective meta-analysis of randomised trials of adjuvant aspirin will shed more light on this issue in time.^38^ It has identified 10 eligible trials, including these three, with the recruitment status of one not confirmed. In the nine remaining trials, with six in unselected patients and three in patients with confirmed PIK3CA mutation, there are 7043 participants in total, with many still in follow-up. This combined analysis should provide more reliable answers to clinically relevant questions about the effects of aspirin on CRC progression and its determinants.

Aspirin has considerable potential to improve CRC and potentially other cancer outcomes and this cheap, readily available drug can more easily be implemented on a global scale than many other advances in cancer therapeutics. However, a discussion regarding how much evidence is required to change practice is pertinent. Recently, the NCCN guidelines were updated to recommended that low dose aspirin be added to adjuvant therapy for the subgroup of patients with somatic *PIK3CA* mutations.^39^ While the combined analyses reported in this paper provides some support for this recommendation relating to *PIK3CA* exon 9/20 mutations, we believe it would be premature to include the broader group of other PI3K related mutations based on a single RCT. Additional evidence from other ongoing trials of adjuvant aspirin for patients with CRC and other cancers^40^, ^41^, ^42^ linked to further mechanistic studies may shed light on its benefit for a wider group of patients with molecularly defined predictive factors.

## Contributors

OMS, ES, JS, VG, HCT, JC were involved in the conceptualisation of the translational study design. SL, DM, MC and YS generated and analysed molecular data. IL, VG and JS were responsible for the biostatistical analysis. JS, OMS, and ES contributed to the writing of the original draft. All authors (ES, SL, IL, DM, SY, DD, MJ, RZ, LN, YS, MC, GFH, TYC, NR, EF, JC, VG, HCT, OMS, JS) contributed to writing, including review and editing. JS, IL, OMS, NR and ES directly accessed and verified the underlying data. All authors had access to the data in the study and have read and approved the final version of the manuscript.

## Data sharing statement

Individual participant data will not be made available. Aggregated data from ASCOLT TR are available upon reasonable request for research purposes (addressed to corresponding authors or to ascolt.study@sydney.edu.au). Study protocol and statistical analysis plan are available in the Supplementary Materials.

## Declaration of interests

JS reports research grants to his institution from the National Health and Medical Research Council (NHMRC) Australia, Bayer, BMS, Roche, Amgen, and MSD, and advisory board fees to his institution from FivepHusion. OS declares research grants from NHMRC and AGITG. DD declares a research grant from Gilead and a travel grant from Novartis. RZ reports honoraria from BMS, Janssen, Merck, The Limbic, Astra Zeneca and Pierre Fabre. LN declares consulting fees to her company from BMS, Pfizer, MSD, Amgen, honoraria from Novartis and Eli Lilly and travel support from Astra-Zeneca. GFH reports grants to his institution and/or honoraria/support from Eli Lily, Regeneron Pharmaceuticals, Merck Sharp & Dohme, AB Science, Astellas, Tessa Therapeutics, Roche, Arcus Bioscience, AstraZeneca, Pfizer, Janssen Research, Mirati Therapeutics, Novartis, Amgen, Boehringer Ingelheim, mAbxience Research, Taiho, Easai, Gene Solutions, IPSEN, Dr Reddy's, Servier and Zuellig Pharma. All other authors declare no competing interests.

## Appendix A Supplementary data

Appendix

ASCOLT Protocol

ASCOLT_TR_SAP

## References

[1] Bray F., Laversanne M., Sung H. (2024). Global cancer statistics 2022: GLOBOCAN estimates of incidence and mortality worldwide for 36 cancers in 185 countries. CA Cancer J Clin.

[2] Kanwal R., Jawed B., Zakir S.K. (2026). The anti-metastatic role of aspirin in cancer: a systematic review. Int J Mol Sci.

[3] Patrono C. (2015). The multifaceted clinical readouts of platelet inhibition by low-dose aspirin. J Am Coll Cardiol.

[4] Galli M., Cortellini G., Occhipinti G., Rossini R., Romano A., Angiolillo D.J. (2024). Aspirin hypersensitivity in patients with atherosclerotic cardiovascular disease. J Am Coll Cardiol.

[5] Qiao Y., Yang T., Gan Y. (2018). Associations between aspirin use and the risk of cancers: a meta-analysis of observational studies. BMC Cancer.

[6] Bosetti C., Santucci C., Gallus S., Martinetti M., La Vecchia C. (2020). Aspirin and the risk of colorectal and other digestive tract cancers: an updated meta-analysis through 2019. Ann Oncol.

[7] Chia J.W.K., Segelov E., Deng Y. (2025). Aspirin after completion of standard adjuvant therapy for colorectal cancer (ASCOLT): an international, multicentre, phase 3, randomised, double-blind, placebo-controlled trial. Lancet Gastroenterol Hepatol.

[8] Liao X., Lochhead P., Nishihara R. (2012). Aspirin use, tumor PIK3CA mutation, and colorectal-cancer survival. N Engl J Med.

[9] Domingo E., Church D.N., Sieber O. (2013). Evaluation of PIK3CA mutation as a predictor of benefit from nonsteroidal anti-inflammatory drug therapy in colorectal cancer. J Clin Oncol.

[10] Kothari N., Kim R., Jorissen R.N. (2015). Impact of regular aspirin use on overall and cancer-specific survival in patients with colorectal cancer harboring a PIK3CA mutation. Acta Oncol.

[11] Gray R., Cantwell M., Coleman H. (2017). Aspirin use and colon cancer survival in a population-based cohort study. Clin Transl Gastroenterol.

[12] Hall D.C.N., Benndorf R.A. (2022). Aspirin sensitivity of PIK3CA-mutated Colorectal Cancer: potential mechanisms revisited. Cell Mol Life Sci.

[13] Güller U., Hayoz S., Horber D. (2025). Adjuvant aspirin treatment in PIK3CA mutated Colon cancer patients: the SAKK 41/13 - prospective randomized placebo-controlled double-blind trial. Clin Cancer Res.

[14] Martling A., Hed Myrberg I., Nilbert M. (2025). Low-Dose aspirin for PI3K-Altered localized colorectal cancer. N Engl J Med.

[15] Chan A.T., Ogino S., Fuchs C.S. (2007). Aspirin and the risk of colorectal cancer in relation to the expression of COX-2. N Engl J Med.

[16] Ogino S., Kirkner G.J., Nosho K. (2008). Cyclooxygenase-2 expression is an independent predictor of poor prognosis in colon cancer. Clin Cancer Res.

[17] Yang J., Wang X., Gao Y. (2020). Inhibition of PI3K-AKT signaling blocks PGE2-Induced COX-2 expression in lung adenocarcinoma. Onco Targets Ther.

[18] Sheng J., Sun H., Yu F.B., Li B., Zhang Y., Zhu Y.T. (2020). The role of Cyclooxygenase-2 in colorectal cancer. Int J Med Sci.

[19] Day F.L., Jorissen R.N., Lipton L. (2013). PIK3CA and PTEN gene and exon mutation-specific clinicopathologic and molecular associations in colorectal cancer. Clin Cancer Res.

[20] Patterson S.E., Statz C.M., Yin T., Mockus S.M. (2019). Utility of the JAX Clinical Knowledgebase in capture and assessment of complex genomic cancer data. NPJ Precis Oncol.

[21] Nowak J.A., Twombly T., Ma C. (2024). Improved Survival with Adjuvant Cyclooxygenase 2 Inhibition in PIK3CA-Activated Stage III Colon Cancer: CALGB/SWOG 80702 (Alliance). J Clin Oncol.

[22] R Core Team (2023). https://www.R-project.org/.

[23] Goldberg R.M., Meyerhardt J.A. (2025). An aspirin a day for improved colorectal cancer outcomes. N Engl J Med.

[24] Patrignani P., Patrono C. (2016). Aspirin and Cancer. J Am Coll Cardiol.

[25] Sankaranarayanan R., Kumar D.R., Altinoz M.A., Bhat G.J. (2020). Mechanisms of colorectal cancer prevention by Aspirin-A literature review and perspective on the role of COX-Dependent and -Independent pathways. Int J Mol Sci.

[26] Patrono C., Burn J., Patrignani P., Langley R.E. (2026). Platelet activation, aspirin, and cancer: from basic science to clinical trials. Pharmacol Rev.

[27] Yanagisawa Y., Johnstone E., Davidson B., Kerr D.J., Tomlinson I.P., Midgley R. (2013). Evaluation of PIK3CA mutation as a predictor of benefit from nonsteroidal anti-inflammatory drug therapy in colorectal cancer. J Clin Oncol.

[28] Sostres C., Gargallo C.J., Lanas A. (2014). Aspirin, cyclooxygenase inhibition and colorectal cancer. World J Gastrointest Pharmacol Ther.

[29] Jindal S., Pennock N.D., Klug A. (2020). S-nitrosylated and non-nitrosylated COX2 have differential expression and distinct subcellular localization in normal and breast cancer tissue. NPJ Breast Cancer.

[30] Yaghjyan L., Eliassen A.H., Colditz G. (2022). Associations of aspirin and other anti-inflammatory medications with breast cancer risk by the status of COX-2 expression. Breast Cancer Res.

[31] Sun M., Yu J., Wan J., Dou X., Chen X., Ye F. (2025). Role of aspirin in cancer prevention. Cancer Treat Res Commun.

[32] Burn J., Sheth H. (2016). The role of aspirin in preventing colorectal cancer. Br Med Bull.

[33] Rothwell P.M., Fowkes F.G., Belch J.F., Ogawa H., Warlow C.P., Meade T.W. (2011). Effect of daily aspirin on long-term risk of death due to cancer: analysis of individual patient data from randomised trials. Lancet.

[34] Rothwell P., Wilson M., Price J., Belch J.F.F., Meade T.W., Mehta Z. (2012). Effect of daily aspirin on risk of cancer metastasis: a study of incident cancers during randomised controlled trials. Lancet.

[35] Yang J., Yamashita-Kanemaru Y., Morris B.I. (2025). Aspirin prevents metastasis by limiting platelet TXA_2_ suppression of T cell immunity. Nature.

[36] Langley R.E., Burn J. (2025). Understanding how aspirin prevents metastasis. N Engl J Med.

[37] Drew D.A., Downie J.M., Chan A.T. (2025). “PIK”ing the right patients for adjuvant aspirin therapy for colorectal cancer. Clin Cancer Res.

[38] Burdett S., Fisher D., Tierney J., Meade A., Nankivell M., Langley R. (2024). https://www.crd.york.ac.uk/PROSPERO/view/CRD42023453156.

[39] National Comprehensive Cancer Network (NCCN) (2025). http://www.nccn.org.

[40] Langley R.E., Wilson R.H., Cafferty F.H. (2019). Aspirin as adjuvant treatment for cancer: feasibility results from the Add-Aspirin randomised trial. Lancet Gastroenterol Hepatol.

[41] Michel P., Boige V., André T. (2018). Aspirin versus placebo in stage III or high-risk stage II colon cancer with PIK3CA mutation: a French randomised double-blind phase III trial (PRODIGE 50-ASPIK). Dig Liver Dis.

[42] Miyamoto K., Takashima A., Mizusawa J. (2019). Efficacy of aspirin for stage III colorectal cancer: a randomized double-blind placebo-controlled trial (JCOG1503C, EPISODE-III trial). Jpn J Clin Oncol.

