# Supplementary material for: Adjuvant aspirin for colorectal cancer with PIK3CA-mutated and COX-2 overexpressed tumours: the ASCOLT translational research study and meta-analysis
Source: eBioMedicine. 2026 Jul 20;130:106389. doi: 10.1016/j.ebiom.2026.106389 (PMC13393707; doi:10.1016/j.ebiom.2026.106389)
Supplement: Appendix [file mmc1.docx]

**Aspirin, *PIK3CA*, COX-2 and colorectal cancer. Supplementary Appendix**

**Table of Contents**

[Investigators, Sites and Acknowledgments 2](#_Toc231397305)

[Supplementary Laboratory Methods 4](#_Toc231397306)

[Supplementary Meta-analysis Methods 7](#_Toc231397307)

[Supplementary Tables. 9](#_Toc231397308)

[Table S1. Variants identified in *PIK3CA* (NM_006218) annotated . 9](#_Toc231397309)

[Table S2. Variants identified in PTEN (NM_000314) annotated 11](#_Toc231397310)

[Table S3. Disease-free survival by biomarker status for oncogenic variants of *PIK3CA* and *PTEN* mutations and for *PIK3CA* hot spot mutations. 12](#_Toc231397311)

[Table S4. Association of MMR/MSI status and *PIK3CA* mutations 13](#_Toc231397312)

[Table S5. Association of Cox-2 Overexpression and PI3K-related mutations.^1^ 13](#_Toc231397314)

[Table S6. Disease-free survival by PIK3CA mutation status and randomised treatment: sensitivity analysis. 14](#_Toc231397316)

[Table S7 Disease-free survival by COX-2 Expression (ordered categories). 14](#_Toc231397317)

[Table S8 Completed randomised trials of adjuvant aspirin in patients with CRC harbouring PI3K-related mutations 15](#_Toc231397318)

[Table S9 Disease-free survival by sex and randomised group. 16](#_Toc231397319)

[Supplementary Figures 17](#_Toc231397320)

[Figure S1. COX-2 immunohistochemistry illustrations. 17](#_Toc231397321)

[Figure S2. Target region coverage metrics for tumour and normals samples. 18](#_Toc231397322)

[Figure S3. Treatment effects on disease-free survival in patients with somatic alterations of PI3K pathway as per the ALASCCA criteria 19](#_Toc231397323)

[Figure S4. Treatment effects time to recurrence in patients with somatic alterations of PI3K pathway. 20](#_Toc231397324)

[Figure S5. Treatment effects on time to recurrence in patients with somatic alterations of PI3K pathway: as per the ALASCCA criteria 21](#_Toc231397325)

[Supplementary References 22](#_Toc231397326)

# Investigators, Sites and Acknowledgments

**Site Investigators by Region (number of patients)**

**Australia** (406): Robert Zielinski, Orange Healthcare Service; Louise Nott, Royal Hobart Hospital; Fiona Day, Calvary Mater Newcastle Hospital; Tom van Hagen, St John of God Subiaco; Lucy Corke , Newcastle Private Hospital; Theresa Hayes, Southwest Oncology; Ray Asghari, Bankstown-Lidcombe Hospital; Shamsudheen Padinharakam, Launceston General Hospital; Stephen Begbie, Port Macquarie Base Hospital; Sunil Rai, Tamworth Hospital; Karen Briscoe, Coffs Harbour Health Campus; Matthew Wong, Gosford Hospital; Andrew Strickland, Monash Health; Madhu Singh, Barwon Health; Guy van Hazel, Kevin Jasas, Sir Charles Gairdner Hospital; Ayesha Saqib, Goulburn Valley Health; Aflah Roohullah, Campbelltown Hospital; Narayan Karanth, Michail Charakidis, Royal Darwin Hospital; Megan Barnet, St Vincent’s Hospital Sydney; Ratnesh Srivastav, The Tweed Hospital; Geoffrey Chong, Ballarat Health Service; Craig Underhill, Border Medical Oncology; Melissa Eastgate, Royal Brisbane and Women’s Hospital; Suresh Varma, Nathan Bain, Townsville Hospital; Timothy Price, Lyell McEwin Hospital; Lisa Horvath, Chris O’Brien Lifehouse; Niall Tebbutt, Austin Health; Bhaskar Karki, Toowoomba Hospital; Nick Pavlakis, Connie Diakos, Genesis Care North Shore.

**Malaysia** (24)

Gwo Fuang Ho, University Malaya Medical Centre;

**New Zealand** (16)

Mark Jeffery, Christchurch Hospital; Christopher Jackson, Dunedin Hospital

**Singapore** (64)

John Chia, Chee Kian Tham, Han Chong Toh, National Cancer Centre.

**Taiwan** (19)

Tsu-Yi Chao, TMU Shuang Ho Hospital.

**Other Acknowledgements**

**International Trial Coordinating Centre**

National Cancer Centre Singapore: Hang Chong Toh, Estelle Foo, John Chia

**Singapore Clinical Research Institute (SCRI) Team**

(Biostatistics team, Research Informatics team and Data Management team for Statistical support, randomization and electronic data capture system for ASCOLT main).

Nabilah Rahman (Study Statistician)

**NHMRC Clinical Trials Centre**

**Translational Research Coordination**: Sonia Yip, Garry Chang, Michelle Cummins, Shayla Sharmin, Ashley Lee, MoonSun Jung. **Biostatistics**: Isabel Li, Val Gebski**. Clinical Fellows:** Katrin Sjoquist, Derrick Siu, Jane So, Joanna Lee, Deborah Zhou. **Trial operations**: Izabella Pokorski; Carla Jensen, Kate Wilson, Nicole Wong, Cheryl Friend, Christine Aiken, Sandra Bahamad, Jaclyn Verghis, Preeti Dave, Annie Yeung, Danielle Parker, Catherine O’Connor, Evonne Tim, Estrella Gonzalez-Alloy, Fathalla Ali and Martijn Oostendorp. **Reviewers:** Ava Tan, Peta Skeers

**Australasian Gastro-Intestinal Trials Group:** Russel Conley, Joanne Cory, Nisha Berthon-Jones, L Carolan.

**Sieber Laboratory Team, WEHI**

Oliver Sieber, Shan Li, Dmitri Mouradov, Claire Storey, Yuntian Sun, Michael Christie

# Supplementary Laboratory Methods

**Assessment of PTGS2/COX-2 protein expression.** Tissue sections were stained with antibody against COX-2 (clone SP21, Thermo Fisher Scientific, TA503316, 1:50), on a Dako Omnis platform (Agilent). Antigen retrieval was performed using High pH EnVision FLEX Target Retrieval Solution (Agilent, K800421-2) at 97 °C for 40 min. Sections were incubated with primary antibody for 1 h and secondary antibody (EnVision+/HRP, Rabbit, Agilent, K400311-2) for 30 min followed by EnVision FLEX DAB+ Substrate Chromogen (Agilent, GV82511-2) treatment for 10 min and counterstaining with hematoxylin.

Tumour PTGS2/COX-2 expression was evaluated by pathologist (YS) and classified into four categories of absent, weak, moderate and strong staining. Normal colonic epithelial cells served as positive and immune cells as negative internal controls. As described by Chan et al,^1,2^ samples with absent or weak staining were considered COX-2 negative and samples with moderate or strong staining were considered COX-2 positive for overexpression (Figure S1). For cases with heterogeneous expression, the average intensity was recorded.

A random sample of 98 cases was re-examined for COX-2 expression by a second observer (MC), unaware of other data, and the concordance between the two observers was 0.84 (κ = 0.67; P < 0.0001), indicating substantial agreement and consistent with previous studies reported by Ogino *et al.*, who had two independent observers rescore their COX-2 slides with kappa values of 0.62 and 0.75.^3^

***PIK3CA* and *PTEN* mutation detection.** Formalin-fixed paraffin-embedded tumour and normal tissues were retrieved from hospital archives. Hematoxylin and eosin (H&E)-stained tissue sections were used to identify tumour areas comprising >50% neoplastic cells and macrodissected from serial sections. Genomic DNA was extracted using the Isolate II Genomic DNA Kit (Meridian Bioscience) and quantified using the Qubit dsDNA Broad Range DNA Assay Kit (Thermo Fisher Scientific).

*PIK3CA* (NM_006218) and *PTEN* (NM_000314) mutations (all exons) were profiled using matched tumour and normal DNA as part of a SureSelect custom gene panel (Agilent). Library preparation, hybridization, and target enrichment were conducted using the SureSelect XT HS2 DNA Regent Kit (Agilent) following manufacturer’s instructions. Amplified libraries were quality-checked using an Agilent TapeStation System with High Sensitivity D1000 ScreenTape Assay Kits and quantified using the Quant-iT PicoGreen dsDNA Assay Kit (Thermo Fisher Scientific). Libraries were pooled and sequenced on an Illumina NextSeq 1000 platform using a 300-cycle (2 × 150 bp) paired-end run at the Ramaciotti Centre for Genomics, UNSW Sydney.

For tumour samples that did not pass quality control for targeted sequencing, mutations in *PIK3CA* exon 9 and exon 20 were profiled by Sanger sequencing using the BigDye Terminator v3.1 Ready Reaction Mix (Applied Biosystems) as described previously.^3^ Sequencing reaction products were analysed on a 3730xl DNA Analyzer (Applied Biosystems), and detected mutations confirmed by resequencing of tumour and matched normal DNA from new PCR product.

Out of 289 tumour samples sequenced by NGS, 195 were sequenced as paired tumour-germline and 94 as tumour only. *PIK3CA* and *PTEN* mutations were annotated for evidence of *in vivo* or *in vitro* functional significance based on the JAX Clinical Knowledgebase (CKB CORE, <https://ckb.genomenon.com/>; accessed 31.03.2026) (Tables S1 and S2). For *PIK3CA* mutations not present in the database, mutation functional classification was regarded as “unknown”. For *PTEN* mutations not present in the database, protein truncating mutations were regarded as “loss of function - predicted (LOFP)”, the remainder regarded as “unknown”. For tumours with multiple *PIK3CA* or *PTEN* mutations, the strongest function classification across all variants in the tumour was used for the tumour-level classification.

**Targeted sequencing and UMI-based preprocessing.** Targeted sequencing of tumour and normal samples included incorporation of unique molecular identifiers (UMIs) to enable consensus deduplication and error correction. Raw FASTQ files were trimmed using the AGeNT toolkit (v3.0.6), specifically the trimmer-3.0.5.jar module, which removed adapter sequences and extracted UMI tags from paired end reads.

**Alignment and consensus BAM generation.** Trimmed reads were aligned to the human reference genome (GRCh38/hg38) using BWA-MEM (v0.7.17). SAM files were converted to BAM format using Samtools (v1.14), and consensus deduplication was then performed using AGeNT’s creak-1.0.5.jar in hybrid mode. The resulting BAM files were sorted, indexed, and stored using Picard tools (v2.25.0) for use in downstream applications.

**Quality control and coverage metrics.** Sequencing quality and target enrichment were assessed using Picard’s CollectTargetedPcrMetrics tool, with a predefined BED file corresponding to the targeted regions. Additional depth and coverage metrics were calculated using GATK’s DepthOfCoverage tool (v4.2.2.0). Only samples in which at least 80% of targeted probes achieved a minimum of 20-fold coverage were retained for further analysis (Figure S2).

**Somatic variant calling.** Somatic variant calling was performed using Mutect2 (GATK v4.2.2.0) and VarScan2 (v2.4.4). For samples with a matched normal, paired tumour-normal mode was used to improve specificity by filtering germline variants and technical artefacts. Tumour samples without a matched normal were analysed in single-sample mode using both Mutect2 and VarScan2. Additionally, all normal samples were processed independently to support the creation of a panel of normals (PoN), which was used to filter recurrent artefacts and common germline variants in the tumour-only analyses.

Mutect2 output was filtered using the FilterMutectCalls tool, and variant calls from both Mutect2 and VarScan2 were annotated using SnpEff (v5.0). Following annotation, variants were retained if they had an alternate allele frequency of at least 10% and a minimum depth of 10 reads. Only variants predicted to alter protein coding were included, based on the following functional consequence terms: NON_SYNONYMOUS_CODING, STOP_GAINED, FRAME_SHIFT, START_GAINED, SPLICE_SITE_DONOR, CODON_INSERTION, CODON_DELETION, SPLICE_SITE_ACCEPTOR, CODON_CHANGE_PLUS_CODON_INSERTION, and CODON_CHANGE_PLUS_CODON_DELETION.

.

# Supplementary Meta-analysis Methods

An updated post-hoc synthesis using all relevant evidence to place our results in context was performed. Relevant trials were identified from the PROSPERO planned prospective meta-analysis (PMA) of randomised controlled trials of adjuvant aspirin in colorectal cancer.^1^ In addition, we used standard, extensive Cochrane search methods on the following databases up to 14 August 2025: Medline OvidSP, Embase OvidSP and World Health Organization (WHO) International Clinical Trials Registry Platform (ICTRP) for all registered and ongoing trials. We identified and considered for inclusion any trial with the key words: ‘aspirin’, ‘acetylsalicylic acid’, ‘adjuvant’, ‘colorectal neoplasm’, ‘colon cancer’, ‘randomized controlled trial’. Potentially eligible trials were then manually screened (by two reviewers) for those randomised placebo-controlled trials of adjuvant aspirin in CRC that included patients with *PIK3CA* mutations and had been completed.

Among 121 references, no additional trials to the PROSPERO planned PMA^5^ were identified and the only completed trials including patients with PIK3CA mutations were SAKK41/13,^6^ ALASCCA^7^ and ASCOLT.^8^

Characteristics of each of these trials are shown in Table S6. All trials compared adjuvant aspirin with placebo given together with any adjuvant chemotherapy in SAKK 44/13 and ALASCCA but following any adjuvant chemotherapy in ASCOLT TR. All trials were judged to have a low risk of bias based on Cochrane risk-of-bias 2 assessment

The primary objective was to assess the effect of aspirin compared with placebo on disease-free survival (DFS) events as defined according to each trial. An additional objective was to assess the effect of aspirin on time to recurrence. Combined analyses were then undertaken on DFS among patients in two subgroups of PI3K- related mutations using the criteria defined in the ALASCCA trial: group A with *PIK3CA* exon 9, 20 mutations and group B with other PI3K-related mutations (*PIK3CA* non-exon 9, 20, *PIK3R1* or *PTEN*).

For each subgroup, a meta-analysis was conducted of hazard ratios (HRs) of DFS for aspirin versus placebo by first transforming reported HRs and their 95% confidence intervals to the log scale. Standard errors for each study’s log-HR were derived from the confidence limits as $\mathrm{SE}ᵢ = (log(upperᵢ) - log(lowerᵢ)) / 3.92$, where 3.92 corresponds to twice the 97.5th percentile of the standard normal distribution (2 × 1.96), reflecting the width of a two-sided 95% confidence interval in standard error units. A fixed-effect model was then used to combine study-specific estimates, with each study weighted by the inverse of its squared standard error ($wᵢ = 1/SEᵢ^{2}$), such that more precisely estimated studies contributed proportionally more to the pooled estimate. ⁹ The pooled log-HR was computed as the precision-weighted mean $\thetâ = \Sigma wᵢ\thetâᵢ / \Sigma wᵢ$. Between-study heterogeneity was evaluated using Cochran’s Q statistic, calculated as the weighted sum of squared deviations of study-specific log-HRs from the pooled estimate ($Q = \Sigma wᵢ(\thetâᵢ - \thetâ)^{2}$), and tested against a χ² distribution with k−1 degrees of freedom, where k is the number of studies; notably, Q was computed using fixed-effect weights $wᵢ = 1/SEᵢ^{2}$ in accordance with standard practice. ^12^ The between-study variance τ² was estimated using the DerSimonian–Laird moment-based estimator ($\taû^{2} = max(0, (Q-(k-1))/c$), where $c = \Sigma wᵢ - \Sigma wᵢ^{2}/\Sigma wᵢ$, with confidence intervals for τ² and τ obtained via the Jackson method, which derives confidence limits by inverting the Q statistic distribution. ^10, 11^ The relative magnitude of heterogeneity was further quantified using the I² statistic ($I^{2} = max(0, (Q-(k-1))/Q) \times100\%$), which represents the proportion of total variance attributable to between-study heterogeneity rather than sampling error; values of 0% indicate no observed heterogeneity, with larger values indicating increasing heterogeneity.¹² The pooled log-HR and 95% confidence interval were subsequently back-transformed to the HR scale by exponentiation for interpretation. A random-effects model was also applied in a sensitivity analysis, in which study-specific weights incorporated both within-study sampling variance and the DerSimonian–Laird estimated between-study variance ($wᵢ = 1/(SEᵢ^{2} + \taû^{2})$), such that the contribution of each study reflects not only its precision but also the extent of heterogeneity across studies. Compared to the fixed-effect model, this approach redistributes weight away from large, precise studies toward smaller studies, yielding a more conservative pooled estimate and wider confidence intervals when heterogeneity is present. A supplementary analysis of effects of treatment on time to recurrence was also undertaken using the inverse variance method.

# Supplementary Tables.

## **Table S1.** **Variants identified in *PIK3CA* (NM_006218)** annotated with exon location, number of times each variant was identified, and functional classification based on the JAX Clinical Knowledgebase (CKB CORE, <https://ckb.genomenon.com/>; accessed 31.03.2026).^4^ For *PIK3CA* mutations not present in the database, mutation functional classification was regarded as “unknown”. GOF = Gain of function, GOFP = Gain of function - predicted.

| **Mutation** | **Protein change** | **Transcript change** | **Coding exon** | **Intron** | **Count** | **Variant in database** | **Functional classification** |
| --- | --- | --- | --- | --- | --- | --- | --- |
| NON_SYNONYMOUS_CODING | H1047R | c.3140A>G | 20 | - | 19 | yes | GOF |
| NON_SYNONYMOUS_CODING | E545K | c.1633G>A | 9 | - | 11 | yes | GOF |
| NON_SYNONYMOUS_CODING | E542K | c.1624G>A | 9 | - | 4 | yes | GOF |
| NON_SYNONYMOUS_CODING | C420R | c.1258T>C | 7 | - | 2 | yes | GOF |
| NON_SYNONYMOUS_CODING | E545A | c.1634A>C | 9 | - | 2 | yes | GOF |
| NON_SYNONYMOUS_CODING | E545G | c.1634A>G | 9 | - | 2 | yes | GOF |
| NON_SYNONYMOUS_CODING | R88Q | c.263G>A | 1 | - | 2 | yes | GOF |
| NON_SYNONYMOUS_CODING | R108H | c.323G>A | 1 | - | 2 | yes | GOF |
| NON_SYNONYMOUS_CODING | K111E | c.331A>G | 1 | - | 2 | yes | GOF |
| NON_SYNONYMOUS_CODING | N345K | c.1035T>A | 4 | - | 1 | yes | GOF |
| NON_SYNONYMOUS_CODING | R357Q | c.1070G>A | 5 | - | 1 | yes | Unknown |
| NON_SYNONYMOUS_CODING | G359R | c.1075G>C | 5 | - | 1 | no | Unknown |
| NON_SYNONYMOUS_CODING | R38C | c.112C>T | 1 | - | 1 | yes | GOF |
| FRAME_SHIFT | E39Pfs*15 | c.113_114insACCATCATCAGGTGAACTGTGGGGCATCCACT | 1 | - | 1 | no | Unknown |
| NON_SYNONYMOUS_CODING | C378R | c.1132T>C | 5 | - | 1 | yes | GOF |
| NON_SYNONYMOUS_CODING | C378Y | c.1133G>A | 5 | - | 1 | yes | Unknown |
| CODON_CHANGE_PLUS_CODON_INSERTION | G451_L452insFG | c.1350_1355dup | 7 | - | 1 | no | Unknown |
| NON_SYNONYMOUS_CODING | E453K | c.1357G>A | 7 | - | 1 | yes | GOF |
| SPLICE_SITE_DONOR | NA | c.1404+1G>A | - | 8 | 1 | no | Unknown |
| SPLICE_SITE_ACCEPTOR | NA | c.1540-2A>T | - | 9 | 1 | no | Unknown |
| STOP_GAINED | R537* | c.1609C>T | 9 | - | 1 | no | Unknown |
| NON_SYNONYMOUS_CODING | Q546K | c.1636C>A | 9 | - | 1 | yes | GOF |
| NON_SYNONYMOUS_CODING | Q546E | c.1636C>G | 9 | - | 1 | yes | GOFP |
| NON_SYNONYMOUS_CODING | Q546P | c.1637A>C | 9 | - | 1 | yes | GOF |
| NON_SYNONYMOUS_CODING | Q546R | c.1637A>G | 9 | - | 1 | yes | GOF |
| SPLICE_SITE_DONOR | NA | c.1911+1G>A | - | 12 | 1 | no | Unknown |
| NON_SYNONYMOUS_CODING | S66T | c.196T>A | 1 | - | 1 | no | Unknown |
| NON_SYNONYMOUS_CODING | E726K | c.2176G>A | 13 | - | 1 | yes | GOF |
| NON_SYNONYMOUS_CODING | E784K | c.2350G>A | 15 | - | 1 | no | Unknown |
| NON_SYNONYMOUS_CODING | D806N | c.2416G>A | 15 | - | 1 | no | Unknown |
| NON_SYNONYMOUS_CODING | E81K | c.241G>A | 1 | - | 1 | yes | Unknown |
| NON_SYNONYMOUS_CODING | R832Q | c.2495G>A | 16 | - | 1 | no | Unknown |
| NON_SYNONYMOUS_CODING | D915N | c.2743G>A | 18 | - | 1 | no | Unknown |
| NON_SYNONYMOUS_CODING | R93W | c.277C>T | 1 | - | 1 | yes | GOF |
| NON_SYNONYMOUS_CODING | L956F | c.2868G>T | 19 | - | 1 | no | Unknown |
| NON_SYNONYMOUS_CODING | Y1021H | c.3061T>C | 20 | - | 1 | yes | Unknown |
| STOP_GAINED | R1023* | c.3067C>T | 20 | - | 1 | no | Unknown |
| NON_SYNONYMOUS_CODING | R1023Q | c.3068G>A | 20 | - | 1 | yes | Unknown |
| NON_SYNONYMOUS_CODING | N1044D | c.3130A>G | 20 | - | 1 | yes | Unknown |
| NON_SYNONYMOUS_CODING | H1047N | c.3139C>A | 20 | - | 1 | yes | Unknown |
| NON_SYNONYMOUS_CODING | H1047L | c.3140A>T | 20 | - | 1 | yes | GOF |
| NON_SYNONYMOUS_CODING | G106V | c.317G>T | 1 | - | 1 | yes | GOF |
| NON_SYNONYMOUS_CODING | N107I | c.320A>T | 1 | - | 1 | yes | Unknown |
| CODON_DELETION | L113del | c.337_339del | 1 | - | 1 | yes | Unknown |
| NON_SYNONYMOUS_CODING | R115Q | c.344G>A | 1 | - | 1 | yes | Unknown |
| NON_SYNONYMOUS_CODING | G118D | c.353G>A | 2 | - | 1 | yes | GOF |
| NON_SYNONYMOUS_CODING | C255Y | c.764G>A | 3 | - | 1 | no | Unknown |
| NON_SYNONYMOUS_CODING | K264E | c.790A>G | 3 | - | 1 | no | Unknown |
| NON_SYNONYMOUS_CODING | S268C | c.802A>T | 3 | - | 1 | no | Unknown |
| NON_SYNONYMOUS_CODING | S295Y | c.884C>A | 4 | - | 1 | no | Unknown |
| NON_SYNONYMOUS_CODING | M30I | c.90G>T | 1 | - | 1 | no | Unknown |

## **Table S2.** **Variants identified in PTEN (NM_000314**) annotated with exon location, number of times each variant was identified, and functional classification based on the JAX Clinical Knowledgebase (CKB CORE, <https://ckb.genomenon.com/>; accessed 31.03.2026).^4^ For *PTEN* mutations not present in the database, protein truncating mutations were regarded as “loss of function - predicted (LOFP)”, the remainder as “unknown”. LOF = Loss of function, LOFP = Loss of function – predicted

| **Mutation** | **Protein change** | **Transcript change** | **Coding exon** | **Intron** | **Count** | **Variant in database** | **Functional classification** |
| --- | --- | --- | --- | --- | --- | --- | --- |
| SPLICE_SITE_ACCEPTOR | NA | c.802-3dup | - | 7 | 4 | no | Unknown |
| NON_SYNONYMOUS_CODING | R130Q | c.389G>A | 5 | - | 2 | yes | LOF |
| FRAME_SHIFT | R335Dfs*9 | c.1003del | 8 | - | 1 | no | LOFP |
| NON_SYNONYMOUS_CODING | P354L | c.1061C>T | 9 | - | 1 | no | Unknown |
| NON_SYNONYMOUS_CODING | E373K | c.1117G>A | 9 | - | 1 | no | Unknown |
| NON_SYNONYMOUS_CODING | S380F | c.1139C>T | 9 | - | 1 | no | Unknown |
| STOP_GAINED | Q399* | c.1195C>T | 9 | - | 1 | yes | Unknown |
| NON_SYNONYMOUS_CODING | K6N | c.18A>C | 1 | - | 1 | yes | Unknown |
| FRAME_SHIFT | G143Afs*4 | c.428del | 5 | - | 1 | yes | LOFP |
| STOP_GAINED | K147* | c.439A>T | 5 | - | 1 | yes | LOFP |
| FRAME_SHIFT | E157Gfs*23 | c.469dup | 5 | - | 1 | no | LOFP |
| STOP_GAINED | E201* | c.601G>T | 6 | - | 1 | yes | LOFP |
| NON_SYNONYMOUS_CODING | Q219R | c.656A>G | 7 | - | 1 | no | Unknown |
| STOP_GAINED | L23* | c.68T>G | 1 | - | 1 | no | LOFP |
| FRAME_SHIFT | K267Rfs*9 | c.800del | 7 | - | 1 | yes | LOFP |
| FRAME_SHIFT | D268Gfs*30 | c.800dup | 7 | - | 1 | yes | LOFP |
| NON_SYNONYMOUS_CODING | H272P | c.815A>C | 8 | - | 1 | yes | LOFP |
| FRAME_SHIFT | T319* | c.955_958del | 8 | - | 1 | yes | LOF |
| FRAME_SHIFT | N323Mfs*21 | c.968del | 8 | - | 1 | yes | LOFP |
| CODON_DELETION | I33del | c.97_99del | 2 | - | 1 | yes | LOFP |
| FRAME_SHIFT | N329Kfs*14 | c.987_990del | 8 | - | 1 | yes | LOFP |

## **Table S3**. **Disease-free survival by biomarker status and randomised treatment for oncogenic variants of *PIK3CA* and *PTEN* mutations and for *PIK3CA* hot spot mutations.**

| Known or likely oncogenic variant mutations^1^ | Patients  N | | Events  n | | HR (95% CI) | % DFS – 5 years (95% CI) | |
| --- | --- | --- | --- | --- | --- | --- | --- |
|  | Placebo | Aspirin | Placebo | Aspirin |  | Placebo | Aspirin |
| *PIK3CA* | 29 | 26 | 7 | 6 | 0.90 (0.30, 2.67) | 76 (62, 93) | 75 (60, 95) |
| *PIK3CA* exon 9 / 20 | 25 | 18 | 7 | 4 | 0.73 (0.21, 2.49) | 72 (56, 92) | 77 (59, 100) |
| *PTEN* | 7 | 7 | 0 | 1 | Undefined | 100 (-, 100) | 83 (58, 100) |
| *PIK3CA* or *PTEN* | 35 | 31 | 7 | 7 | 1.10 (0.39, 3.14) | 80 (68, 94) | 76 (62, 93) |
| Hot spot mutations^2^ |  |  |  |  |  |  |  |
| *PIK3CA* exon 9/ 20 | 26 | 18 | 7 | 4 | 0.76 (0.22, 2.61) | 73 (58, 92) | 77 (59, 100) |
|  |  |  |  |  |  |  |  |

^1^ Based on classification used by Nowak et al.^13^

^2^ Based on the ALASCCA classification for Group A patients.^7^

**Table S4**. **Association of MMR/MSI status and *PIK3CA* mutations.^1^**

|  |  | | MMR/MSI status | | |  | |  | |
| --- | --- | --- | --- | --- | --- | --- | --- | --- | --- |
|  | | **Total** | | **MSS** | **MSI** | | Unknown | | p-value^2^ |
| *PIK3CA* Mutation* | |  | |  |  | |  | |  |
| Yes | | 69 | | 50 (75%) | 17 (25%) | | 2 | | 0.001 |
| No | | 328 | | 288 (90%) | 32 (10%) | | 8 | |  |
| *PIK3CA* mutation (Exon 9, 20) | |  | |  |  | |  | |  |
| Yes | | 45 | | 33 (77%) | 10 (23%) | | 2 | | 0.05 |
| No | | 352 | | 305 (89%) | 39 (11%) | | 8 | |  |

## ^1^ n (%) ^2^ Pearson’s Chi-squared test

**Table S5**. **Association of Cox-2 Overexpression and PI3K-related mutations.^1^**

|  | Cox-2 Overexpression | | |  | |
| --- | --- | --- | --- | --- | --- |
|  | | **Positive**  N = 307 | **Negative**  N = 137 | | p-value^2^ |
| *PIK3CA* Mutation | | 21 (18%) | 45 (17%) | | 0.9 |
| *PIK3CA* mutation (Exon 9, 20) | | 12 (10%) | 31 (12%) | | 0.7 |
| *PTEN* mutation | | 3 (4.2%) | 16 (8.0%) | | 0.3 |

^1^ n (%) ^2^ Pearson’s Chi-squared test

## **Table S6**. **Disease-free survival by PIK3CA mutation status and randomised treatment: sensitivity analysis with patients with unknown PIK3CA other exon mutation due to failed NGS reported separately.**

| *PIK3CA* mutation | Patients  N | | Events  n | | HR (95% CI) |
| --- | --- | --- | --- | --- | --- |
|  | Placebo | Aspirin | Placebo | Aspirin |  |
| Yes | 34 | 35 | 8 | 8 | 0.93 (0.35, 2.47) |
| No | 115 | 122 | 27 | 26 | 0.90 (0.53, 1.54) |
| Unknown | 38 | 53 | 6 | 11 | 1.38 (0.51, 3.73) |

## **Table S7** **Disease-free survival by COX-2 Expression (ordered categories) and randomised group.**

| COX-2 Expression Group^1^ | Patients^2^  N | | Events  n | | HR (95% CI^3^ |
| --- | --- | --- | --- | --- | --- |
|  | Placebo | Aspirin | Placebo | Aspirin |  |
| Absent | 11 | 10 | 2 | 4 | 2.60(0.47, 14.2) |
| Weak | 58 | 58 | 14 | 10 | 0.69 (0.31, 1.56) |
| Moderate | 72 | 75 | 18 | 15 | 0.80 (0.40, 1.59) |
| Strong | 65 | 95 | 10 | 19 | 1.36 (0.63, 2.92) |
|  |  |  |  |  |  |

^1^ COX-2 overexpression correspond to moderate and/or strong expression groups.

^2^ m-ITT population with available Tissue

^3^ HR = Hazard Ratio; CI = Confidence Interval

##

## **Table S8 Completed randomised trials of adjuvant aspirin in patients with CRC harbouring PI3K-related mutations**

| Trial (reference) | Years recruited | Year 1^st^ published | Women (%) | White (%) | Cancer type | Treatment | Given with or after any adjuvant chemotherapy | Treatment / follow-up (yrs) | Patients | | Patients / DFS events with PI3K alterations | |
| --- | --- | --- | --- | --- | --- | --- | --- | --- | --- | --- | --- | --- |
|  |  |  |  |  |  |  |  |  | Placebo | Aspirin | *PIK3CA* exon 9,20 | 0ther PI3K related |
| SAKK 41/13^6^ | 2016-2020 | 2025 | 43 | 94 | Stage II / III colon | Asp 100 mg v placebo | With | 3 / 5 yrs | 38 | 74 | 112 /19 | 0 /0 |
| ALASCCA^7^ | 2016-2021 | 2025 | 52 | * | Stage II/III colon or rectal | Asp 160 mg v placebo | With | 3 / 3 yrs | 313 | 313 | 314 / 49 | 312 / 54 |
| ASCOLT^8^ | 2009-2021 | 2025 | 42 | 28** | Stage II/III colon or rectal | Asp 200mg v placebo | After | 3 / 5 yrs | 759 | 791 | 45 /11 | 41/ 6 |

* Race was not collected, but recruitment only from Nordic countries – so the vast majority were white ** Among patients included in ASCOLT TR 71% were white.

## **Table S9 Disease-free survival by sex and randomised group.**

| Patient Group | Patients  N | | Events  n (%) | | HR (95% CI) |
| --- | --- | --- | --- | --- | --- |
| All patients | Placebo | Aspirin | Placebo | Aspirin |  |
| Women | 83 | 117 | 20 (24%) | 25 (21%) | 0.87 (0.48, 1.57) |
| Men | 135 | 130 | 26 (19%) | 25 (19%) | 1.01 (0.58, 1.75) |
| Patients with *PIK3CA* Mutation | | | | | |
| Women | 14 | 16 | 4 (29%) | 5 (31%) | 1.02 (0.27, 3.78) |
| Men | 20 | 19 | 4 (20%) | 3 (16%) | 0.77 (0.17, 3.43) |

HR - Hazard ratio; CI – Confidence Interval; DFS – Disease-free survival

# Supplementary Figures

***
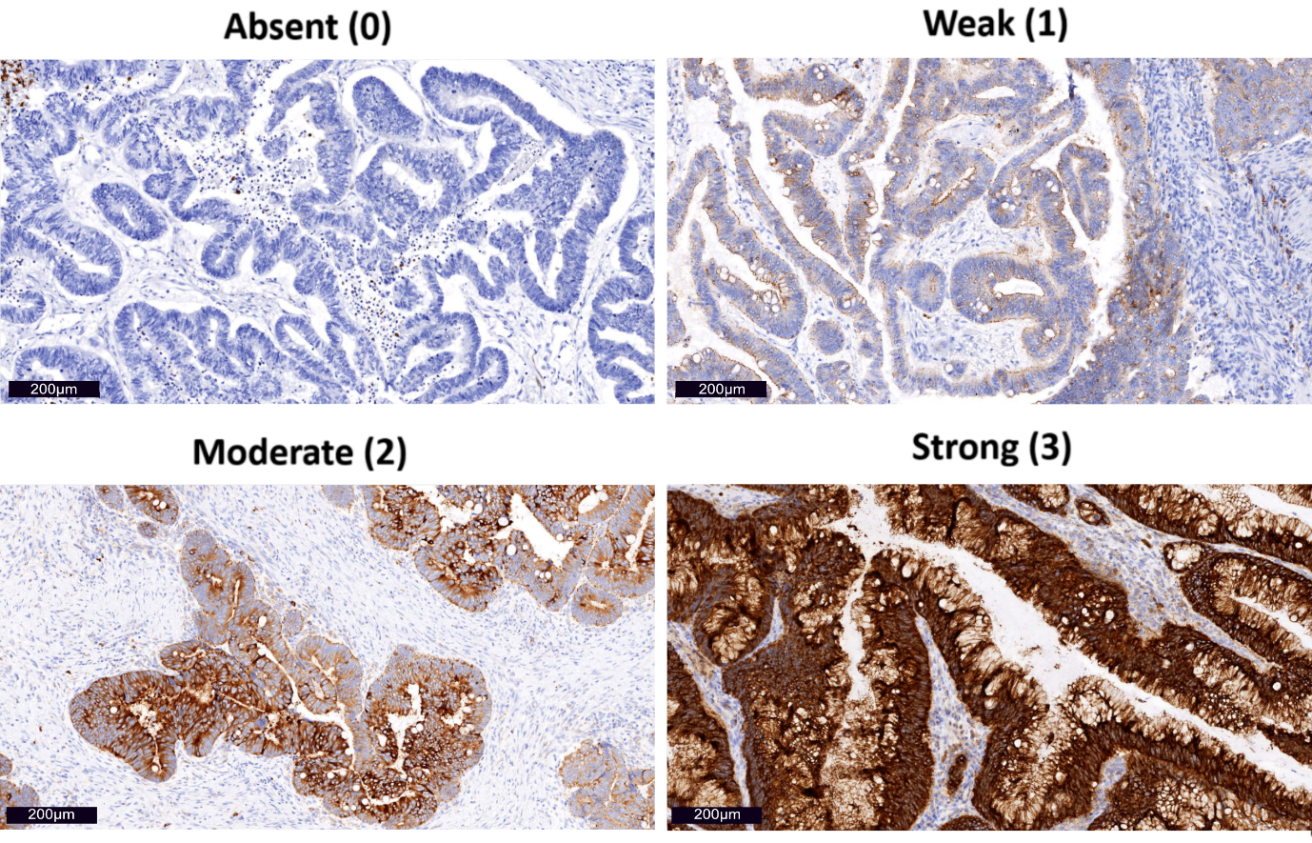
***

## **Figure S1.** COX-2 immunohistochemistry on colorectal cancer sections illustrating absent (0), weak (1), moderate (2) and strong (3) staining. Scale bars = 200 μM.


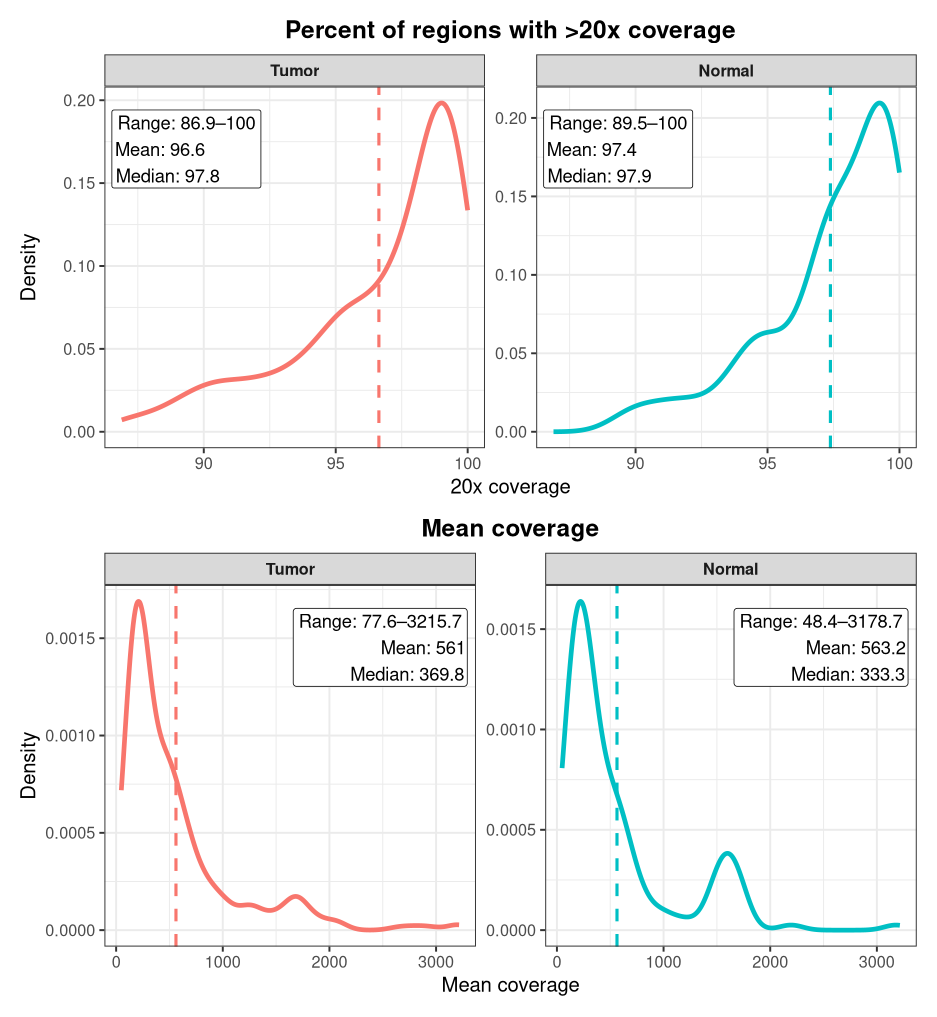


**Figure S2.** Target region coverage metrics for tumour and normals samples assessed by next-generation sequencing including percent of regions with >20x coverage and mean target coverage; dashed lines indicate mean values.

**PIK3CA hot spot mutations exon 9/ 20**


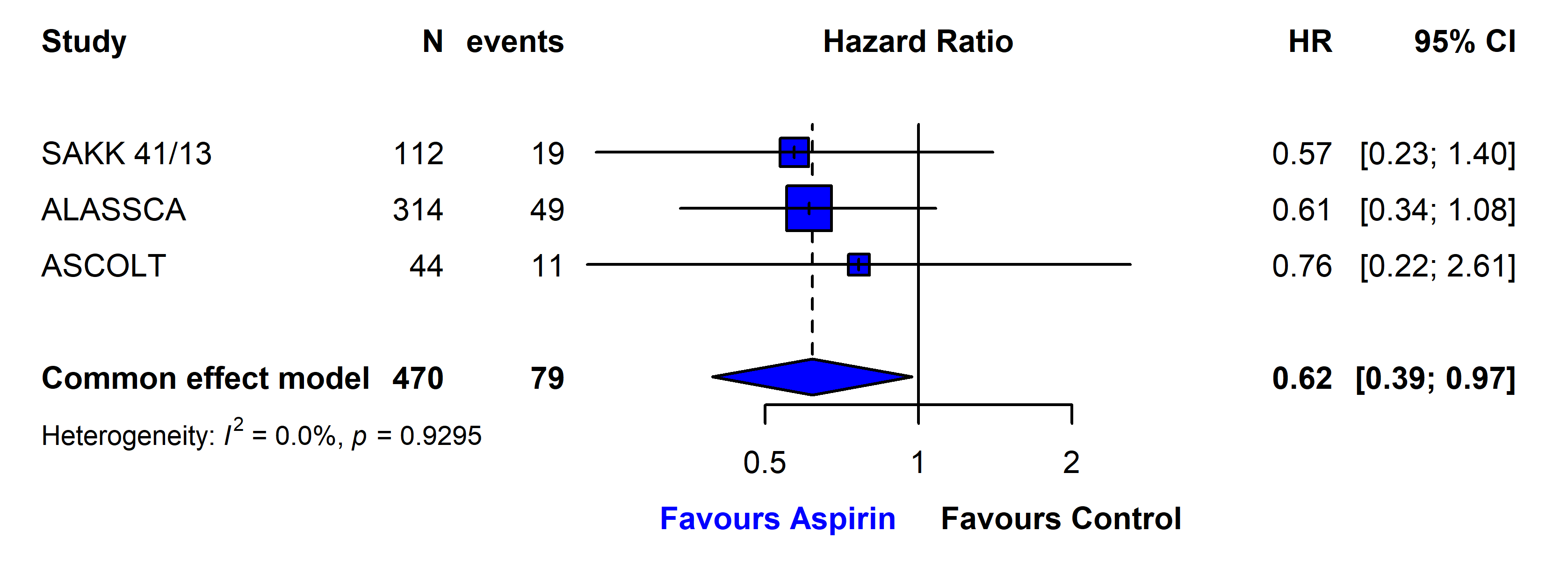


**Other PI3K (other PIK3CA, PIK3R1, PTEN)**


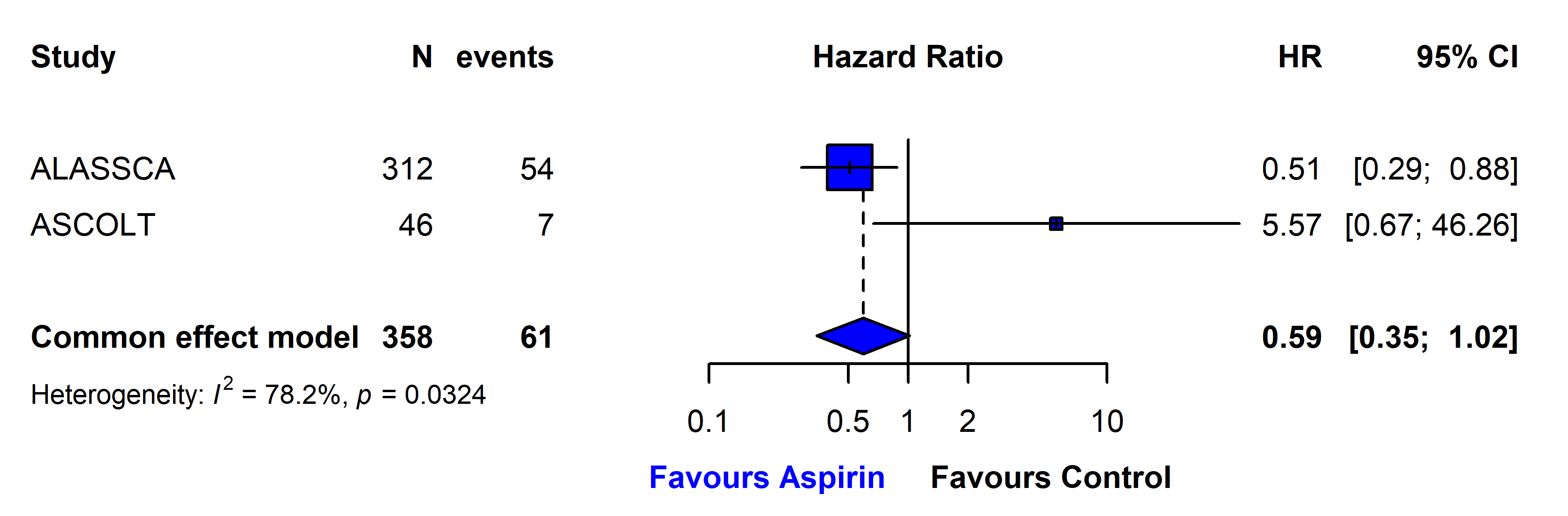


**Any PI3K alterations (PIK3CA, PIK3R1, PTEN)**


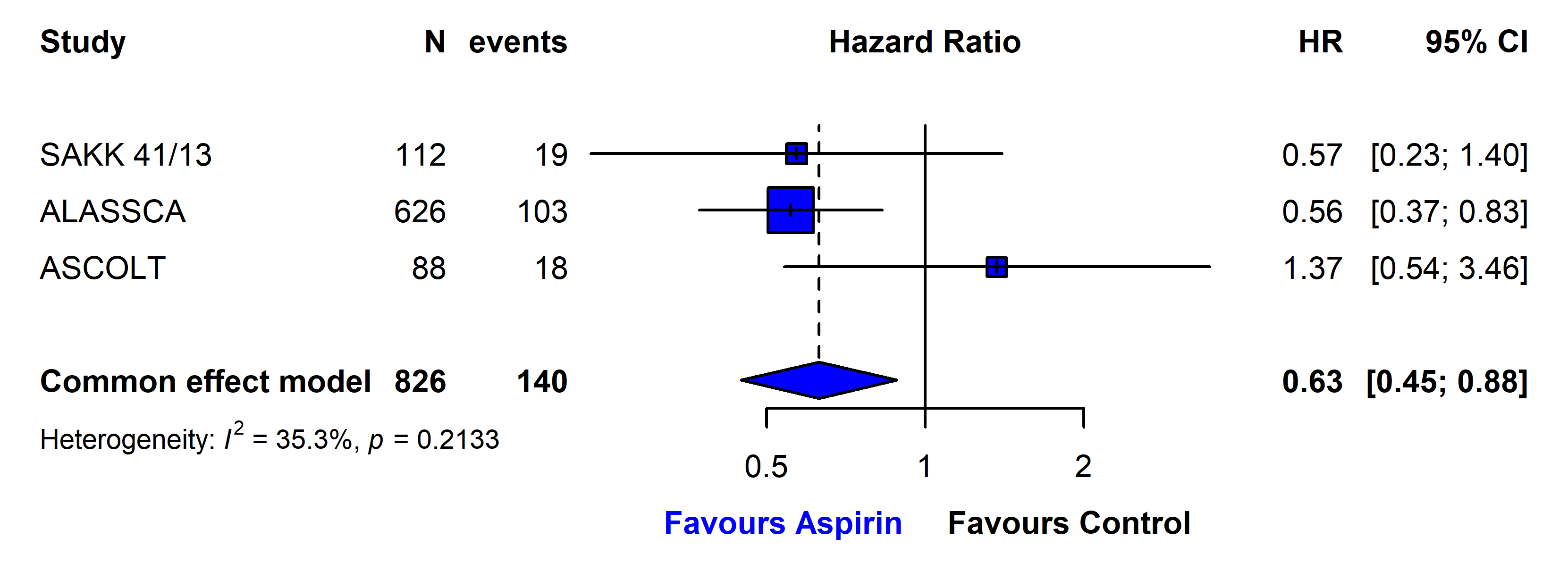


## **Figure S3.** Treatment effects of adjuvant aspirin versus placebo on **disease-free survival** in SAKK 41/13,^6^ ALASCCA^7^ and ASCOLT^8^ trials in patients with somatic alterations of *PI3K* pathway: with ASCOLT data as per the ALASCCA criteria:^7^ Group A for *PIK3CA* exon 9,20 limited to hot spot mutations and Group B for other PI3K mutations broadened to include nonsense and splice site mutations.

**PIK3CA hot spot mutations exon 9/ 20**


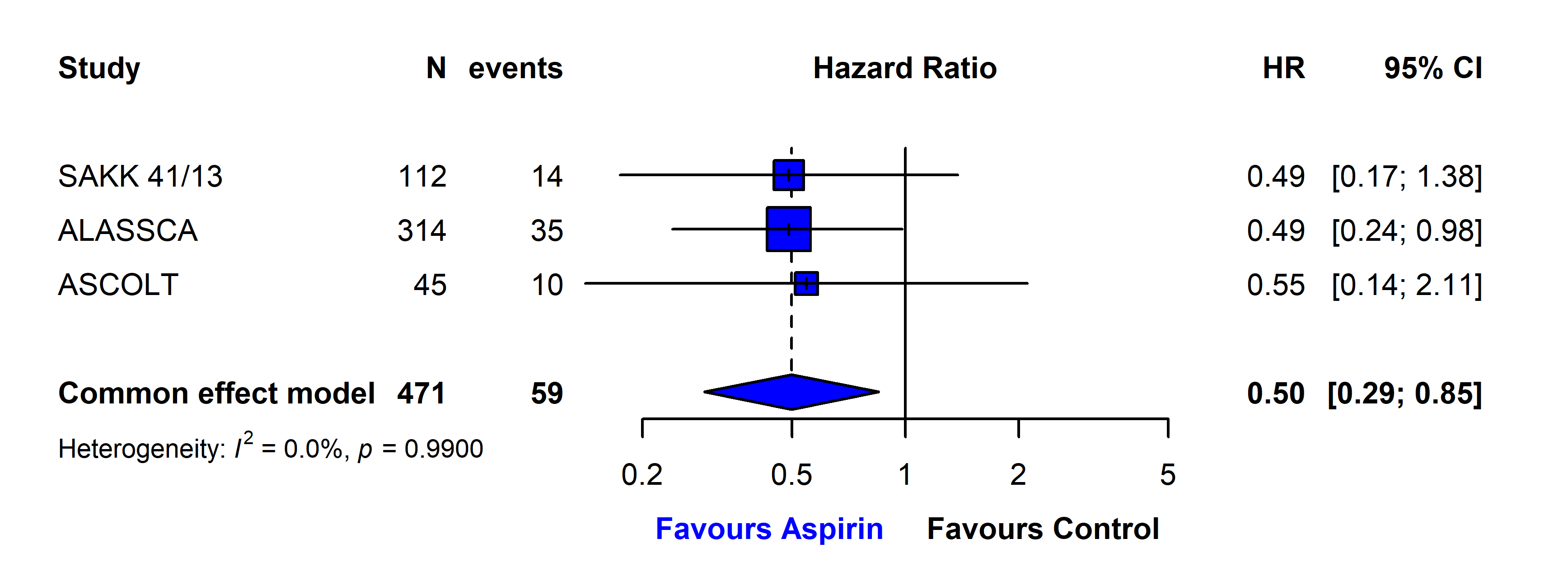


**Other PI3K (other PIK3CA, PIK3R1, PTEN)**


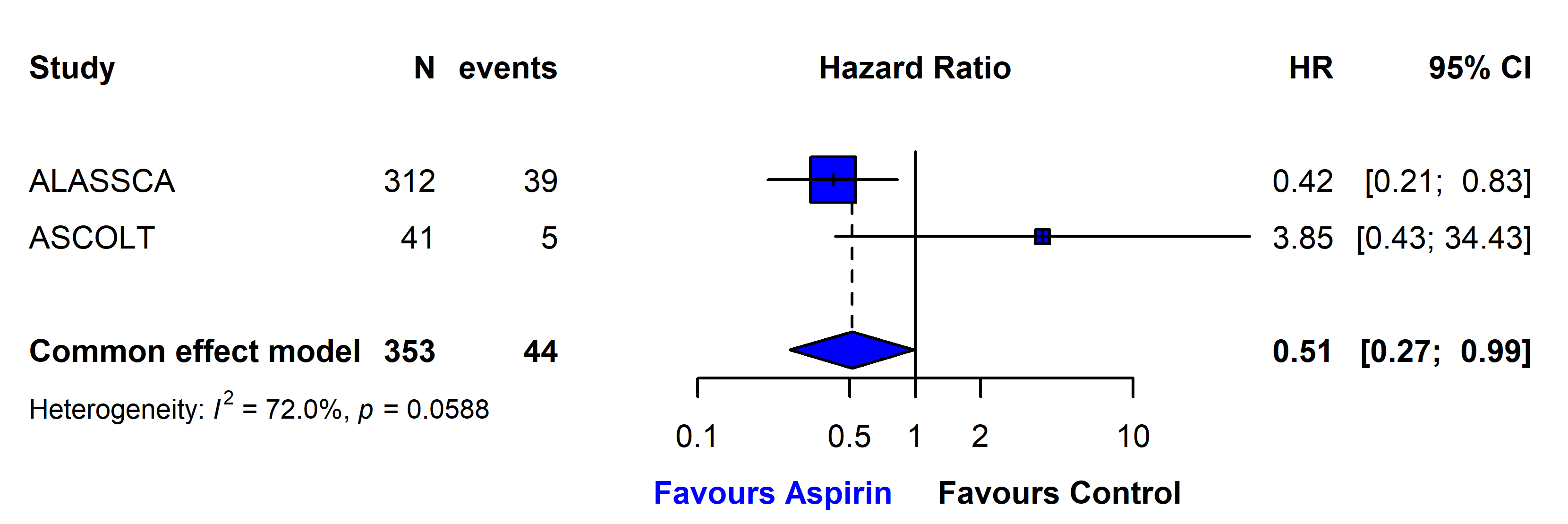


**Any PI3K alterations (PIK3CA, PIK3R1, PTEN)**


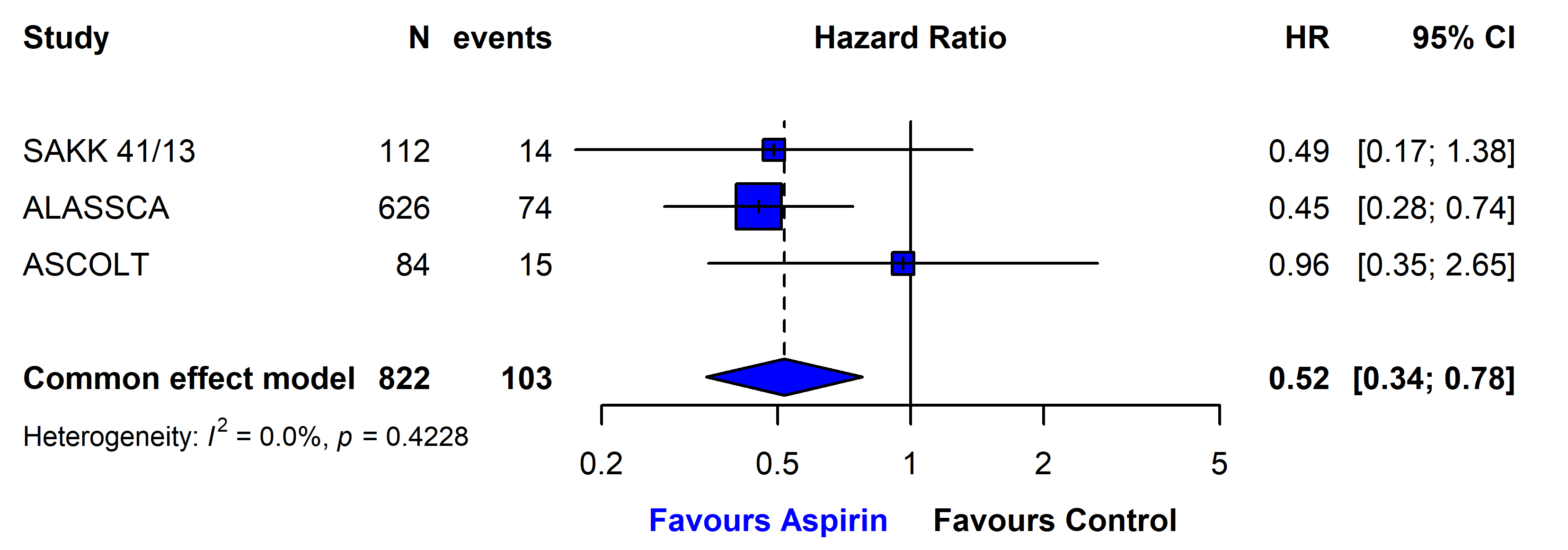


## **Figure S4.** Treatment effects of adjuvant aspirin versus placebo on **time to recurrence** in SAKK 41/13,^6^ ALASCCA^7^ and ASCOLT^8^ trials in patients with somatic alterations of *PI3K* pathway.

**PIK3CA hot spot mutations exon 9/ 20**


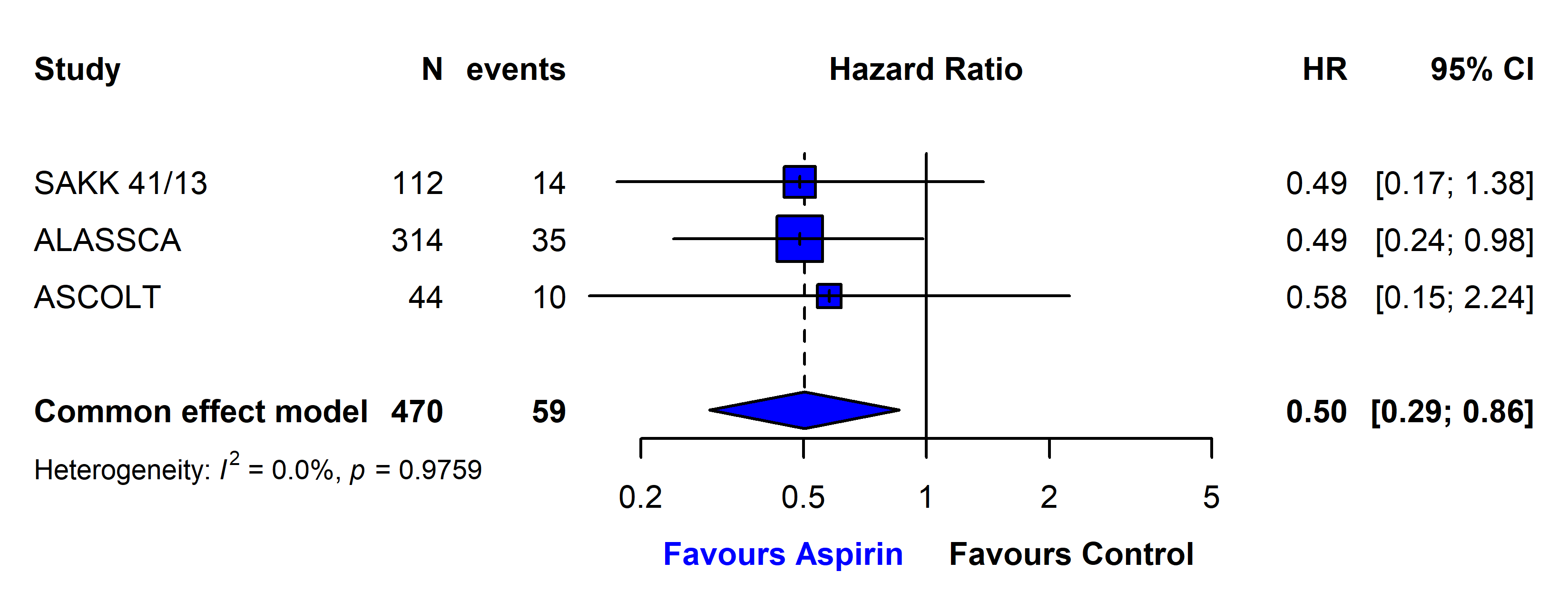


**Other PI3K (other PIK3CA, PIK3R1, PTEN)**


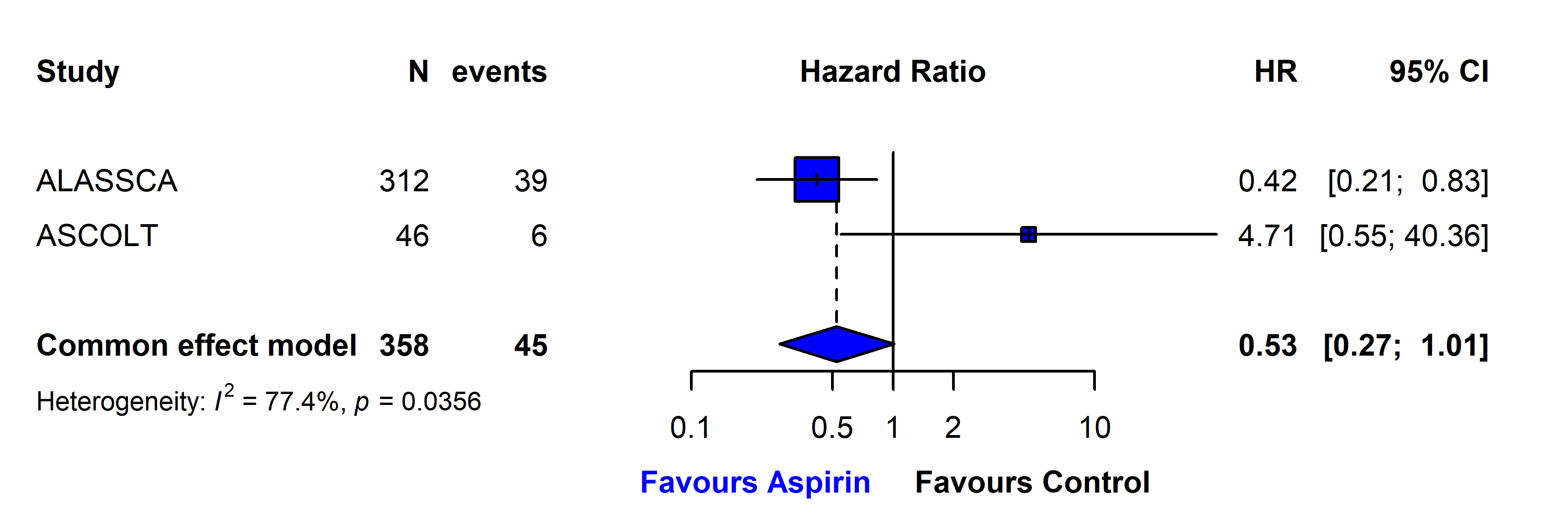


**Any PI3K alterations (PIK3CA, PIK3R1, PTEN)**


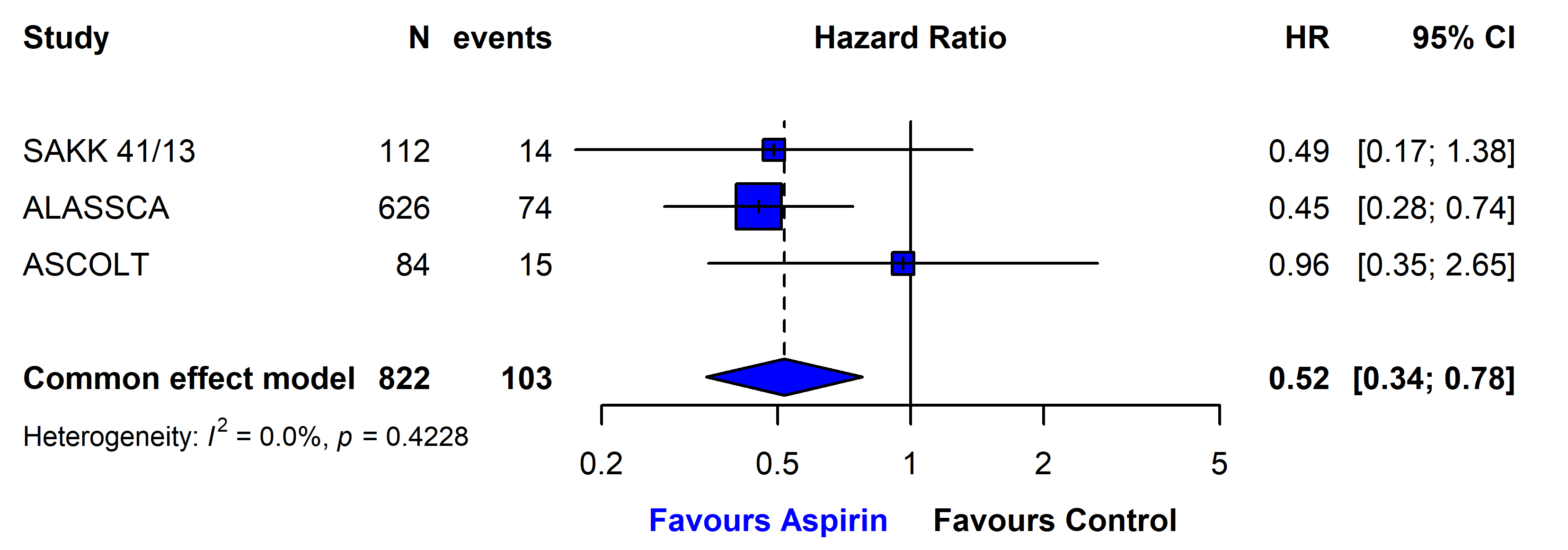


## **Figure S5.** Treatment effects of adjuvant aspirin versus placebo on **time to recurrence** in SAKK 41/13,^6^ ALASCCA^7^ and ASCOLT^8^ trials in patients with somatic alterations of *PI3K* pathway. with ASCOLT data as per the ALASCCA criteria:7 Group A for PIK3CA exon 9,20 limited to hot spot mutations and Group B for other PI3K mutations broadened to include nonsense and splice site mutations.

# Supplementary References

1. Chan AT, Ogino S, Fuchs CS. Aspirin and the risk of colorectal cancer in relation to the expression of COX-2. N Engl J Med. 2007; 356:2131-42.
2. Chan AT, Ogino S, Fuchs CS. Aspirin use and survival after diagnosis of colorectal cancer. JAMA. 2009; 302:649-58.
3. Ogino S, Kirkner GJ, Nosho K, et al. Cyclooxygenase-2 expression is an independent predictor of poor prognosis in colon cancer. Clin Cancer Res. 2008; 14:8221-7.
4. Patterson SE, Statz CM, Yin T, Mockus SM. Utility of the JAX Clinical Knowledgebase in capture and assessment of complex genomic cancer data. NPJ Precis Oncol. 2019; 3:2.
5. Burdett S, Fisher D, Tierney J, Meade A, Nankivell M, Langley R. Aspirin after radical therapy for colorectal cancer: a prospective meta-analysis. PROSPERO 2024 Available from <https://www.crd.york.ac.uk/PROSPERO/view/CRD42023453156>
6. Güller U, Hayoz S, Horber D, et al. Adjuvant Aspirin Treatment in PIK3CA Mutated Colon Cancer Patients: The SAKK 41/13 - Prospective Randomized Placebo-Controlled Double-Blind Trial. Clin Cancer Res. 2025 Mar 11: 10.1158/1078-0432.CCR-24-4048. doi: 10.1158/1078-0432.CCR-24-4048
7. Martling A, Myrberg IH, Nilbert M, et al, for the ALASCCA Study Group. Low-Dose Aspirin for PI3K-Altered Localized Colorectal Cancer. N Engl J Med. 2025;393(11):1051-1064.
8. Chia JWK, Segelov E, Deng Y, et al. Aspirin after completion of standard adjuvant therapy for colorectal cancer (ASCOLT): an international, multicentre, phase 3, randomised, double-blind, placebo-controlled trial. Lancet Gastroenterol Hepatol 2025; 10: 198–209
9. Borenstein M, Hedges LV, Higgins JPT, Rothstein HR. Introduction to Meta-analysis. Wiley: Chichester, 2009
10. DerSimonian R, Laird NM. Meta-analysis in clinical trials, Control. Clin. Trials 7 (1986) 177–188.
11. Jackson D. Confidence intervals for the between-study variance in random effects meta-analysis using generalised Cochran heterogeneity statistics. Res Synth Methods. 2013 Sep;4(3):220-9.
12. Higgins JPT, Thompson SG. Quantifying heterogeneity in a meta-analysis. Statistics in Medicine 2002; 21:1539-1558.
13. Nowak JA, Twombly T, Ma C, et al. Improved Survival with Adjuvant Cyclooxygenase 2 Inhibition in PIK3CA-Activated Stage III Colon Cancer: CALGB/SWOG 80702 (Alliance). J Clin Oncol. 2024; 42:2853-2859.
