# Supplementary material for: Adjuvant aspirin for colorectal cancer with PIK3CA-mutated and COX-2 overexpressed tumours: the ASCOLT translational research study and meta-analysis
Source: eBioMedicine. 2026 Jul 20;130:106389. doi: 10.1016/j.ebiom.2026.106389 (PMC13393707; doi:10.1016/j.ebiom.2026.106389)
Supplement: ASCOLT Protocol [file mmc2.pdf]

ICR02 / ASCOLT

Aspirin for Dukes C and High Risk Dukes B Colorectal Cancers

An International, Multi-centre, Double Blind, Randomised Placebo Controlled Phase III Trial

Version 7.0: 09 May 2018

TRIAL OUTLINE

Aspirin for Dukes C and High Risk Dukes B Colorectal Cancers - An International, multi-centre, double blind, randomised Placebo controlled Phase III Trial

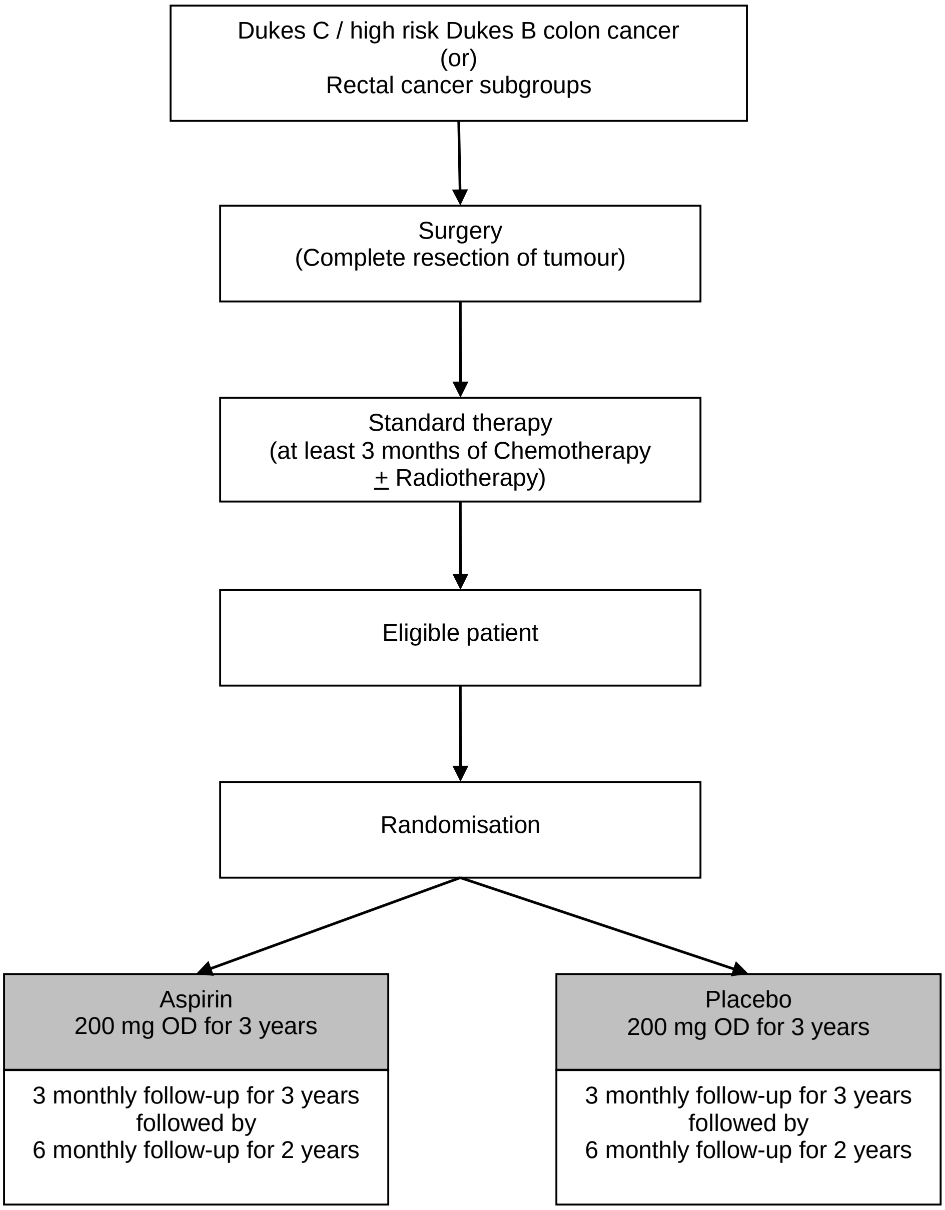

TRIAL MANAGEMENT GROUP

Study Co-Chair

Dr John Chia Whay Kuang	Division of Medical Oncology<br>National Cancer Centre<br>11 Hospital Drive<br>Singapore 169610
A/Prof Toh Han Chong	Division of Medical Oncology<br>National Cancer Centre<br>11 Hospital Drive<br>Singapore 169610
Dr Raghib Ali	Director, Public Health Research Center<br>New York University Abu Dhabi<br>Saadiyat Island. PO Box 129188<br>Abu Dhabi. UAE
Prof Eva Segelov	Professor/Director of Oncology<br>Monash Health and Monash University<br>Lvl 7, MHTP building, Monash Health 246 Clayton Rd<br>Clayton VIC 3168 Australia

Trial Project Management

Project Manager	Division of Medical Oncology<br>National Cancer Centre 11 Hospital Drive<br>Singapore 169610

Trial Biostatisticians

SCRI Statistician	Singapore Clinical Research Institute<br>31 Biopolis Way, Nanos #02-01<br>Singapore 138669

Trial Study Pharmacist

National Cancer Centre Trial Pharmacist	National Cancer Centre<br>Oncology Pharmacy Level 3<br>11, Hospital Drive<br>Singapore 169610

TRIAL STEERING COMMITTEE

Independent Member

Professor Pierce Chow	Division of Surgical Oncology<br>National Cancer Centre<br>11 Hospital Drive<br>Singapore 169610
Professor Peter M Rothwell	Department of Clinical Neurology<br>University of Oxford<br>Level 6, West Wing, John Radcliffe Hospital<br>Oxford, OX3 9DU, UK

Non-Independent Members

Dr John Chia Whay Kuang	Division of Medical Oncology<br>National Cancer Centre<br>11 Hospital Drive<br>Singapore 169610
A/Prof Toh Han Chong	Division of Medical Oncology<br>National Cancer Centre<br>11 Hospital Drive<br>Singapore 169610
Dr Raghib Ali	Director, Public Health Research Center<br>New York University Abu Dhabi<br>Saadiyat Island. PO Box 129188<br>Abu Dhabi. UAE
Prof David J Kerr	Department of Clinical Pharmacology<br>University of Oxford<br>Old Road Campus Research Building<br>Oxford, OX3 7DQ
Prof Eva Segelov	Professor/Director of Oncology<br>Monash Health and Monash University<br>Lvl 7, MHTP building, Monash Health 246 Clayton Rd<br>Clayton VIC 3168 Australia
Prof R John Simes	Director<br>NHMRC Clinical Trials Centre<br>Medical Foundation Building<br>University of Sydney NSW 2006 Australia
Estelle Foo Mei Jye	Project Manager<br>Division of Medical Oncology<br>National Cancer Centre 11 Hospital Drive<br>Singapore 169610

Observer, Nonvoting member

Elmar Detering<br>Medical Affairs Physician (MD, PhD)<br>Global Medical Lead Aspirin (low dose; CRC)	Bayer AG<br>Medical Affairs TA Pulmonology & Established Products.<br>Medical Affairs & Pharmacovigilance, Pharmaceuticals<br>Building S101 / 07 / 156<br>Muellerstrasse 178<br>13353 Berlin, Germany

Data Monitoring Committee

Prof Ian F. Tannock	Emeritus Professor of Medical Oncology,<br>Princess Margaret Cancer Centre<br>610 University Avenue,<br>Toronto, ON M5G 2M9, Canada
Prof Andrew Chan	Massachusetts General Hospital / Program Director of Gastroenterology Training Program
Dr Sharon Love	Centre for Statistics in Medicine<br>Wolfson College Annexe<br>Linton Road, Oxford<br>OX2 6UD, UK

SUPPORTED BY GRANT FUNDING

SingHealth Foundation

National Medical Research Council, Singapore

National Cancer Centre Research Fund

Rising Tide Foundation

Lee Foundation

Lee Kim Tah Foundation

PHARMACEUTICAL SUPPORT

Bayer AG (Provided study drugs)

The list of participating sites will be supplied in the form of supplementary documents as requested.

This protocol is intended for use by the investigators and collaborators of this study. It is otherwise available for information only. No part of this protocol may be reproduced or distributed in any form or by any means, or stored in a database or retrieval system without the prior written permission of steering committee.

TABLE OF CONTENTS

LIST OF ABBREVIATIONS 1

SUMMARY 2

1. INTRODUCTION 6

1.1. Background 6
1.2. Adjuvant agents for colorectal cancer 7
1.3. Failure of targeted Agents 8
1.4. NSAIDS as adjuvant agents 9
1.5. Aspirin's Secondary Role – Prevention of new polyps 11
1.6. Primary Prevention of Colorectal Cancer 12
1.7. Preclinical data and scientific rationale 14
1.8. Optimal biologically effective dose for Aspirin 15

2. STUDY OBJECTIVE 16

3. STUDY ENDPOINTS 16

4. TRIAL DESIGN 16

5. ELIGIBILITY CRITERIA 17

5.1. Inclusion Criteria 17
5.2. Exclusion Criteria 18

6. RANDOMISATION 18

7. STUDY DRUG 19

7.1. How to take the study drug 19
7.2. Supply and Resupply of study drug 19
7.3. Description and contents of study drug and placebo drug 19
7.4. Packaging information 19
7.5. Storage information 20
7.6. Study drug labelling and handling 20
7.7. Compliance of study drug 20
7.8. Anticipated side effects of Aspirin 20

8. BLINDING 21

8.1. Blinding 21
8.2. Code-break envelopes 21
8.3. Unblinding 21

9. TREATMENT SCHEDULE 21

9.1. Treatment groups 21
9.2. Treatment modification 22
9.3. Treatment discontinuation 23
9.4. Study Withdrawal 24

10. CONCOMITANT/CONTRAINDICATED MEDICATIONS 24

11. ASSESSMENTS AND FOLLOW-UP 24

11.1. Screening 24
11.2. Baseline 25
11.3. 3-monthly assessments 25

11.4. 6-monthly assessments ... 26
11.5. Unscheduled visits ... 26
11.6. Assessment of recurrence and salvage chemotherapy ... 26
11.7. Assessment of Medical Events ... 27
11.8 Post Month 60 – Clinical Data ... 27

12 STATISTICAL CONSIDERATIONS ... 27

12.1 Sample size calculation ... 27
12.2 Statistical analysis ... 28
12.3 Interim analysis ... 29

13 SAFETY ... 29

13.1 Definitions ... 30
13.2 Reporting of Adverse Event ... 31
13.3 Reporting of Death ... 31

14 SUB-STUDIES (For Singapore and Selected Sites Only) ... 32

14.1 Blood Samples ... 32
14.1 Biomarkers, protein, lipid and leukocyte profiling ... 32
14.2 Germline DNA ... 32
14.3 Stool Samples ... 32
14.4 Urine Samples. ... 32
14.5 Archival tumour specimens ... 32

15 DIRECT ACCESS TO SOURCE DATA ... 33

16 QUALITY CONTROL AND QUALITY ASSURANCE ... 33

17 ETHICAL CONSIDERATIONS ... 33

18 INSURANCE ... 33

19 PUBLICATION POLICY ... 33

20 STUDY ORGANISATION ... 34

21 ARCHIVING ... 34

REFERENCES ... 35

APPENDIX 1 – TNM AND DUKES STAGING FOR COLORECTAL CANCER ... 39

APPENDIX 2 – ECOG PERFORMANCE STATUS ... 41

APPENDIX 3 – STUDY VISIT SCHEDULES & ASSESSMENTS ... 42

APPENDIX 4 – STUDY DRUG LABEL ... 43

LIST OF ABBREVIATIONS

5FU	5-Fluorouracil
AE	Adverse Event
ALP	Alkaline phosphatise
ALT	Alanine transaminase
ANC	Absolute neutrophil count
AST	Aspartate transaminase
BP	Blood pressure
CEA	Carcinoembryonic antigen
CIOMS	Council for International Organisations of Medical Sciences
CRA	Clinical Research Associate
CT	Computed tomography
DFS	Disease Free Survival
DMC	Data Monitoring Committee
DRE	Digital rectal examination
EC	Ethics Committee
ECG	Electrocardiogram
ECOG	Eastern Cooperative Oncology Group
ERUS	Endorectal ultrasound
FA	Folinic acid
FAP	Familial adenomatous polyposis
FOLFOX	FOLFOX is a chemotherapy regimen for treatment of colorectal cancer, made up of the drugs, folinic acid (FOL), fluorouracil (F) and oxaliplatin (OX)
GCP	Good Clinical Practice
GERD	Gastroesophageal reflux disease
Hb	Haemoglobin
HR	Hazard Ratio
IRB	Institutional Review Board
LFT	Liver function tests
MCRC	Medical Clinical Research Committee
NSAID	Non-steroidal anti-inflammatory drugs
OS	Overall Survival
PGE2	Prostaglandin E2
PPI	Proton pump inhibitor
PR	Pulse rate
RT	Radiation therapy
SCRI	Singapore Clinical Research Institute
SAE	Serious Adverse Event
TCF	T-Cell Factor
TME	Total mesocolon excision
TMG	Trial Management Group
TSC	Trial Steering Committee
VEGF	Vascular Endothelial growth factor

SUMMARY

Protocol Identifier: ASCOLT/ ICR02

Version Number: Version 7.0: 09 May 2018

Title: Aspirin for Dukes C and High Risk Dukes B Colorectal Cancers An International, Multi-centre, Double Blind, Randomized Placebo Controlled Phase III Trial

Study Phase: III

Rationale:

The utility of Aspirin and NSAIDs in preventing cancer, especially colon cancer is the subject of intense pre-clinical and clinical interest and investigation. Whereas high quality evidence (randomized controlled studies) indicates that aspirin is effective in reducing colorectal adenomatous polyps; and numerous studies point towards an ability to prevent colorectal cancer; the role of Aspirin as an adjuvant agent in patients with established cancers remains to be defined. The analysis of a nested cohort study (Nurses Health Study) has suggested that the initiation of Aspirin *after* the diagnosis of colon cancer was able to reduce colorectal cancer specific mortality (HR 0.53, CI 0.33-0.86). Further, a preplanned analysis of patients in the CALGB 89810 adjuvant Irinotecan trial suggested that patients who used aspirin or a cox2 inhibitor regularly had half the risk of recurrence and death. More recently, a Scottish study showed post-diagnosis aspirin use to be associated with a lower risk of death from CRC (HR 0.58, CI 0.45-0.75). Similarly, a Dutch study demonstrated post-diagnosis aspirin use to associate with an improvement in overall survival (HR 0.70, CI 0.57-0.88). Up to date, although this data is strongly supportive of Aspirin's potential role in adjuvant CRC therapy, the findings do not cross the threshold required to change clinical practice – that of a prospective randomized clinical trial. Two large randomized cox-2 inhibitor studies had been undertaken to answer the same question, but were suspended prematurely, due to concerns of cardiovascular toxicity. In contrast, Aspirin is cardio-protective and is one of the most widely used drugs with a well-established safety profile. We hypothesize through this randomized, placebo-controlled adjuvant study, that Aspirin in patients with dukes C or high risk dukes B colorectal cancer (ASCOLT) can improve survival in this patient population over placebo control. If indeed found to be beneficial, because aspirin is cheap and easy to administer, it will positively impact the lives of many individuals in Asia and globally.

Objectives:

Primary Endpoint: Disease Free Survival (DFS)
Secondary Endpoint: Overall Survival (OS) over 5 years

Subjects and Centres: A total of up to 1587 patients will be randomized from multinational centres.

Inclusion Criteria:

- Male or female outpatient of ≥ 18 years of age or ≥ country's legal age for adult consent
- Dukes C colon cancer, high risk Dukes B colon cancer, Dukes B rectal cancer or Dukes C rectal cancer
- Undergone complete resection of primary tumour
- Completed standard therapy (at least 3 months of chemotherapy ± radiotherapy)
- Within 120 days of completion of standard therapy
- (surgery, chemotherapy ± radiotherapy)
- ECOG performance status 0 to 2
- Satisfactory haematological or biochemical functions
  (Tests should be carried out within 8 weeks prior to randomisation:- ANC ≥ 1.0 x 109/L, Platelets ≥ 100 x 109/L, Creatinine clearance ≥ 30 mL/min, Total bilirubin ≤ 2 x the upper limit normal, AST & ALT ≤ 5 x the upper limit normal)

- Completed the following investigations
  - Colonoscopy(or CT colonogram(within 16 months prior to randomization)
  - Imaging of abdomen (CT or CT colonogram or MRI or PET or Ultrasound) within 16 months prior to randomization
- Written informed consent

Exclusion Criteria

- Pre-existing Familial adenomatous polyposis, inflammatory bowel disease or ulcerative colitis
- Active gastritis or active peptic ulcer
- History of continuous daily use of PPI more than 1 year prior to consent
- Gastrointestinal bleeding within the past one year
- Haemorrhagic diathesis (i.e. haemophilia)
- Uncontrolled hypertension (untreated systolic blood pressure > 160 mmHg, or diastolic blood pressure > 95 mmHg)
- History of recent cancers (except for colorectal cancers, non-melanoma skin cancers, basal cell carcinomas, squamous cell carcinomas) in the past 5 years
- History of stroke, coronary arterial disease, angina, or vascular disease
- Patients who are on current long term treatment (≥ 4 consecutive weeks) with Aspirin, NSAID or Cox-2 inhibitors
- History of erosive GERD or active erosive GERD on gastroscopy.
- Patient on active current treatment with antiplatelet agents (i.e. off-study Aspirin, clopidogrel, ticlopidine)
- Patient receiving current treatment with anticoagulants (i.e. warfarin, low molecular weight heparins)
- Pregnant, lactating, or not using adequate contraception
- Patient having known allergy to NSAID or Aspirin
- Unexplained rise of CEA (i.e. smoker with elevated CEA will not be excluded)
- Patient on other investigational drug
- Patients with HNPCC (Lynch Syndrome)

Treatment Groups and Randomisation

Eligible patients will be randomized to two treatment arms, (either 200mg Aspirin for 3 years or 200mg Placebo for 3 years) using the following stratification factors:

- Study centre
- Tumour type (Dukes C colon, high risk Dukes B colon cancer & rectal cancer sub-groups) and
- Type of adjuvant chemotherapy received (exposed/ not exposed to oxaliplatin)

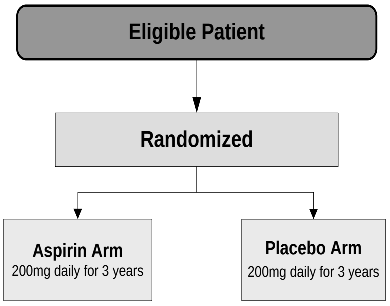

Study Duration

Patients will be receiving aspirin or placebo for 3 years after randomisation and followed up for 5 years after randomization. The study is expected to recruit the last patient by end of 2020, with the last patient potentially being followed up until 2025.
NOTE– (these dates may need changing according to new estimated recruitment timelines)

Follow-Up

After randomization, patient will have 3 monthly assessments for 3 years (month 3 t*o month 36) followed by 6 monthly assessments for additional 2 years (month 42 to month 60).* Subjects who consent to continue information collection after month 60 will have vital data captured via review of medical records or phone call. Data capture for vital status, recurrence, NSAIDs consumption and medical events (e.g. stroke, acute mycocardial infarction [AMI], new cancers, and major bleeds) will occur every 12 months and up to the time of last patient last visit (month 60 or earlier) at the site.

Statistics and Analysis Plan

Based on recent studies of aspirin in colorectal cancer patients, we assumed a hazard ratio of 0.72 for disease free survival between the aspirin and the placebo groups (the corresponding absolute difference of disease free survival rate between the two treatment groups is about 8.5%, assuming that 3-year disease free survival rates for Dukes B colon cancer, Dukes C colon cancer and rectal cancer are 65% after standard adjuvant chemotherapy). A total of 300 recurrences (or deaths) is required for a two-sided log-rank test of 5% type I error, 80% power and 15% attrition rate. This translates into a total sample size of 1200 patients if the disease free survival rate is 65% in the placebo group.

A recent review of trial interim data, after enrollment of at least 1140 patients and 3 year follow-up being completed in 430 patients, revealed a pooled disease free survival rate of 79% at 5 years, we subsequently recalculated the sample size to be 1587 patients (attrition rate reduced to 10%, the rest unchanged).

In analyzing the Primary Endpoint: Disease Free Survival (DFS) and Secondary Endpoint: Overall Survival (OS), logrank test will be used to compare between groups. Hazard ratio and its 95% confidence interval will be estimated. Cox model will be further performed to adjust for potential confounding factors.

Two interim analyses are scheduled. The first analysis is a safety and toxicity analysis planned to be done after 540 patients are recruited into the study or at the mid-point of the targeted recruitment period, whichever comes earlier. The second analysis will look at safety as well as DFS and OS, after 540 patients have been followed up for 3 years, or when the cohort of patients included in the first analysis have completed follow up for 3 years..

Assessments	Screening*		Baseline	Follow-up month (+/- 1 month)			Follow-up month (+/- 1 month) After Recurrence	Follow-up month (+/- 1 month) After Early Study Drug Discontinuation and no recurrence	Post Month 60
				3, 9 ,15, 21, 27, 33	6,12, 18, 24, 30, 36	42, 48, 54, 60	3 to 60	3 to 36	
Written informed consent		x							
Demography, Tumour history, Medical history & family history			x						
Record surgical measurements,(For rectal cancer only)			x						
Adjuvant Therapy			x						
Vital signs (Body weight, BP, height), ECOG			*x*						
Hb	(Within 8 weeks prior to randomisation)		x		x				
Platelet count, Creatinine, Serum Bilirubin, AST, ALT	(Within 8 weeks prior to randomisation)	x							
CEA			x	x	x	x		x	
Imaging of abdomen (CT or CT colonogram or MRI or PET or Ultrasound)	To be done if not done within 16 months prior to randomization	x		x (Mth 6, 18 and 30) Optional; or frequency according to institutional practice					
Radiological examination of the Chest (including but not restricted to Chest x ray, CT thorax or PET scan)	To be done if not done within 16 months prior to randomization	x							
Colonoscopy/CT colonogram	Full colonoscopy if not done within 16 months prior to randomization	x		x (Mth 6 and 30) Optional; or frequency according to institutional practice					
AE and SAE				x	x				
Death				x	x	x	x	x	x
Medical Events				x	x	x	x	x	x
Concomitant NSAIDs				x	x	x	x	x	x
Concomitant treatment/medication				x	x				
Recurrence, Salvage Chemotherapy				x	x	x	x	x	x

Notes:
*Screening can start after last dose of standard therapy;

1. INTRODUCTION

1.1. Background

Colorectal cancer is the third most common cancer worldwide with almost 1 million new cases diagnosed each year. It is now also the third leading cause of cancer mortality in men and women with more than half of diagnosed patients dying from the disease.[1] Over the past 3 decades, the age-standardized incidence rate for colorectal cancer has increased from two to fourfold in Asian countries such as China, Japan, South Korea and Singapore. Mortality rates in Asian countries have risen concomitantly and in Singapore rates have doubled over the same period, .[2] now with incidence rates amongst the highest in Asia; colon cancer has recently surpassed lung cancer as the commonest cancer diagnosed in Singapore. [3]

With the rising rates of cancer, a fundamental shift of the cancer burden has also occurred between the developed and underdeveloped world. This problem, highlighted in the 2008 WHO World Cancer Report, warned of a disproportionate number of cancer deaths occurring in developing countries. Currently of the annual 12 million new cancer cases diagnosed and the 7.6 million cancer deaths worldwide; 5.6 million new cases and 4.7 million cancer deaths occurred in developing countries.[4] By the year 2020, it is predicted that changes in the demographics of the population in developing nations will lead to approximately 70% of all new cancers occurring in lower income countries.[5] Efforts therefore will have to be made to develop novel therapies that are not only effective but also accessible to the people who need them.[6]

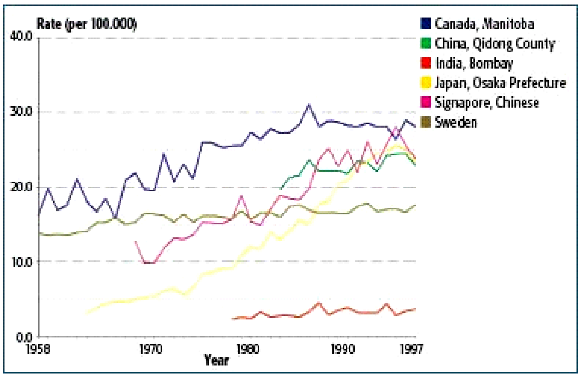

Fig. 1 Time trends of ASR of colorectal cancer of males from Western and Asian cancer registries showing increasing rates. WHO/IARC database 2000.

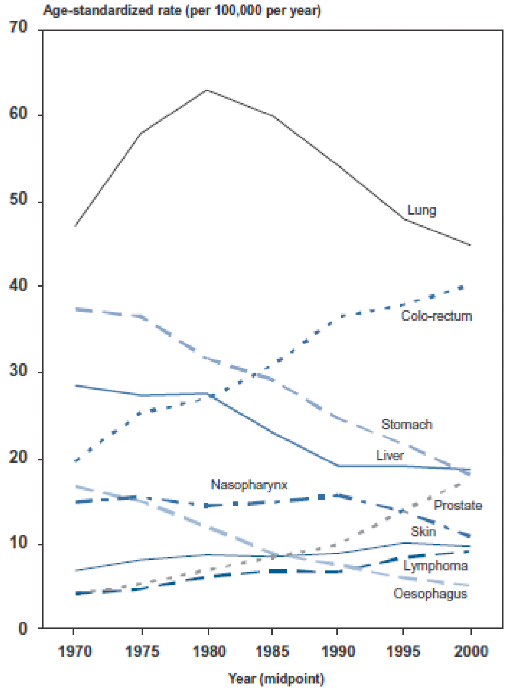

Fig 2. Trends in Age standardised Incidence for selected cancer sites in Singapore Males (1968-2002)

1.2. Adjuvant agents for colorectal cancer

The 5 FU Era

Adjuvant chemotherapy has been proven to improve relative overall survival in duke's C colon cancer by approximately 25-35%, in a series of landmark clinical trials conducted over the past 3 decades (table 1). The first trial to show benefit was NSABP-C01 conducted in the 1980s. Post-surgical patients randomised to Semustine, Vincristine and 5FU (MOF) chemotherapy were found to be 30% less likely to develop recurrences or death compared with those receiving surgery alone.[7]

Two subsequent studies further established 5FU based chemotherapy as the standard of care. In the study by Moertel et al, post-operative intravenous 5FU combined with levamisole reduced the relative risk of recurrence and death by 40% and 33%, and the absolute risk of death by 12%, compared with standard surgery alone.[8] For the North Central Cancer Treatment Group (NCCTG) study, colon cancer relapse and death was significantly lower in patients receiving intravenous 5FU/leucovorin compared with surgery alone - 27% versus 41% and 28% versus 40% respectively.[9]

Over the next two decades, due to a lack of effective new agents, development continued to focus on 5FU based regimens comparing duration, schedules, and administration route. For example, 12 month was compared with 6 month duration of chemotherapy and continuous infusion 5FU (via pump) was compared with bolus 5FU in 3 large randomised studies. Although toxicity was lower for infusional 5FU, overall survival was not improved.[10-12] Finally, in 2005, the landmark X-ACT study, demonstrated oral 5FU (capecitabine) to be non-inferior to IV 5FU. (Twelves et al).[13]

Trial Name	No. of Subjects	Intervention Arms	Results	Reference
NSABP C03	1166	MOF versus BCG versus Observation	Improved DFS and OS	J Natl Cancer Inst 1988;80(1):30-6.
Intergroup	929	5FU/ levamisole vs levamisole alone	Improved DFS and OS	Ann Intern Med 1995;122(5):321-6
NCCTG	317	5FU/Leucovorin vs Observation	Improved DFS and OS	J Clin Oncol 1997 Jan;15(1):246-50
X-ACT	1987	Capecitabine (oral 5FU) vs Bolus 5FU (mayo regimen)	No difference between IV and oral 5FU	N Engl J Med 2005;352:2696-704

Table 1. Clinical Trials - Adjuvant chemotherapy for colon cancer (1988 – 2005)

Oxaliplatin and Irinotecan

A year before X-ACT, the MOSAIC study was able to establish a new standard of care. For the first time in two decades, a new agent (oxaliplatin), was shown to improve 3 year disease free survival (DFS) over infusional 5FU alone.[14] After 7 years of follow up, oxaliplatin was associated an absolute 2.5% survival gain for Dukes C colon cancer. Although Oxaliplatin-5FU combinations have become the new standard of care for Dukes C colon cancer; neurotoxicity and thrombocytopenia continue to remain significant challenges in the clinic, and the higher cost of oxaliplatin still represents a substantial barrier to its routine adoption in many countries including Singapore.

Since the MOSAIC study was published in 2004, no other new agents have been shown to improve colon cancer outcomes (Table 2). For example, although highly effective in the metastatic setting,

Irinotecan chemotherapy has failed in three large randomised adjuvant studies – the EORTC PETACC-3, ACCORD and CALGB 89803.[15-17] The failure of irinotecan is particularly disappointing, since there are so few agents with a similar track record of efficacy in the metastatic setting. However, the consistent negative results in these high profile studies, means that further trials with Irinotecan will unlikely be undertaken in the future.[18] With conventional chemotherapy combinations appearing to be approaching their therapeutic index and limit, much hope is now centred on combinations using biologic agents, for example bevacizumab and cetuximab.

Trial Name	No of Subjects	Intervention Arms	Results	Reference
**Oxaliplatin**				
MOSAIC	1123	Infusional 5FU / Oxaliplatin<br>Vs<br>Infusional 5FU alone	Improved DFS	N Engl J Med 2004;350:2343-51
**Irinotecan**				
PETAAC 3	2124	Irinotecan/5FU<br>Vs<br>5FU	No difference	J Clinl Oncol, 2005;23, (16S): 8
ACCORD	2014	Irinotecan/5FU<br>Vs<br>5FU	No difference	J Clin Oncol. 2005;23(16S):3502.
CALGB 89803	1264	Irinotecan/5FU<br>Vs<br>5FU	No difference	J Clin Oncol. 2004;22(14S):3500.

Table 2. Clinical Trials - Oxaliplatin and Irinotecan Chemotherapy

1.3. Failure of targeted Agents

Bevacizumab The idea of using anti-VEGF monoclonal antibodies such as Bevacizumab, upfront in the adjuvant setting is one that carries particular scientific merit. The concept of the "angiogenic switch" means that tumours more than 1mm would need to eventually grow their own blood supply in order to survive.[19] Consequently , bevacizumab should be theoretically even more effective, when used to treat tumours that are small, and particularly vulnerable to vascular disruption. Two large adjuvant bevacizumab studies have been undertaken to prove this concept (table 3) – the NSABP C08 trial and the AVANT study.[20] The results of the C08 study was presented at ASCO 2009 and was negative for its primary endpoint[21]. 2632 patients were randomised to either 5FU/Oxaliplatin or the same chemotherapy with Bevacizumab and after a median follow up of 3 years, there was no significant difference in disease free survival between the 2 populations. The failure of bevacizumab in the adjuvant setting has forced a serious re-evaluation of our current understanding of tumour dormancy and treatment of micrometastasis disease free survival.

Trial Name	No. of Subjects	Intervention Arms	Results	Reference/ Comments
NSABP-C08	2632	Oxaliplatin/5FU/<br>Bevacizumab<br>Vs<br>Oxaliplatin/5FU alone	Negative	J Clin Oncol 27:18s, 2009 (supple; abstr LBA4)
AVANT	3450	Oxaliplatin/5FU/<br>Bevacizumab<br>Vs<br>Oxaliplatin/Capecitabine/<br>Bevacizumbab<br>Vs<br>Oxaliplatin/5FU alone	Pending	www.clinicaltrials.gov: NCT00112918

Table 3. Adjuvant Bevacizumab studies

Cetuximab, a chimeric human-mouse monoclonal antibody to VEGF receptor is currently under development as an adjuvant agent for colon and rectal cancers (table 4). [22-23] Although Cetuximab is highly effective in the metastatic setting, the preliminary results of the NCCTG Intergroup 0147 study evaluating its use in the adjuvant setting indicates that it improves neither disease free survival nor overall survival.[24]

Trial Name	No of. Subjects	Intervention Arms	Results	Reference/ Comments
NCCTG Trial	2600	Chemotherapy with or without 5FU	Negative	www.clinicaltrials.gov NCT00079274
French Study (FFCD)	2000	Ox/5FU/Cetuximab Vs Ox/5FU	-	www.clinicaltrials.gov NCT00265811

Table 4. Adjuvant Cetuximab studies

1.4. NSAIDS as adjuvant agents

Cox 2 specific inhibitors. Since the discovery that cox-2 enzyme was consistently over-expressed in colon cancer; a large body of pre-clinical scientific evidence has emerged implicating cox and PGE2 in cancer initiation and propagation.[25-26] This has culminated in two large randomised trials evaluating Cox-2 specific inhibitors as specific adjuvant agents in colon cancer (table 5) – VICTOR and the EORTC PETAAC 5. Both these trials have however been discontinued following concerns about the cardiovascular safety for these agents when in extended use. PETAAC 5 was a double blind randomised placebo controlled study, and evaluated celecoxib or placebo for 3 years and used DFS as a primary endpoint.[27] Unlike ASCOLT, celecoxib was administered concurrently with chemotherapy (this is not possible with aspirin due to the risk of chemotherapy induced thrombocytopenia) and the population under study was restricted to Duke C colon cancer patients only.

Launched in 2001, VICTOR randomised patients with stage II or III colon and rectal cancer that had completed standard adjuvant therapy to 2 years of rofecoxib, 5 years of rofecoxib or placebo, before it was prematurely suspended in 2005 when rofecoxib was withdrawn from the market.[28] In contrast to the EORTC study, VICTOR only randomized patients after completion of standard adjuvant therapy and it also included rectal cancers and stage II disease. In the analysis of 2300 patients who had been treated with study drug for a median of 7 months, there was no difference observed between the two treatment groups in terms of disease free survival and overall survival.[29] However due to the short median exposure to study medication (7mths versus the planned 2 and 5 years), and the failure to achieve accrual target, it is not possible to make any conclusion concerning efficacy. Of note however, survival during the first year was significantly superior in favour of rofecoxib, during the first year of study (when patients were exposed to study drug), but the difference was insignificant at 5 years.

Study Name	No of Subjects	Intervention	Results	Reference
EORTC PETAAC5	1450	Celecoxib vs Placebo	Suspended	www.clinicaltrials.gov NCT00085163
VICTOR	7000	Rofecoxib vs Placebo	Suspended	Annals of Oncology 19 (Supplement 8):2008, LBA3

Table 5: Adjuvant Cox-2 Inhibitor studies

Aspirin. The first suggestion that NSAIDs/ Aspirin may be beneficial in the adjuvant setting derives from a pre-planned review of patients in the CALGB 89803 study. This study randomised stage III colon cancer patients to 5FU chemotherapy with or without irinotecan, and in addition to the primary study, incorporated a pre-planned analysis to compare outcomes of patients who were on Aspirin or cox2 inhibitors. Of the 830 patients surveyed, 75 patients and 41 patients used Aspirin and Cox-2 inhibitors regularly. Amongst Aspirin and Cox2 users, the hazard ratio for disease recurrence was 0.45 (95% CI 0.21-0.97) and recurrence and/or death 0.48 (95%CI 0.24-0.99). No difference however was noted with paracetamol use.[30]

A nest cohort study (Nurses' Health Study) suggested that the initiation of Aspirin *after* the diagnosis of colon cancer was able to reduce colorectal cancer specific mortality (HR 0.53, CI 0.33-0.86) on multivariate analysis[31]. Regular aspirin use after diagnosis was associated with an impressive lowering of colorectal cancer−specific mortality among participants in whom primary tumours overexpressed COX-2 (multivariate HR, 0.39; 95% CI, 0.20-0.76). This is in contrast to patients with tumours that had weak or absent cox2 expression where aspirin use was not associated with lower risk (multivariate HR, 1.22; 95% CI, 0.36-4.18). This suggests a biologically plausible mechanism for aspirin's activity.

The California Teachers Study conducted by Zell *et al* was a prospective cohort study comprising 133,479 female public school teachers and administrators[32]. Aspirin and ibuprofen use was assessed by a self-administered questionnaire, and incident diagnosis of CRC was identified through the California Cancer Registry database. Amongst 621 participants who developed CRC, regular NSAID (aspirin or ibuprofen) use pre-diagnosis was found to decrease overall risk of death and death from CRC. Amongst regular pre-diagnosis aspirin or NSAID users (approximately a third of the study cohort), the multivariate HR for CRC-specific mortality was 0.62 (95% CI 0.41–0.94), and for overall survival was 0.74 (95% CI 0.54–1.01). When analysed for duration of use, 5 or more years of aspirin use was associated with an even greater benefit for CRC-specific survival (RR = 0.33; 95% CI 0.18–0.63) and for overall survival (HR = 0.53; 95% CI 0.34–0.83).

More recently, Bastiaannet *et al* published the largest observational study evaluating the benefit of post--diagnosis 'adjuvant' aspirin[33]. In total, 4,481 patients with CRC were identified from a population cancer registry in the Netherlands, and aspirin use was assessed from a centralized prescription database. Frequent aspirin use post CRC diagnosis was associated with significant survival gain (adjusted HR 0.70; 95%CI 0.57–0.88). Aspirin dose in this Dutch study was 80mg daily.

Most recently McCowen et al analysed 2990 CRC patients in a Scottish cohort[34]. Aspirin use post-diagnosis was associated with lower risk of all-cause mortality (HR =0.67, 95%CI 0.57–0.79) and colorectal cancer-specific mortality (HR 0.58; 95%CI 0.45–0.75). Majority of patients in this study used low dose Aspirin (75mg daily).

Although the results from these studies are extremely exciting and have the potential to open up new avenues in our understanding of cancer biology; because they are observational, the results will have to be proven in a prospective randomized placebo-controlled study. The ASCOLT study is the first study to evaluate this.

Study Name	No of subjects	Study Type	Intervention	Results	Reference
CALGB 89803 (Fuchs et al)	830	Nested case-control study	5FU/Leu +/- Irinotecan<br><br>Patients prospectively surveyed for Aspirin /Cox2 use	Lower recurrence for patients on Aspirin and cox2 inhibitors (RR 0.48). No benefit for paracetamol	J Clin Oncol 2005;23S: 3530
Nurses Health Study (Chan et al)	1279	Nested case-control study	Analysis of outcomes with regards to aspirin use	Reduced colorectal cancer specific mortality (HR 0.53)	JAMA 2009;302(6):649-659
California Teachers Study (Zell et al)	621	Nested case-control study	Analysis of outcomes with regards to pre-diagnosis aspirin use	Reduced CRC-specific mortality (HR 0.62)	Cancer 2009; 115: 5662–5671
Dutch Study (Bastiaannet et al)	4481	Cancer Registry Study	Analyzed according to Post-CRC diagnosis aspirin use	Reduced overall mortality (HR 0.70)	Br. J. Cancer 2012; 106: 1564–1570.
Scottish Study (McCowen et al)	2990	Cancer Registry Study	Analyzed according to Post-CRC diagnosis aspirin use	Reduced CRC-specific mortality (HR 0.58)	Eur J Cancer 2012. http://dx.doi.org/10.1016/j.ejca.2012.10.024

Table 6. Evidence supporting role for Aspirin in Established Colon Cancer

1.5. Aspirin's Secondary Role – Prevention of new polyps

There is currently high quality evidence from a series of prospective placebo-controlled randomized studies that Aspirin and NSAIDs reduced the incidence of colorectal adenomas (table 7). Therefore since patients with resected colorectal cancer are at increased risk of developing a second metachronous cancer (up to 3% of patients in the first 5 years), they may derive secondary benefit from the use of aspirin.

In the study by Sandler et al, a total of 635 patients were randomised to receive Aspirin 325 mg daily or placebo. One or more adenomas were found in 17% of patients in the Aspirin group and 27 % of patients in the placebo group (P=0.004). The relative risk of any polyp in the Aspirin group was 0.65 (95%CI 0.46-0.91) and the time to detect the first adenoma was longer in the Aspirin group than in the placebo group.[35] In the Baron study, patients were randomised to receive Aspirin at 81 mg, 325 mg or placebo. For advanced neoplasm (adenomas measuring at least 1 cm, or with villous, tubulovillous features, severe dysplasia or invasive cancer) the relative risks were 0.59 (95% CI 0.38-0.92) for the 81 mg arm and 0.83 for the 325 mg Aspirin arm.[36] In a third trial by Logan et al, 945 patients were randomised to Aspirin, Folate supplementation or placebo. Whereas no benefit was found for folate, Patients on Aspirin had a significant reduction in polyp burden.[37] More recently, a 375 patient randomised study demonstrated that patients treated with Sulindac and Difluoromethylornithine (DMFO), a drug also used to treat trypanosomiasis, reduced the risk of recurrent adenomas by 72% and high risk adenomas by 92%.[38] This magnitude of reduction was much larger than expected and in fact was the greatest seen for any chemoprevention trial to date and this has sparked off a renewed interest in the use of NSAID combination agents for chemoprevention.[39] Cox2 inhibitors studies (i.e. APC, PreSAP, APPROVe) have also shown this class of agents to be effective in reducing polyps,[40-42] however their adverse cardiovascular profile precludes their long term use for this indication. Aspirin however is cardio-protective and can be safely administered over prolonged periods of time.[43-44]

Study / Author	No of Subjects	Intervention	Results	Reference
Baron et al	1121	Aspirin 81mg vs Aspirin 325mg vs Placebo	RR 0.59 for high risk polyps in 81mg Aspirin arm	N Engl J Med 2003;348:891
Sandler et al	517	Aspirin vs Placebo	RR 0.65 for any adenoma in 325mg Aspirin arm.	N Engl J Med 2003;348:883
Logan et al	945	Aspirin 300mg Vs Folate 0.5mg Vs Placebo	RR 0.79 for any adenoma and 0.63 for high risk adenoma in Aspirin arm	Gastroenterology 2008; 134:29
Meyskens et al	375	Sulindac + DMFO Vs Placebo	RR 0.3 for any adenoma and RR 0.085 for high risk adenoma	Cancer Prev Res 2008;1:32
APC study	1364	Celecoxib vs Placebo	RR 0.67 for 200mg bd dose and RR 0.55 for 400mg bd dose	N Engl J Med. 2006;355:873
PreSAP study	1561	Celecoxib vs Placebo	RR 0.64 for adenomas and RR 0.59 for high risk adenomas	N Engl J Med. 2006;355:885
APPROVe Study	2587	Rofecoxib vs Placebo	RR 0.76 for adenomas. RR 0.65 for advanced adenomas	Gastroenterology 2006;131:1674

Table 7. NSAIDs and Polyp prevention

1.6. Primary Prevention of Colorectal Cancer

A large number of epidemiological and clinical studies have shown a strikingly consistent 40 to 50 percent reduction in the risk of colorectal neoplasia, in patients on regular aspirin, despite the differences in study designs, populations, and patterns of Aspirin use.[45]

In a retrospective Australian case control study performed in the 1980s, Kune et al noticed that there was a significant deficit of heart disease, stroke and arthritis in patients who had colon cancer who used regular aspirin amongst 715 colon cancer cases matched against 727 controls. On multivariate analysis, controlling for heart disease and stroke, the association between aspirin use and incidence of colon cancer persisted and remained highly significant across gender and disease site.[46]

Later, in a prospective mortality study of 662,424 individuals who provided information on the frequency and duration of their aspirin use, Thun et al noted that death rates from colon cancer decrease with more frequent aspirin use. The relative risk among persons who used aspirin 16 or more times per month for at least one year was 0.60 in men (95% CI 0.40 - 0.89) and 0.58 in women (95% confidence interval, 0.37 - 0.90). Adjustment for dietary factors, obesity, physical activity, and family history did not alter the findings significantly and no association was found between the use of acetaminophen and the risk of colon cancer.[47]

Further support comes from the analysis of the results of the National Health and Nutrition Examination Survey I (NHANES I) and the NHANES I Epidemiologic Follow-up Studies (NHEFS). Amongst 12 668 responding subjects who were followed up an average of 12.4 years, 1,257 were eventually diagnosed with cancer. The relative risk of colorectal cancer amongst younger men who were regular users of aspirin was 0.35 (95% CI 0.17-0.73).[48]

In the Nurses Health Study, women were surveyed using 3 consecutive questionnaires in 1980, 1982 and 1984. Incidence of colorectal cancer in women who were consistent users of aspirin, defined as

2 or more tablets a week, were compared with non-users. In total, 331 new cases of colorectal cancer diagnosed during 551,651 person-years of follow-up. Although there was no difference at 4 years; after 20 years of consistent aspirin use, there was a statistically significant reduction (relative risk, 0.56; 95% CI 0.36 to 0.90; P for trend = 0.008).[49].

More recently, a combined analysis of two randomised aspirin trials (The British Doctors Aspirin Trial and the UK-TIA Aspirin Trial), demonstrated that Aspirin reduced the incidence of colorectal cancer with a Odds Ratio (OR) of 0.74 (95% CI 0.56-.97). Subjects in these studies were randomised prospectively to aspirin or placebo, but with a vascular outcome as the primary endpoint. In compliant patients who received Aspirin for 5 years or more, the OR for incidence of new colorectal cancer at 10-14 years was 0.26 (95% CI 0.12-0.56, p=0.0002). A systemic review of published observational studies performed by the same authors showed that in 19 case-control studies (20,815 cases) and 11 cohort studies (1,136,110 individuals), regular use of Aspirin or NSAID was consistently associated with a reduced risk of colorectal cancer.[50]

In the Women's Health Study, 39 876 women were randomised in a 2x2 factorial trial design to aspirin 100mg every other day, vitamin E and placebo. After an average 10 yrs of follow up, 2865 new cases of invasive cancers were diagnosed however there was no effect of Aspirin on the incidence of all cancers or colorectal cancer. This study was however limited by the low dose and (alternate-day) schedule of aspirin administration.[51]

**Study / Author**	**Study Type**	**No of Subjects**	**Results**	**Reference**
Kune et al.	Case-Control	715 colon cases vs 727 controls	significant deficit of colon cancer amongst aspirin users	Cancer Res 1988 ;48:4399
Thun et al.	Nested Case-Control	662,424	Death rate from gastrointestinal cancer 40% lower amongst individuals who used Aspirin 16 times/ mth or more.	Cancer Res 1993; 15; 53 (6): 1322-7
NHANES I and NHEFS study	Nested Case-Control	12,668	Lower incidence risk across several cancers with the lowest risk amongst young male aspirin users	Epidemiology 1994; 5(2):138-46
Nurses Health Study	Nested Case-Control	551,651 person yrs of follow up	Lower risk of colon cancer with prolonged aspirin use	N Engl J Med 1995; 333 : 609-14
US Physician Health Study	Randomised trial evaluating aspirin in vascular disease	22,071	No Benefit	N Engl J Med 1989; 321:129
Women's Health study	Randomised trial evaluating aspirin in vascular disease	39,876	No Benefit	JAMA 2005;294:47
Flossman et al	Randomised trials evaluating aspirin in vascular disease	7588	Overall lower incidence of colorectal cancer HR 0.74, (95% CI 0.56-0.97) in Aspirin users.<br><br>Amongst compliant patients with prolonged (>5 yrs) use, HR 0.26 (95CI 0.12-0.56)	Lancet. 2007 May 12;369(9573):1603-13

Table 8. Aspirin and Primary prevention of colorectal cancer

1.7. Preclinical data and scientific rationale

The pathways and mechanisms in which aspirin/ NSAIDs inhibit colon cancer cell growth are multiple, and complex.[52,53] Aspirin and Indomethacin have been shown in numerous animal studies to reduce the incidence, multiplicity and size of chemically induced gastrointestinal cancers[54-56] and in APC min mice.[57] Aspirin has also been shown to have anti-angiogenic properties in vitro and is able to potently inhibit endothelial cell migration and endothelial capillary tube formation.[58] Sulindac and cox2 inhibitors have also been shown to reduce microvascular density, cell proliferation index and increase apopotosis in-vivo, in athymic xenograft mouse models.[59] Recently in an aggressive ovarian clear-cell cancer mouse model, mice treated with aspirin were noted to have markedly lower ascitic volume, smaller area of diaphragmatic tumor coverage, and a longer time to disease progression and distant metastatasis.[60] Through a series of experiments, the authors were able to show that increased local and systemic inflammation always accompanied tumor progression and the extent of inflammation also corresponded with disease progression. By modulating inflammation via macrophage depletion, researchers were able to alter rate of cancer progression in the mice – thus demonstrating that aspirin's activity was dependent on the adaptive immune system. This is particularly interesting because inflammation stands at the nexus of the adaptive immune response, and the latter is an extremely powerful prognostic marker in human colon cancer.[61]

Whilst it is well known that chronic inflammation predisposes to certain malignancies, it has only recently recognized that a maladaptive inflammatory response is required for malignant transformation and metastasis.[62] Indeed Hanahan and Weinberg have included tumor promoting inflammation as one of the key hallmarks of cancer. [63] Inflammation has been shown to induce widespread epigenetic changes in ileum of mice (i.e. methylation of polycomb target genes) leading to silencing of multiple genes required for the development of cancer.[64] Studies have also linked proinflammatory PGE2 (a downstream product of cox activation) with colon cancer carcinogenesis via complex interactions with stem cell pathways.[65,66] Indeed mice which lack the ability to produce PGE2 have resistance to colon cancer.[67] The evidence from preclinical studies is also finding some support from several human studies. For example, a very large cohort study in China found a strong association between urine PGE2 and the risk of developing colon cancer[68] and another Scottish study, noted that high pre-operative CRP levels correlated with poor tumor specific survival.[69] Low doses of Aspirin has been shown to be highly effective in reduce rectal mucosal PGE2 levels in human subjects[70,71] and this may be one reason to explain its anti-cancer effect.

Aspirin is a non-selective inhibitor of cox and binds to both cox-1 and cox-2 in an irreversible fashion. Whereas cox-1 is constitutively activated in all tissues, cox-2 represents an inducible isoform that is only expressed in inflamed tissues. Cox-2 is not normally expressed in normal colonic epithelial tissue, however it is over-expressed in 40-50% of adenomatous polyps and in 85% of colon cancers.[72] Further, the intensity of cox over-expression is higher in metastasis when compared to the primary colonic tumor. Cox-2 has been shown to play an important role in tumor growth and studies of cox-2 knock-out mice have demonstrated inhibition of tumor growth versus wild type mice. Conversely, when wild type mice were treated with cox-2 inhibitors, it also demonstrated a similar ability to retard tumor growth.[73] It is currently believed that NSAIDs and cox-2 inhibitors also act on additional pathways apart from cox. Invitro studies in some instances seem to indicate NSAIDs do not require

the presence of cox-2 to prevent cancer and are able to demonstrate an ability to inhibit cell proliferation, angiogenesis and to induce apoptosis, even in the absence of cox-1 and/or cox-2.[74]. Indeed, most recent analysis of colorectal cancer patients in the Nurses Health Study indicate that tumor PIK3CA mutational status may help select for patient who would benefit from aspirin treatment.[75]

1.8. Optimal biologically effective dose for Aspirin

The optimal dose of Aspirin as an adjuvant agent for colorectal cancer is not known. There have been, to date, no randomised Aspirin trials exploring *secondary prevention* as an endpoint. Non-randomised studies evaluating Aspirin as a *primary prevention* agent have suggested that a dose of 300 mg a day for 5 years is effective [31,50]. In the analysis of the Nurses Health Study, regular use of Aspirin (325 mg) twice or more per week was shown to reduce the incidence of colorectal cancers that over express Cox-2 but not the incidence of cancers with weak or absent expression of Cox-2.

In the *polyp prevention* study by Baron J A et al, a lower dose (81 mg) of Aspirin appeared to be at least equally as effective as an intermediate dose (325 mg) in preventing recurrence of polyps[36]. In addition, dose escalation studies in normal human subjects using *mucosal PGE2 as a biomarker* have suggested that 81 mg Aspirin dose was sufficient to significantly suppress rectal mucosal PGE2 levels and did so to an equivalent extent as higher doses[70,71] .

In both the CALGB 89803 analysis[30], and the study by Chan et al,[31] the majority of participants were taking Aspirin at 325 mg per day (personal communication, Charles Fuchs and Andrew Chan). In contrast, subjects in the studies by Bastiaannet et al and McCowen et al utilized low-dose aspirin (75mg to 80mg).[32,33]

Our study uses a 200 mg daily dose, which falls directly in the middle of the dose range used by patients in the various cohort studies. We believe this intermediate dose will most optimally define the trade-off between efficacy and tolerability.

2. STUDY OBJECTIVE

To assess the effectiveness of Aspirin against placebo control in patients with dukes C or high risk dukes B colorectal cancer in terms of Disease Free Survival (DFS) and Overall Survival (OS)

3. STUDY ENDPOINTS

Primary endpoints

- DFS among all eligible subjects (high risk Dukes B colon cancer, Dukes C colon cancer and rectal cancer patient sub-groups);
- DFS among patients with colon cancer (high-risk Dukes B and Dukes C colon cancer).

Secondary endpoints

- Overall survival (OS) over 5 years
- DFS and OS in
    - Chinese, Malay, Indian and other ethnic groups
    - Resected high risk Dukes B colon cancer, Dukes C colon cancer and rectal cancer sub-groups, individually
    - Compliant versus non-compliant subjects
    - PIK3CA mutated tumors (where samples are available)

Endpoint definitions

Disease recurrence is defined as any one of the followings:

1. Unequivocal radiological evidence of colorectal cancer recurrence
2. Recurrence detected by Digital rectal examination (DRE)
3. Positive histology or cytology (i.e. peritoneal or pleural cytology)
4. Colonoscopic evidence of local cancer recurrence at the previous operation site
5. Detection of a new colon or rectal primary tumour

Disease free survival is defined as the time from randomisation to the time of documentation of disease recurrence or death from any cause. If there is no disease recurrence or death by the time when a patient is last followed up, it will be censored at that time.

Overall survival is defined as the time from randomisation to the time of death from any cause. If there is no death by the time when a patient is last known to be alive, it will be censored at that time.

Compliant subject is defined as a subject who takes the study drug for more than 70% of days for the study period during which the patient is on treatment, up to the time of discontinuation.

4. TRIAL DESIGN

Dukes C colon cancer, high risk Dukes B colon cancer, Dukes B rectal cancer or Dukes C rectal cancer patients who have completed the resection of primary tumour will be treated with standard

therapy (chemotherapy ± radiotherapy). Adjuvant chemotherapy is not specified but should consist of at least 3 months of a 5FU based chemotherapy – for example, weekly 5FU/FA (Roswell Park regimen), monthly 5FU/FA (Mayo Clinic regimen), infusion of 5FU, oral capecitabine, FOLFOX or Capecitabine/Oxaliplatin regimens. Rectal cancers may additionally have adjuvant radiotherapy administered in either adjuvant or neoadjuvant fashion. During the last cycle of standard therapy, prospective patients would be invited to participate in this study. Once informed consent is taken & within 120 days of completion of the standard therapy, they would be checked for trial eligibility.

An eligible subject will be randomised to the study in 1:1 ratio to either

- Aspirin arm: 200 mg Aspirin once a day for 3 years
- Placebo arm: 200 mg matching placebo once a day for 3 years.

After randomisation, patient will have 3 monthly assessments for 3 years (month 3 to month 36) followed by 6 monthly assessments for additional 2 years (month 42 to month 60). All subjects will be approached to seek consent for survival information (DFS, OS) after 5 years post randomization will have data collected via review of medical records or by phone contact, every 12 months until, and at the last patient last visit (month 60 visit or earlier) at the site.

The purpose of 5 year follow up is to evaluate-

1. The long term survival outcomes and
2. Persistence of effect after completion of treatment.

5. ELIGIBILITY CRITERIA

5.1. Inclusion Criteria

- Male or female outpatient of ⩾ 18 years of age or ⩾ country's legal age for adult consent
- Dukes C colon cancer, high risk Dukes B colon cancer, Dukes B rectal cancer or Dukes C rectal cancer (*see Appendix 1 for definition of High Risk Dukes B)*
- Undergone complete resection of primary tumour
- Completed standard therapy (at least 3 months of chemotherapy ± radiotherapy)
- Within 120 days of completion of standard therapy (surgery, chemotherapy ± radiotherapy)
- ECOG performance status 0 to 2
- Satisfactory haematological or biochemical functions (tests should be carried out within 8 weeks prior to randomisation): Results of clinical investigations carried out within 8 weeks prior to randomisation can be used in place of the required screening investigations. Patients with mild laboratory abnormalities can be included at the discretion by the site principal investigator, and after approval by ASCOLT Trial Management Group
    - ANC ⩾ 1.0 x 10^9/L
    - Platelets ⩾ 100 x 10^9/L
    - Creatinine clearance ⩾ 30 mL/min

  - Total bilirubin ≤ 2.0 x the upper limit normal
  - AST & ALT ≤ 5 x the upper limit normal
- Completed the following investigations
  - Colonoscopy(or CT colonogram(within 16 months prior to randomization)
  - Imaging of abdomen (CT or CT colonogram or MRI or PET or Ultrasound) within 16 months prior to randomization
- Written informed consent

5.2. Exclusion Criteria

- Pre-existing Familial adenomatous polyposis, inflammatory bowel disease or ulcerative colitis
- Active gastritis or active peptic ulcer
- History of continuous daily use of PPI more than 1 year prior to consent
- Gastrointestinal bleeding within the past one year
- Haemorrhagic diathesis (i.e. haemophilia)
- Uncontrolled hypertension (untreated systolic blood pressure > 160 mmHg, or diastolic blood pressure > 95 mmHg)
- History of recent cancers (except for colorectal cancers, non-melanoma skin cancers, basal cell carcinomas, squamous cell carcinomas) in the past 5 years
- History of stroke, coronary arterial disease, angina, or vascular disease
- Patients who are on current long term treatment (≥ 4 consecutive weeks) with Aspirin, NSAID or Cox-2 inhibitors
- History of erosive GERD or active erosive GERD on gastroscopy.
- Patient on active current treatment of antiplatelet agents (i.e. off-study Aspirin, clopidogrel, ticlopidine)
- Patient receiving active treatment of anticoagulants (i.e. warfarin, low molecular weight heparins)
- Pregnant, lactating, or not using adequate contraception
- Patient having known allergy to NSAID or Aspirin
- Unexplained rise of CEA (i.e. smoker with elevated CEA will not be excluded)
- Patient on other investigational drug
- Patients with HNPCC (Lynch Syndrome)

6. RANDOMISATION

Randomization will occur within 120 days of completion of standard therapy. After informed consent is signed and subject's eligibility is confirmed, the subject can be randomised in a 1:1 allocation ratio, stratified by

- Study centre
- Tumour type (Dukes C colon, high risk Dukes B colon cancer, and rectal cancer sub-groups) and
- Type of adjuvant chemotherapy received (exposed/ not exposed to oxaliplatin)

to receive either Aspirin or a matched placebo for 3 years.

Randomisation will be done via direct web randomisation: Authorized study centre personnel will randomise the patient via a password-protected internet web site. The randomisation system will then determine the treatment arm and provide the subject number to be used for the patient. The site monitor/ project coordinator will be informed immediately in the event that the web randomisation is not successful.

7. STUDY DRUG

7.1. How to take the study drug

The study subject will take two tablets of study drug orally daily for 3 years or as instructed by investigator.

7.2. Supply and Resupply of study drug

The investigator or designated person at the study centre will dispense the study drug to the patient at baseline visit and each assessment/ follow-up visit. Patients are required to return the used and unused bottles at each visit for drug accountability.

Each subject will be given sufficient supply at each follow-up visit to the study centre according to the specified protocol schedule during the first 3 years after randomisation. If the subject requires treatment modification (dose reduction), study drug supply will be adjusted and dispensed as appropriate.

The manufacturer of study drug, Bayer AG, will provide the required study drugs for this study. These are being shipped from Bayer AG to the central drug depot. The central drug depot will then be responsible for the distribution to the individual study centres. The study centre PI is responsible to ensure that there are adequate supplies for the study subjects at the centre.

7.3. Description and contents of study drug and placebo drug

Each tablet contains 100 mg enteric-coated Aspirin (Acetylsalicylic acid) or placebo. Excipients of Aspirin Cardio placebo include: microcrystalline cellulose, citric acid anhydrous, dibasic hydrogen phosphate, lactose monohydrate, magnesium stearate of vegetable origin, maize starch, silica colloidal anhydrous, methacrylic acid-ethyl acrylate copolymer, talc, triethylcitrate.

7.4. Packaging information

Each tablet contains 100 mg enteric-coated Aspirin (Acetylsalicylic acid) or placebo. Study drug will be supplied in screw capped bottles with 100 tablets per bottle and two bottles of study drug will be packed in a secondary packaging box. This would be sufficient for 100 days for one subject.

7.5. Storage information

Study drugs at each centre must be stored in a secure and locked facility and in a dry place, at room temperature (25 degree centigrade and below). Any temperature deviation must be reported once known, to the site monitor or project coordinator who will in turn report to the project manager of the trial at NCCS.

Following exposure to a temperature deviation, study drugs will not be used until approval is given by the manufacturer through the trial project manager via the site monitor or project coordinator.

7.6. Study drug labelling and handling

The primary label of the study drugs will be labelled through Bayer AG centrally and delivered to the allocated depots. The kit numbers will be labelled by unblinded personnel at the allocated depots before delivering to the individual study centres in batches.

The label will include the following particulars (Appendix IV – Study Drug Label):

a) the study protocol code

b) batch number

c) Manufacture date, expiry date or retest date of study material

d) the subject number for whom the study material is intended

e) The study site code

f) Dosage and administration instruction

g) the storage conditions, as indicated by the manufacturer

h) the words : " For clinical trial use only"

i) the name and address of the manufacturer

The study drug at site should be handled only by authorised personnel at site. The study drug accountability will also be done by authorised personnel and all the procedures will be documented. The authorised personnel at site is also responsible of the destruction of the unused or returned study drug as per institution practice after the study drug accountability is documented checked.

The investigator at each study centre should ensure that the investigational product(s) are used only in accordance with the approved protocol.

7.7. Compliance of study drug

Noncompliance is defined as omission of more than 30% of days for the study period during which the patient is on the study drug. The reason for non-compliance will be documented.

7.8. Anticipated side effects of Aspirin

Side effects that occasionally occur are gastrointestinal disorders such as nausea, vomiting, diarrhoea, and slight gastrointestinal blood loss which is exceptional cases can lead to anaemia. Gastrointestinal ulcers may rarely develop, in some circumstances with haemorrhaging and

perforation. Dizziness and ringing in the ear can occur as symptoms of over dosage, especially in children and elderly patients.

Rare cases of hypersensitivity reactions (e.g. difficulty in breathing, skin reaction) can occur.

Isolated cases of liver and kidney function disturbances, and severe skin reactions have been reported.

The absolute annual increase risk attributable to Aspirin for major bleeding, major gastrointestinal bleeding and intracranial haemorrhage is estimated at 0.13%, 0.12% and 0.03% respectively. (McQuaid KR et al)

8. BLINDING

8.1. Blinding

This is a double-blind study. The patient, the study team including the investigator(s) and the sponsor will be blinded. However, study statistician who prepares the randomisation list and the designated unblinded personnel who are involved in the procedures of study drug packaging and labelling will not be blinded.

8.2. Code-break envelopes

The study statistician will prepare the sealed emergency code-break envelopes and distribute to the study centres accordingly. The envelopes will contain the treatment assignment, with the corresponding subject number printed on it. The sealed emergency code-break envelopes will be collected before site closure or when necessary to return to the study statistician for destruction.

8.3. Unblinding

The emergency code-break envelopes provided by the study statistician will be kept at site by investigator or designated person. In case of medical emergency, unblinding should be done to the required subject only, where the study code needs to be known prior to the further management of subject. At such, the study centre investigator or designated person will unblind the treatment given to the subject. The investigator will discuss the purpose of unblinding with the study co-chair, if possible, prior to unblind the study patient. The study statistician and monitor/project coordinator will be informed of the occurrence and reason for unblinding. In the event of unblinding, only the investigator treating the patient will be unblinded of the treatment code & it is his primary responsibility to keep the rest of the study team at his centre blinded of the treatment code.

9. TREATMENT SCHEDULE

9.1. Treatment groups

The study drug, oral Aspirin 200 mg/ placebo 200 mg OD should be started immediately but no later than 2 weeks after randomisation/baseline visit. The patient will be treated with the same study drug for 3 years.

9.2. Treatment modification

The treatment modifications listed in this section are for reference only. The investigator should modify the treatment according to clinical judgement and best medical practices.

Event	Severity	Action
Angioedema	Any	Stop study drug permanently
Anaphylaxis	Any	Stop study drug permanently
Generalized Rashes	Any	- Stop study drug for two weeks<br>- Consider to rechallenge at next follow up/ assessment if the rash is mild, or unlikely to be related to study drug
Epigastric discomfort	Mild	- Reduce dose to 100 mg of study drug<br>- Start PPI<br>- Review in one month. If symptoms persist, consider stopping study drug and gastroscopy (Treat as per Gastroscopic findings)
Epigastric discomfort	Moderate	- Stop study drug<br>- Start PPI<br>- Review in one month<br>- Rechallenge with lower dose (i.e. 100 mg) of study drug if symptoms resolved and continue long term PPI therapy.<br>- Consider gastroscopy if symptoms persist
Epigastric discomfort	Severe	- Stop study drug<br>- Start PPI<br>- Gastroscopy<br>- Treat as per Gastroscopic findings
Haematamesis	-	- Stop study drug permanently<br>- Start PPI<br>- Gastroscopy<br>- Treat as per Gastroscopic findings
Melenic stools	-	- Stop study drug permanently<br>- Start PPI<br>- Gastroscopy with/without colonoscopy<br>- Treat as per Gastroscopic findings
Asymptomatic drop in haemoglobin of > 2 g/dL from baseline	-	- Stop study drug<br>- Start PPI<br>- Gastroscopy/Colonoscopy<br>- Treat as per Gastroscopic findings

Gastroscopic Findings

Event	Severity	Action
Gastritis	Mild/<br>Moderate	- Stop study drug<br>- Start PPI<br>- Rechallenge with lower dose (i.e. 100 mg) of study drug 2- 4 weeks after therapy with PPI<br>- Helicobacter pylori eradication if present
Gastritis	Severe	- Stop study drug (for 4-8 weeks)<br>- Start with PPI

		- Consider rechallenge with lower dose (i.e. 100 mg) of study drug 4-8 weeks after therapy with PPI if symptoms resolved completely<br>- Helicobacter pylori eradication if present
Peptic ulcer	-	- Stop study drug permanently<br>- Start PPI<br>- Helicobacter pylori eradication if present
Oesophageal or Gastric Varices	-	- Stop study drug permanently<br>- Continue on follow up as per institutional practice<br>- Helicobacter pylori eradication if present
Normal findings in a symptomatic patient	-	- Consider other cause of symptoms<br>- Continue study drug at lower dose (i.e. 100 mg) together with PPI<br>- Consider to stop study drug if symptoms are severe and persistent despite treatment with PPI

Patients who are unable to tolerate 200 mg of study drug may have the dose reduced to 100 mg of study drug. The reason and date of dose reduction will be clearly documented. PPIs will be used in patients who have symptoms of epigastric discomfort. They should be given at adequate doses and continued for at least 3 months. PPIs are preferable to H2 antagonists. Antacids (i.e. magnesium trisilicate, magnesium carbonate) should not be given in place of PPIs; however, they may be used to supplement these agents. Patients who undergo gastroscopy should be screened for helicobacter pylori. Positive helicobacter pylori should be treated accordingly.

Patients who develop anaphylaxis, angioedema or gastrointestinal bleeding should stop the study drug immediately and should not undergo rechallenge to study drug.

Patients who need to undergo elective surgery or other interventional procedures may stop the study drug 5 days prior to surgery and recommence the study drug upon recovery (when haemostasis is secured or when the patient is able to take orally).

9.3. Treatment discontinuation

The investigator has the right to discontinue study treatment if he/she feels that it is in the best interests of the patient or due to safety concerns.

Study drug will be stopped immediately if there is disease recurrence confirmed by CT, histology or cytology, and in the event of an SAE unequivocally related to study drug (or at the discretion of the investigator if the relationship of SAE to the study drug cannot be established with certainty at the time of reporting).

The reasons for treatment discontinuation will be documented. Patients with their treatment discontinued will continue to be followed up until 5 years after randomisation.

Even if a patient chooses not to adhere to the treatment assignment or data collection schedule, every effort should be made to follow patient for the protocol objectives until the end of the study.

9.4. Study Withdrawal

All patients, including patients discontinued from study treatment, will continue to be followed up for 5 years after randomisation, unless the patient withdraws consent, is lost to follow up or death. The reason for withdrawal should be documented.

10. CONCOMITANT/CONTRAINDICATED MEDICATIONS

Patients are not permitted to take the following medications concurrently with the study drug. If required, patient should be removed from the study treatment with the reason clearly documented: -

- Other investigational drugs while on treatment with study drug
- Any anticancer treatment while on treatment with study drug
- Antiplatelet agents (off-study Aspirin, clopidogrel, ticlopidine) concurrently with study medication
- Anticoagulants (warfarin, unfractionated heparin or low molecular weight heparin) concurrently with study medication

Patients should be encouraged to use paracetamol or codeine phosphate for analgesia and to avoid NSAID whenever possible. Although short term intermittent NSAID use is allowed, NSAID should not be prescribed with study drug for more than 2 consecutive weeks. The use of NSAID must be clearly documented throughout the study.

Patients will be allowed concurrent PPIs, H2 antagonists or antacids.

Ongoing concomitant medication will be recorded from baseline visit date until 30 days after treatment discontinuation. Patients receiving study drug should maintain adequate contraception.

11. ASSESSMENTS AND FOLLOW-UP

11.1. Screening

Within 8 Weeks prior to randomisation

- Haematology: platelet count
- Creatinine
- LFT: serum bilirubin, AST, ALT

Within 16 months prior to randomization

- Full colonoscopy(or CT colonogram).
- Imaging of abdomen (CT or CT colonogram or MRI or PET or Ultrasound)
- Radiological examination of the Chest (including but not restricted to Chest x ray, CT thorax or PET scan)

11.2. Baseline

- Medical history: asthma, diabetes, ischaemic heart disease, stroke, gastrointestinal ulcers or bleeding, alcohol history, smoking
- Tumour History: stage, risk factors, treatment, and tumour response grade (for patients with neoadjuvant chemoradiation only)
- Family history of Colorectal cancer
- Record ongoing medication
- Vital signs: body weight, height, BP
- Haematology: haemoglobin (within 8 weeks prior to randomisation)
- CEA(latest reading)
- Randomization performed and study treatment commence

11.3. 3-monthly assessments

3-monthly patient assessments will be done at month 3, 6, 9, 12, 15, 18, 21, 24, 27, 30, 33, and 36. Assessments ± 1 month window will be allowed for each 3-monthly visit. Phone assessments are allowed at alternate 3-monthly assessments if patient is unable to return for site follow-up. The following will be performed at each assessment:

- CEA
- Hb (for month 6, 12, 18, 24, 30, 36 only)
- Record AE and SAE (See Section 13.2)
- Concomitant medication
- Concomitant NSAIDs
- Medical Events : (acute myocardial infarction, transient ischemic attack, stroke, new cancers and gastrointestinal bleeding)
- Recurrence and Salvage Chemotherapy

The following will be performed at each specified interval:

- Surveillance Colonoscopy/ CT colonogram at month 6 and 30 (optional) *
- Surveillance CT scan (abdomen ± pelvis) / or CT colonogram at month 6, 18 and 30 (optional)*

Off-site investigations (for example surveillance CEA, CT scan, colonoscopy) will be allowed and data from unscheduled investigations (for example CT scan or colonoscopy due to symptoms or during hospitalisations) may be recorded.

* The policy of surveillance CT scan and Colonoscopy (as described above) will be decided at the study centre level, by the study centre PI. However, these procedures wherever implemented, should be consistently applied to all trial subjects within a particular study centre.

Early Treatment Discontinuation follow-up

- Subjects with early treatment discontinuation (e.g. for toxicity) but without recurrence will continue with the same 3 monthly assessments except Hb that is not required after study drug treatment is discontinued.
- Subjects with recurrence (who may be receiving salvage treatment or palliative care) may be assessed at 6 monthly intervals in the clinic or via phone assessments if they are unable to return for 3 monthly clinic follow up. Hb, CEA, and concomitant medication data are not required after recurrence date.

11.4. 6-monthly assessments

6-monthly assessments will be done at month 42, 48, 54 and 60.

Assessments ± 1 month window will be allowed for each 6-monthly visit. The following will be performed at each assessment:

- CEA
- Death and related information if any
- Medical Events: (AMI, transient ischemic attack, stroke, new cancers and gastrointestinal bleeding)
- Concomitant NSAIDs
- Recurrence and salvage chemotherapy
- Subjects with recurrence (who may be receiving salvage treatment or palliative care) may be assessed via phone assessments if they are unable to return for clinic follow up. CEA is not required after recurrence date.

11.5. Unscheduled visits

Any additional visit to the scheduled follow-ups will be considered as unscheduled visit if it is of medical significance in the opinion of the treating physician or investigator.

At such visits, the patient will be assessed for:

- Recurrence
- AE and SAE
- Medical Events: (acute myocardial infarction, transient ischemic attack, stroke, new cancers and gastrointestinal bleeding)

11.6. Assessment of recurrence and salvage chemotherapy

For the patient with unequivocal radiological documented recurrence, the date of CT scan or any other radiological modality documenting unequivocal recurrence will be taken as the date of recurrence. Patients who have disease recurrence confirmed with histology or cytology, the date of recurrence will be taken as the date of biopsy or cytology collection.

For patients with local recurrence detected through colonoscopy/CT colonogram or digital rectal examination (DRE), without CT scan or histology, the date of colonoscopy or DRE will be taken as the date of recurrence.

An elevated CEA level or abnormal LFT will not be considered as acceptable evidence of colorectal cancer recurrence. However, a rising CEA trend should prompt the search for possible tumour recurrence.

At the time of colorectal cancer recurrence, the investigator should clearly indicate the site of tumour recurrence and the method of diagnosis.

Patients with tumour recurrence and commencing salvage chemotherapy will have the names and initiation date of each new chemotherapy regimen recorded.

11.7. Assessment of Medical Events

Medical events such as acute myocardial infarction, ischemic stroke (including transient ischemic attack), hemorrhagic stroke or new non-colorectal cancers will be recorded. Major gastrointestinal bleeding (requiring blood transfusion) will also be recorded.

11.8 Post Month 60 – Clinical Data

Subjects who have consented to allow data collection after month 60, will have their medical records reviewed every 12 months (or via phone contact), up to and at the time of the last patient last visit (month 60 or earlier) - at the site.

The following will be recorded:

- Death related information; if any
- Medical Events: (AMI, transient ischemic attack, stroke, new cancers and gastrointestinal bleeding)
- Concomitant NSAIDs
- Recurrence and salvage chemotherapy

12 STATISTICAL CONSIDERATIONS

12.1 Sample size calculation

<u>Initial sample size calculation</u>

The initial total trial size was 2660 patients, 1330 randomised to Aspirin group and 1330 randomised to Placebo group. In the sub-groups, there should be at least 2000 high risk Dukes B or Dukes C colon cancer patients, others are rectal cancer patients

It was assumed that 3-year disease free survival rate for Dukes B colon cancer, Dukes C colon cancer and rectal cancer are 65% after standard adjuvant chemotherapy; and the attrition rate is 5%. The total trial size (2660) would be sufficient to detect an 6% absolute difference of disease free survival rate for all subjects between the two treatments, with a two-sided logrank test of 5% type I error and 90% power; For the main sub-group analysis, the 3-year DFS rate for colon cancer patients

was assumed to be 65% under standard care, which is similar to the entire group as a whole, the size of colon cancer (n=2000) will be sufficient to detect an 6% absolute difference of disease free survival rate for colon cancer between the two treatments, with a two-sided logrank test of 5% type I error and 80% power.

Subsequent sample size calculation

Based on recent studies[31,33,34] of aspirin in colorectal cancer patients, with a reported hazard ratio (HR) or risk ratio ranging from 0.67 to 0.77, we assumed a hazard ratio of 0.72 for disease free survival between the aspirin and the placebo groups. The corresponding absolute difference of disease free survival rate between the two treatment groups is about 8.5% instead of 6% for a 3 year disease free survival rate of 65% in the placebo group. To detect a HR of 0.72, a total of 300 recurrences (or deaths if occur without recurrence) is required for a two-sided logrank test of 5% type I error and 80% power. This translates into a total sample size of 1200 patients if the disease free survival rate is 65% in the placebo group and attrition rate is 15%.

Latest sample size calculation

A recent review of trial interim data, after enrollment of at least 1140 patients and 3-years follow-up being completed in 430 patients, revealed a pooled disease free survival rate of 79% at 5 years. We estimated that only 240 recurrences would be observed with a total sample size of 1200, a shortfall of 60 recurrences to maintain the study power at 80%. We subsequently recalculated the sample size to be 1587 patients such that we would expect to observe 300 recurrences (or deaths) at the end of study (attrition rate reduced from 15% to 10%, the rest unchanged).

12.2 Statistical analysis

All statistical analyses will be carried out on an intention-to-treat basis.

In the analysis of disease free survival, an event of interest is considered to have occurred if a patient relapses or dies during the study period. The starting point for disease free survival is the date of randomisation and the end point is the date of first disease recurrence or date of death, whichever occurs first. Patients in whom there has been no evidence of disease after treatment are censored at the date of last follow-up. Similarly, the overall survival time is censored at the date when the patient is last known to be alive.

Each primary endpoint will be analysed as follows. Survival curves will be constructed using the Kaplan-Meier method. Life table estimates of 3 and 5 year survival rates will be calculated. The efficacy of Aspirin will be evaluated by the Hazard Ratio (HR) and its corresponding 95% CI. A Cox proportional hazard model will be used to estimate HRs adjusting for the trial stratification factors (site, type of tumour and type of adjuvant chemotherapy). Stratified analysis and other non-proportional hazard models (which allow for the effects of covariates to vary over time) would be considered when proportional hazards assumption is not valid.

The secondary endpoint of overall survival will be analysed in a similar manner to the primary endpoints. For the subgroup analyses, tests for interaction for the colon cancer subgroups and other subgroups will be conducted first. Similar analyses of DFS and OS as presented above will be repeated within the subgroups respectively defined by ethnicity, tumour type, and patients'

compliance and according to patient's PIK3CA mutational status. The percentage of patients with different PIK3CA mutational status will be compared by Chi-square test in the Aspirin and Placebo groups.

As an exploratory analysis, cumulative incidence function will be estimated and the two treatment arms will be compared using Gray's method when considering competing risks of first recurrence with different causes of death due to colorectal cancer, other cancers or other causes.

12.3 Interim analysis

An independent DMC will be established to review the interim results of the study. Two interim analyses are scheduled. Ideally, the first interim analysis should be done after 540 patients have been recruited (estimated to take between two to three years) or at the mid-point of the targeted recruitment period (end of Year 2012), whichever comes earlier. The second interim analysis should be done once 540 patients or the same cohort of patients from the first interim analysis, have been followed up for 3 years (approximately between fifth to sixth year).

The aim of the first interim analysis is toxicity. Safety will be the main issue reviewed in the first DMC meeting. A report containing non-confidential data should be sent to the Study Steering Committee. Randomisation, compliance, CRF received and processed, AE and SAE should be included in this DMC report.

The study endpoints to be reviewed in the second interim analysis are disease free survival and overall survival, in addition to toxicity profiles. Statistical analysis method for the primary endpoint will be the same as 12.2. The analysis results of the interim analyses will not be the sole criteria for deciding whether to terminate accrual or report the results early. Rather they will provide a guideline to aid in the decision, which will also take into account the characteristics of the patients, nature of toxicities, relevant external results. Another goal of this monitoring is to identify if the study is underpowered and sample size needs to be adjusted. Apart from the reason of safety, which may caution otherwise, the minimum trial size will remain as 1200 as initially planned.

In addition to the two interim analyses above, periodic review of safety data (in the same format of the first interim analysis) will be performed for annual DMC meetings. Efficacy data will not be included unless requested by DMC.

The results of the interim reviews will remain confidential to the study statistician and the DMC members. After reviewing the efficacy and safety data, the DMC will make recommendations to the Study Steering Committee on changes to the protocol.

13 SAFETY

The site investigator of each study centre is responsible for monitoring the safety of subjects for that study centre.

Immediate medical attention should be provided to resolve any SAE which occurs during the study**.** All AEs and SAEs occurring after any administration of the study drug will be followed until resolution or are clearly determined to be due to the patient's stable or chronic condition or intercurrent illness.

13.1 Definitions

Adverse event

An adverse event (AE) is any untoward medical occurrence in a patient administered a pharmaceutical product and which does not necessarily have a causal relationship with this treatment.

An AE can therefore be any unfavourable and unintended sign (including an abnormal laboratory finding, for example), symptom, or disease temporally associated with the use of a medicinal (investigational) product, whether or not considered related to the medicinal (investigational) product.

All AEs, including observed or volunteered problems, complaints, or symptoms are to be recorded. Each AE is to be evaluated for duration, intensity and causal relationship with the study medication or other factors.

The most anticipated AEs associated with Aspirin use are gastrointestinal related, majority of which are minor and resolved without any medical intervention. In rare cases, more serious effects, such as bleeding can be occurred in patient with long-term Aspirin use. Even less, haemorrhagic stroke can occur.

Adjuvant chemotherapy related toxicities can occur, however they are not regarded as AE for this study.

Serious adverse event

A serious adverse event (SAE) is any untoward medical occurrence that:

- Results in death
- Is life-threatening
- Requires inpatient hospitalisation or prolongation of existing hospitalisation
- Results in persistent or significant disability/incapacity, or
- Is a congenital anomaly/birth defect

A medical event that may not be immediately life-threatening or result in death or hospitalisation but may jeopardise the patient or may require intervention to prevent a serious outcome may also be considered serious (e.g. intensive treatment in an emergency room or at home for allergic bronchospasm; blood dyscrasias or convulsions that do not result in hospitalisation; or development of drug dependency or drug abuse). Medical and scientific judgement must be exercised when classifying events as serious.

When SAE occurs, the subject will be reassessed and investigator will consider whether to continue or discontinue study medication.

Unexpected adverse drug reaction (UADR)

An UADR is defined as an adverse reaction, the nature or severity of which is not consistent with the applicable product information (e.g. investigator’s brochure for an unapproved investigational product or package insert/summary of product characteristics for an approved product).

13.2 Reporting of Adverse Event

All AEs will be recorded on the AE form in the subject's case report form.

The site investigator (international site or local site) is responsible to report **all SAEs** with de-identified relevant reports **within 1 working day** of the knowledge of the event to all of the following:

- Attention to monitor or project coordinator
- Investigational Product Manufacturer (Bayer AG)

The site investigator is also responsible to report all SAEs to relevant ethics committee or institutional review board within the required timeline. All SAEs will be followed up until resolution with updated information reported whenever available to the relevant parties as per initial reporting procedure.

The study monitor/project coordinator will notify the study co-chair and study project manager with the submitted SAE report by the site. The study co-chair will review the SAE report and recommendations will be sent to the site investigators if any.

The study co-chair is responsible to ensure any serious and unexpected adverse drug reaction occurring in the study is notified to all participant sites for reporting to ethics committee(s) or institutional review board(s) of all study centres and regulatory body (ies) of the country (ies) where the study is conducted by using the CIOMS Form. Fatal or life-threatening unexpected adverse drug reaction must be notified immediately but no later than 7 calendar days after the first knowledge by the study co-chair, followed by a complete report within 8 additional calendar days. All other serious, unexpected adverse drug reactions that are not fatal or life threatening must be reported within 15 calendar days after first knowledge by the study co-chair. The CIOMS Form will be sent to Bayer AG for review before distributing to all participating sites.

After discontinuation of treatment, patients should be carefully monitored for the occurrence of new adverse events up to 30 calendar days (follow-up period) after the administration of the last dose of aspirin. Adverse events are to be assessed in terms of their seriousness, severity, and relationship to the study drug. Only serious adverse (SAEs) should be reported to Bayer AG.

AEs and SAEs will be recorded from the time of entry into the study until 30 days after the full completion of study treatment, or in instances of early drug termination 30 days after the final cessation of study drug.

13.3 Reporting of Death

The site investigator is responsible for reporting all deaths that happen after 30 calendar days of study drug discontinuation until study completion date within 14 days of first knowledge by the Site Investigator to the study monitor/project coordinator. Date of death, cause and related information - will be documented as specified by the Trial Management Group.

The study monitor/project coordinator will notify the study co-chair and study project manager with the submitted report by the site. The report will be reviewed by the study co-chair and recommendations will be sent to the site investigators if any.

14 SUB-STUDIES (For Singapore and Selected Sites Only)

14.1 Blood Samples

Only patients who consent for research bloods will have 30 mls of blood collected at the time of consent and at 6 and 12 months after randomisation. The blood will be spun down and the buffy coat and serum aliquoted and stored separately in liquid nitrogen and at minus 80 degrees respectively. Each sample will be coded by a study number with no patient identifiers.

14.1 Biomarkers, protein, lipid and leukocyte profiling

Biomarkers will be investigated for possible association with clinical outcomes (disease free survival, overall survival) and for the effects of the study medication. Protein biomarkers related to inflammation and adaptive immunity will be profiled using standardised high throughput techniques. Lipid biomarkers derived from the metabolism of fatty acids which may be affected by COX activity will be profiled using HPLC. Leukocyte subsets and their function will be characterised by flow cytometry.

14.2 Germline DNA

DNA will be extracted using standardised methods and stored for subsequent genotyping. Genotyping for polymorphisms in drug targets and metabolising enzymes of relevance to aspirin and susceptibility to or prognosis of colon cancer will be performed using previously described techniques. Known functional SNPs of *COX 1 and COX2*, Prostaglandin syntheses, and *CYP2C9* will be characterized using high throughput sequencing techniques.

14.3 Stool Samples

Stool Samples will be collected once at the following time points: Before initiation of study drug or any time points after initiation of study drug. Stool will be collected in a sterile specimen container and approximately one gram will be sealed in cryovials and snap frozen in liquid nitrogen before storage at -80 degrees Celsius. Stools will be analyzed in batches for Streptococcus *bovis* and other colonic flora. Association analysis will be made between colonic flora and clinical outcomes.

14.4 Urine Samples.

Urine specimens will be collected at the time of entry into the study before initiation of aspirin, and at 6 and 12 months after randomisation. Urine will be collected in a sterile specimen container and aliquot into vials to be stored at -80 degrees. Urine will be analysed for prostaglandin metabolites levels that will be correlated with aspirin treatment and outcomes.

14.5 Archival tumour specimens

In consenting patients, where samples are available, archived paraffin embedded tumour tissues will be retrieved, annotated, and stored till the end of the study where they will then be reviewed for immunohistocytochemical COX 1/ 2 staining and graded according to intensity of staining. Correlation will be made between intensity of Cox 1/ 2 staining and aspirin benefit. Archived tumour tissue will be stained for leukocyte subsets and correlation made with COX staining, survival and aspirin benefit.

DNA will also be extracted from archival paraffin tumour samples and tumour PIK3CA mutational status will also be assessed and analysed according to Aspirin exposure.

15 DIRECT ACCESS TO SOURCE DATA

The investigator will permit study related monitoring, audits, MCRC and/or EC review and regulatory inspection(s), providing direct access to the source data/ documents.

16 QUALITY CONTROL AND QUALITY ASSURANCE

This study will be monitored by delegated monitors according to its standard operating procedures. All protocol amendments will be in collaboration with SCRI. All administrative changes and regulatory reports/ approvals will be reported to Trial Management Group. The Investigator at each study centre must ensure that the study is conducted in compliance to the approved study protocol at the respective centre.

17 ETHICAL CONSIDERATIONS

This study shall be conducted in accordance to the principles of ICH E6 and any relevant research requirements. The study protocol should be reviewed and approved by the institutional review board or ethics committee of each participating centre. This will be the responsibility of each participating investigator.

The patient's written informed consent to participate in the study should be obtained after a full explanation has been given of the treatment options, including the conventional and generally accepted methods of treatment and the manner of treatment allocation.

The right of the patient to refuse to participate without giving reasons must be respected. After the patient has entered the study, the clinician remains free to give alternative treatment to that specified in the protocol at any stage if he/she feels it to be in the patient's best interest. However, the patient will need to remain within the study for the purpose of follow-up and data analysis. Similarly, the patient remains free to withdraw at any time from protocol treatment without giving reasons and without prejudicing his/her further treatment.

Records identifying the patient will be kept confidential and will not be made publicly available. If the results of the study are presented or published, the patient’s identity will be kept confidential. The monitor(s), auditor(s), the Ethic Committee/ Institutional Review Board, and the regulatory authority (ies) will be granted direct access to patient’s original medical records for verification of clinical study procedures and/or data, without violating the patient confidentiality.

18 INSURANCE

Study related injury would be determined by the institution insurance policy of the centre.

19 PUBLICATION POLICY

The results from all participating centres will be analysed together and published as soon as possible. Individual clinicians must not publish data concerning their patients that are directly relevant to

questions posed by the study until the main study report is published. The study results will be published under the ASCOLT Study Group.

20 STUDY ORGANISATION

The day-to-day running of the ASCOLT study will be managed by the Trial Management group. However, the ASCOLT study will also be subject to review by the Trial Steering Committee, Data Safety Monitoring Committee and Trial Management Group. Each committee will comprise of a mixture of independent members, study principal investigators, representatives from Trial Management Group, project manager and statisticians. For a full breakdown of committee members, please refer to the beginning of the protocol.

Data Monitoring Committee

The Data Monitoring Committee (DMC) is an independent group of individuals who are appropriately qualified and experienced in clinical trials. These individuals will be responsible for overseeing the safety aspects of the trial. The detailed plan of DMC review is stated under protocol 12.3 interim analysis. The DMC members will be confirmed within one year of study kick-off.

Trial Steering Committee

The Trial Steering Committee (TSC) is responsible for assessing the trial's overall progress, sample use, sanctioning or proposing any protocol changes, reviewing and approve publication data and abstracts, review trial endpoints, efficacy data, site performance, approving any proposals made by the TMG to change the design of the trial. This committee will receive and act upon reports from both the Trial Management Group and the DMC.

Trial Management Group

The Trial Management Group (TMG) is responsible for acting on the advice and recommendations of the DMC, and TSC. They will be responsible for acting on the advice and proposals given by the TSC and DMC committees, putting procedures in place, planning the course of action for the trial, problem solving any major issues and overall trial coordination.

21 ARCHIVING

The investigator of each centre must securely retain study documentation and secure access to the source data for a period of 15 years or as specified by the local IRB/ regulatory policy.

REFERENCES

1. Cancer Incidence, Mortality, and Prevalence Worldwide, GLOBOCAN, 2000 American Cancer Society.
2. Sung JY, Lau YW, Goh KL, et al. Increasing incidence of colorectal cancer in Asia: implications for screening. Lancet Oncol 2005;6:871-76.
3. Singapore Cancer Registry Interim Report: Trends in Cancer Incidence in Singapore 2002-2006. National Registry of Disease Office (NRDO).
4. Boyle P, Levin B. WHO 2008 World cancer Report. Internation Agency for Research on Cancer; Nonserial Publication, WHO Press 2008.
5. Ali R, Raina V. Developing innovative models for North-South cooperation in clinical research – exprience from the INDOX Cancer Research Network. Annals of Oncology 2008; 19: 831–833
6. Cavalli F. Cancer in the developing world: Can we avoid the disaster? Nat Clin Prac Oncol. 2006; 3(11):583-3.
7. Wolmark N; Fisher B; Rockette H et al. Postoperative adjuvant chemotherapy or BCG for colon cancer: results from NSABP protocol C-01. J Natl Cancer Inst 1988 Mar 2;80(1):30-6
8. Moertel CG; Fleming TR; Macdonald JS et al. Fluorouracil plus levamisole as effective adjuvant therapy after resection of stage III colon carcinoma: a final report. Ann Intern Med 1995 Mar 1;122(5):321-6.
9. O'Connell MJ; Mailliard JA; Kahn MJ et al. Controlled trial of fluorouracil and low-dose leucovorin given for 6 months as postoperative adjuvant therapy for colon cancer. J Clin Oncol 1997 Jan;15(1):246-50
10. Poplin EA; Benedetti JK; Estes NC et al. Phase III Southwest Oncology Group 9415/Intergroup 0153 randomized trial of fluorouracil, leucovorin, and levamisole versus fluorouracil continuous infusion and levamisole for adjuvant treatment of stage III and high-risk stage II colon cancer. J Clin Oncol. 2005; 20;23(9):1819-25
11. Carrato, A, Kohne, C, Bedenne et al. Folinic acid modulated bolus 5-FU or infusional 5-FU for adjuvant treatment of patients of UICC stage III colon cancer: Preliminarhy analysis of the PETACC-2-study. J Clin Oncol 2006; 24:161s
12. Chau I; Norman AR; Cunningham D et al. A randomised comparison between 6 months of bolus fluorouracil /leucovorin and 12 weeks of protracted venous infusion fluorouracil as adjuvant treatment in colorectal cancer. Ann Oncol. 2005 Apr;16(4):549-57
13. Twelves C; Wong A; Nowacki MP et al. Capecitabine as adjuvant treatment for stage III colon cancer. N Engl J Med 2005 Jun 30;352(26):2696-704.
14. Andre T; Boni C; Mounedji-Boudiaf L et al. Oxaliplatin, fluorouracil, and leucovorin as adjuvant treatment for colon cancer. N Engl J Med 2004 Jun 3;350(23):2343-51.
15. Van Cutsem E, Labianca R, Hossfeld D et al. Randomized phase III trial comparing infused irinotecan/5-fluorouracil (5-FU)/folinic acid (IF) versus 5-FU/FA (F) in stage III colon cancer patients (pts). (PETACC 3). J Clinl Oncol, 2005;23, (16S):8
16. M. Ychou, J.-L. Raoul, J.-Y. Douillard et al. A phase III randomized trial of LV5FU2+CPT-11 vs. LV5FU2 alone in adjuvant high risk colon cancer (FNCLCC Accord02/FFCD9802). J Clin Oncol. 2005;23(16S):3502.
17. L. B. Saltz, D. Niedzwiecki, D. Hollis et al. Irinotecan plus fluorouracil/leucovorin (IFL) versus fluorouracil/leucovorin alone (FL) in stage III colon cancer (intergroup trial CALGB C89803). J Clin Oncol. 2004;22(14S):3500.
18. Chau I, Cunningham D. Adjuvant chemotherapy for colon cancer – what, when and how. Ann Oncol 2006; 9: 1347-59.
19. Berges G, Benjamin LE. Tumorigenesis and the Angiogenic Switch. Nat Rev Cancer 2003; 3(6):401-10.
20. Fluorouracil, Leucovorin, and Oxaliplatin With or Without Bevacizumab in Treating Patients Who Have Undergone Surgery for Stage II or Stage III Colon Cancer. www.clinicaltrials.gov Study code : NCT00096278

21. Wolmark N, Yothers G, O Conell MJ et al. A Phase III trial comparing mFOLFOX6 to mFOLFOX6 pluse bevacizumab in stage II or III carcinoma of the colon: Results of NSABP Protocol C-08. J Clin Oncol 27:18s, 2009 (supple; abstr LBA4)

22. Comparison of Combination Chemotherapy Regimens With or Without Cetuximab in Treating Patients Who Have Undergone Surgery For Stage III Colon Cancer. www.clinicaltrials.gov Study code : NCT00079274

23. Combination Chemotherapy With or Without Cetuximab in Treating Patients With Stage III Colon Cancer That Was Completely Removed By Surgery. www.clinicaltrials.gov Study code : NCT00265811

24. Alberts SR, Sargent DJ, Smyrk TC, et al: Adjuvant mFOLFOX6 with or without cetuximab (Cmab) in KRAS wild-type (WT) patients (pts) with resected stage III colon cancer (CC): Results from NCCTG Intergroup Phase III Trial N0147. 2010 ASCO Annual Meeting. Abstract CRA3507. Presented June 5, 2010.

25. Williams CS, Mann M, DuBois RN. The role of cyclooxygenases in inflammation, cancer and development. Oncogene 1999; 18: 7908-16

26. Cai QY, Gao YT, Chow WH et al. Prospective Study of Urinary Prostaglandin E2 Metabolite and Colorectal Cancer Risk. J Clin Oncol 2006; 24:5010-5016.

27. Celecoxib Combined With Fluorouracil and Leucovorin in Treating Patients With Resected Stage III Adenocarcinoma (Cancer) of the Colon. www.clinicaltrials.gov Study code: NCT00085163

28. Rofecoxib After Surgery in Treating Patients With Stage II or Stage III Colorectal Cancer. www.clinicaltrials.gov Study code : NCT00031863

29. Midgley RSJ, McConkey CC, Langman MJ et al. VICTOR: A phase III placebo-controlled trial of Rofecoxib in colorectal cancer patients following surgical resection. Annals of Oncology 19 (Supplement 8): 2008, LBA 3.

30. Fuchs C, Meyerhardt D, Heseltine L. Influence of regular Aspirin on survival for patients with stage III colon cancer : Findings from Intergroup trial CALGB 89803. J Clin Oncol 2005;23S: 3530

31. Chan AT, Ogino S, Fuchs C. Aspirin use and survival after the diagnosis of Colorectal cancer. JAMA. 2009;302(6):649-659

32. Zell, J. A. et al. Non-steroidal anti-inflammatory drugs: effects on mortality after colorectal cancer diagnosis. Cancer 2009; 115: 5662–5671.

33. Bastiaannet, E. et al. Use of aspirin postdiagnosis improves survival for colon cancer patients. Br. J. Cancer 2012; 106: 1564–1570.

34. MacCowen C, Munro AJ, Donnan PT et al. Use of aspirin post-diagnosis in a cohort of patients with colorectal cancer and its association with all-cause and colorectal cancer specific mortality. Eur J Cancer 2012. http://dx.doi.org/10.1016/j.ejca.2012.10.024

35. Sandler R S, Halabi S, Baron J A et al. A randomised trial of Aspirin to prevent colorectal adenomas in patients with previous colorectal cancer. N Engl J Med 2003;348 : 883-90.

36. Baron J A, Cole B F, Sandler R S et al. A Randomised Trial of Aspirin to Prevent Colorectal Adenomas**.** N Engl J Med 2003; 348:891-899.

37. Logan RF; Grainge MJ; Shepherd VC et al. Aspirin and folic acid for the prevention of recurrent colorectal adenomas. Gastroenterology 2008; 134:29-38

38. Meyskens FL, McLaren CE, Pelot D et al. Difluoromethylornithine plus sulindac for the prevention of sporadic colorectal adenomas: a randomized placebo-controlled, double-blind trial. Cancer Prev Res 2008;1:32

39. Tuma Rabiya. Drugs to prevent Colon Cancer show promise but hurdles Remain for chemoprevention. J Nat Cancer Inst. 2008; 100 (11) : 764-6

40. Bertagnolli MM, Eagle CJ; Zauber AG et al. Celecoxib for the prevention of sporadic colorectal adenomas. N Engl J Med. 2006;355:873

41. Arber N; Eagle CJ; Spicak J et al. Celecoxib for the prevention of colorectal adenomatous polyps. N Engl J Med. 2006;355:885

42. Baron JA; Sandler RS; Bresalier RS et al. A randomized trial of rofecoxib for the chemoprevention of colorectal adenomas. Gastroenterology 2006;131:1674

43. Antithrombotic Trialists' Collaboration. Collaborative meta-analysis of randomised trials of antiplatelet therapy for prevention of death, myocardial infarction, and stroke in high risk patients. BMJ 2002 Jan 12;324(7329):71-86

44. Berger JS; Brown DL; Becker RC. Low-dose aspirin in patients with stable cardiovascular disease: a meta-analysis. Am J Med. 2008 Jan;121(1):43-9.

45. Impreriale T F. Aspirin and the Prevention of colorectal cancer. N Engl J Med 2003; 348(10):879-880.

46. Kune GA, Kune S, Watson LF. Colorectal Cancer Risk, Chronic Illnesses, Operations and Medications: Case Control Results from the Melbourne Colorectal Cancer Study. Cancer Res 1988 ;48:4399

47. Thun MJ, Namboodiri MM, Calle EE et al. Aspirin Use and Risk of Fatal Cancer. Cancer Res 1993; 15; 53 (6): 1322-7

48. Schreinemachers DM, Everson RB. Aspirin use and lung, colon, and breast cancer incidence in a prospective study. Epidemiology. 1994; 5(2):138-46

49. Giovannucci E, Edgan KM, Hunter D et al. Aspirin and the risk of Colorectal cancer in women. N Engl J Med 1995; 333 : 609-14.

50. Flossmann E, Rothwell PM. Effect of Aspirin on long-term risk of colorectal cancer: consistent evidence from randomised and observational studies. Lancet; 369:1603.

51. Cook NR, Lee IM, Gaziano JM et al. Low-Dose Aspirin in the Primary Prevention of Cancer: The Women’s Health Study: A Randomized Controlled Trial. JAMA 2005;294:47Fuchs C, Meyerhardt D, Heseltine L. Influence of regular Aspirin on survival for patients with stage III colon cancer : Findings from Intergroup trial CALGB 89803. J Clin Oncol 2005;23S: 3530

52. Thun MJ, Henley SJ, Patrono C. Nonsteroidal Anti-inflammatory Drugs as Anticancer Agents: Mechanistic, Pharmacologic, and Clinical Issues. J Natl Cancer Inst 2002;94: 252-66

53. Wang D, DuBois RN. Prostaglandins and Cancer. Gut 2006;55:115-22

54. Reddy BS; Rao CV; Rivenson A; Kelloff G. Inhibitory effect of aspirin on azoxymethane-induced colon carcinogenesis in F344 rats. Carcinogenesis 1993;14(8):1493-7.

55. Pollard M; Luckert PH. Indomethacin treatment of rats with dimethylhydrazine-induced intestinal tumors. Cancer Treat Rep 1980;64(12):1323-7.

56. Narisawa T; Sato M; Tani M et al. Inhibition of development of methylnitrosourea-induced rat colon tumors by indomethacin treatment. Cancer Res 1981; 41(5):1954-7.

57. Barnes CJ; Lee M. Chemoprevention of spontaneous intestinal adenomas in the adenomatous polyposis coli Min mouse model with aspirin. Gastroenterology 1998; 114(5):873-7.

58. Tsujii M, Kawano, Tsuji S et al. Cyclooxygenase Regulates Angiogenesis Induced by Colon Cancer Cells. Cell, 1998; 93: 705–716.

59. Fu SL, Wu YL, Zhang YP et al. Anti-cancer effects of COX-2 inhibitors and their correlation with angiogenesis and invasion in gastric cancer. World J Gastroenterol 2004;10(13):1971-1974

60. Robinson-Smith TM, Isaacsohn I, Mercer CA et al. Macrophages Mediate Inflammation-Enhanced Metastasis of Ovarian Tumors in Mice. Cancer Res 2007;67(12):5708–16

61. Pages F, Berger A, Sanchez CF et al. Effector memory T cells, early metastasis and survival in colorectal cancer. N Engl J Med 2005; 353:2654.

62. Schwertfeger KL, Xian W, Kaplan AM et al. A Critical Role for the Inflammatory Response in a Mouse Model of Preneoplastic Progression. Cancer Res 2006; 66(11): 5676-85

63. Hanahan D and Weinberg RA. Hallmarks of Cancer – The Next Generation. Cell 2011;144: 646-674.

64. Hahn MA, Hahn T, Lee DH et al. Methylation of Polycomb Target Genes in Intestinal Cancer Is Mediated by Inflammation. Cancer Res 2008;68(24):10280–9.

65. Castellone MD; Teramoto H; Williams BO et al. Prostaglandin E2 promotes colon cancer cell growth through a Gs-axin-beta-catenin signaling axis. Science. 2005 Dec 2;310(5753):1504-10

66. Jinyi S, Chaeyong J Chunming L. Prostaglandin E2 stimulates the b-catenin/T Cell Factor-dependent Transcription in colon cancer. J Bio Chem 2005; 28: 26565-72.

67. Kawamori T, Kitamura T, Watanabe K et al. Prostaglandin E receptor subtype EP1 deficiency inhibits colon cancer development. Carcinogenesis 2005; 26(2) : 353-7.

68. Cai QY, Gao YT, Chow WH et al. Prospective Study of Urinary Prostaglandin E2 Metabolite and Colorectal Cancer Risk. J Clin Oncol 2006; 24:5010-5016.

69. Crozier JEM, McKee RF, McArdle CS et al. Preoperative but not postoperative systemic inflammatory response correlates with survival in colorectal cancer. British Journal of Surgery 2007; 94:1028–1032

70. Ruffin MT 4th, Krishnan K, Rock CL. Supression of human colorectal mucosal prostaglandins: determining the lowest effective Aspirin dose. J Natl Cancer Inst. 1997; 89(15):1152-60.

71. Sample D, Wargovich M, Fischer SM et al. A dose-finding study of Aspirin for chemoprevention utilizing rectal mucosal prostaglandin E(2) levels as a biomarker. Cancer Epidemiol Biomarkers Prev. 2002;11(3):275-9.

72. Williams CS, Mann M, DuBois RN. The role of cyclooxygenases in inflammation, cancer, and development. Oncogene 1999; 18: 7908-16

73. Willams CS, Tsujii M, Reese J et al. Host cyclooxygenase-2 modulates carcinoma growth. Journal of Clinical Investigation 2000; 105 (11): 1589 -94.

74. Kashi K, Rigas B. Is COX-2 a ‘collateral’ target in cancer prevention? Biochemical Society Transactions 2005;33(4) : 724-7.

75. Liao XL, Lochhead P, Nishihara R et al. Aspirin Use, Tumor PIK3CA mutation and Colorectal-Cancer Survival. N Engl J Med 2012; 367: 1596-1606.

APPENDIX 1 – TNM AND DUKES STAGING FOR COLORECTAL CANCER

TNM and Dukes Staging for Colorectal Cancer

Primary Tumour (T)

Tx	Primary tumour cannot be assessed
T0	No evidence of primary tumour
Tis	Carcinoma in situ: intraepithelial or invasion of lamina propria*
T1	Tumour invades submucosa
T2	Tumour invades muscularis propria
T3	Tumour invades through the muscularis propria into the subserosa, or into non-peritonealized pericolic or perirectal tissues
T4	Tumour directly invades other organs or structures, and/or perforates visceral peritoneum** , ***

* Note: Tis includes cancer cells confined within the glandular basement membrane (intraepithelial) or lamina propria (intramucosal) with no extension through the muscularis mucosae into the submucosa.

** Note : Direct invasion in the T4 includes invasion of other segments of the colorectum by way of the serosa; for example, invasion of the sigmoid colon by a carcinoma of the cecum.

*** Tumour that is adherent to other organs or structures, macroscopically, is classified T4. However, if no tumour is present in the adhesion, microscopically, the classification should be pT3. The V and L substaging should be used to identify the presence or absence of vascular or lymphatic invasion.

Regional Lymph Nodes (N)

Nx	Regional lymph nodes cannot be assessed
N0	No regional lymph node metastasis
N1	Metastasis in 1 to 3 regional lymph nodes
N2	Metastasis in 4 or more regional lymph nodes

Note: A tumour nodule in the pericolorectal adipose tissue of a primary carcinoma without histologic evidence of residual lymph node in the nodule is classified in the pN category as a regional lymph node. If the nodule has an irregular contour, it should be classified in the T category and also coded as V1(microscopic venous invasion) or as V2 (if it was grossly evident), because there is a strong likelihood that it represents venous invasion.

Distant Metastasis (M)

Mx	Distant metastasis cannot be assessed
M0	No distant metastasis
M1	Distant metastasis

APPENDIX 1(Continued)

TNM AND DUKES STAGING FOR COLORECTAL CANCER

Stage grouping

T	N	M	TNM	Dukes
Tis	N0	M0	0	-
T1<br>T2	N0<br>N0	M0<br>M0	I<br>I	A<br>A
T3<br>T4	N0<br>N0	M0<br>M0	IIA<br>IIB	B<br>B
T1-T2<br>T3-T4<br>Any T	N1<br>N1<br>N2	M0<br>M0<br>M0	IIIA<br>IIIB<br>IIIC	C<br>C<br>C
Any T	Any N	M1	IV	-

Adapted from:
AJCC Cancer Staging Manual. Sixth Edition. Springer 2002.

Criteria for high risk Dukes B (any of the following)

- High grade (3 or 4) tumours
- Lymphatic invasion
- Vascular invasion
- Less than 12 lymph nodes examined
- Bowel obstruction
- Perforation
- T4 tumour
- Elevated CEA

APPENDIX 2 – ECOG PERFORMANCE STATUS

ECOG PERFORMANCE STATUS*

Grade	ECOG
0	Fully active, able to carry on all pre-disease performance without restriction
1	Restricted in physically strenuous activity but ambulatory and able to carry out work of a light or sedentary nature, e.g. light house work, office work
2	Ambulatory and capable of all self care but unable to carry out any work activities. Up and about more than 50% of waking hours
3	Capable of only limited self care, confined to bed or chair more than 50% of waking hours
4	Completely disabled. Cannot carry on any self care. Totally confined to bed or chair
5	Dead

* As published in Am. J. Clin. Oncol.:
Oken, M.M., Creech, R.H., Tormey, D.C., Horton, J., Davis, T.E., McFadden, E.T., Carbone, P.P.: Toxicity And Response Criteria Of The Eastern Cooperative Oncology Group. Am J Clin Oncol 5:649-655, 1982.

APPENDIX 3 – STUDY VISIT SCHEDULES & ASSESSMENTS

Assessments	Screening*		Baseline	Follow-up month (+/- 1 month)			Follow-up month (+/- 1 month) After Recurrence	Follow-up month (+/- 1 month) After Early Study Drug Discontinuation and no recurrence	Post Month 60
				3, 9 ,15, 21, 27, 33	6,12, 18, 24, 30, 36	42, 48, 54, 60	3 to 60	3 to 36	
Written informed consent		x							
Demography, Tumour history, Medical history & family history			x						
Record surgical measurements,(For rectal cancer only)			x						
Adjuvant Therapy			x						
Vital signs (Body weight, BP, height), ECOG			*x*						
Hb	(Within 8 weeks prior to randomisation)		x		x				
Platelet count, Creatinine, Serum Bilirubin, AST, ALT	(Within 8 weeks prior to randomisation)	x							
CEA			x	x	x	x		x	
Imaging of abdomen (CT or CT colonogram or MRI or PET or Ultrasound)	To be done if not done within 16 months prior to randomization	x		x (Mth 6, 18 and 30) Optional; or frequency according to institutional practice					
Radiological examination of the Chest (including but not restricted to Chest x ray, CT thorax or PET scan)	To be done if not done within 16 months prior to randomization	x							
Colonoscopy/CT colonogram	Full colonoscopy if not done within 16 months prior to randomization	x		x (Mth 6, 18 and 30) Optional; or frequency according to institutional practice					
AE and SAE				x	x				
Death				x	x	x	x	x	x
Medical Events				x	x	x	x	x	x
Concomitant NSAIDs				x	x	x	x	x	x
Concomitant treatment/medication				x	x				
Recurrence, Salvage Chemotherapy				x	x	x	x	x	x

Notes:

* Screening can start after last dose of standard therapy

APPENDIX 4 – STUDY DRUG LABEL

Study code :ICR02/ASCOLT<br><br>Date of Dispensing<br><br>Subject No:<br><br>Subject Initial<br><br>Kit No:<br><br>Pack Batch No:<br><br>Expiry date<br><br>2 bottles x 100 tablets Aspirin/Placebo 100mg<br>For Oral Use Only<br>Take as directed/<br>Do not store above 25°C<br><br>FOR CLINICAL TRIAL USE ONLY<br><br>KEEP OUT OF REACH OF CHILDREN<br><br>Manufactured by Bayer Pharma AG D-51368<br><br>Leverkusen, Germany
