# Supplementary material for: Adjuvant aspirin for colorectal cancer with PIK3CA-mutated and COX-2 overexpressed tumours: the ASCOLT translational research study and meta-analysis
Source: eBioMedicine. 2026 Jul 20;130:106389. doi: 10.1016/j.ebiom.2026.106389 (PMC13393707; doi:10.1016/j.ebiom.2026.106389)
Supplement: ASCOLT_TR_SAP [file mmc3.pdf]

ASCOLT STATISTICAL ANALYSIS PLAN

ASPIRIN FOR DUKES C AND HIGH RISK DUKES B COLORECTAL CANCERS

Prepared by: Isabel Li

2025-08-15

INTRODUCTION

The ASCOLT trial was designed to assess the effectiveness of aspirin compared with placebo on improving disease-free survival for patients with Dukes C or high risk Dukes B colorectal cancer. The main trial aimed to assess of treatment among all randomised patietns that commenced study medication in a modified intention-to-treat analysis.

The ASCOLT translational study aimed to assess the effects of among subsets of patients that had tissue available from the original tumour for analysis of genetic or molecular subtypes.

This analysis plan outlines the proposed analyses related to subsets of patients defined by 1. PIK3CA mutations 2. Related PIK mutated subtypes and 3. Cox II Overexpression.

PRIMARY OBJECTIVE

To compare DFS between the group randomised to Aspirin and the group randomised to placebo control in subjects with Dukes C or high-risk Dukes B colorectal cancer among patients with available tissue samples for PIK3CA/PTEN and COX-2 analysis.

SECONDARY OBJECTIVES

To compare OS between the group randomised to Aspirin and the group randomised to placebo control in subjects with Dukes C or high-risk Dukes B colorectal cancer among patients with available tissue samples for PIK3CA/PTEN and COX-2 analysis.

STUDY POPULATION

The study population consists of subjects with Dukes C colon cancer, high-risk Dukes B colon cancer, Dukes B rectal cancer, or Dukes C rectal cancer who have completed resection of the primary tumour. The population will receive standard therapy (chemotherapy ± radiotherapy). During the last cycle of standard therapy, patients will be invited to participate in this study. Once informed consent has been obtained, and within 120 days of completing standard therapy, they will be assessed for trial eligibility. Only paitents who have provided tissue samples for PIK3CA/PTEN and COX-2 analysis will be included in the analysis.

OTHERS

Please see protocol for details on the study design, randomisation, blinding, and other aspects of the trial.

STATISTICAL ANALYSIS PLAN

PATIENT STATUS

The patient status table will provide information about patient eligibility, consent, and treatment status. The following variables will be included in the analysis:

- Eligibility (n(%) for Yes, No, Unknown)
- Consent at study enrollment (n(%) for Yes, No, Unknown)

- Consent for post 5-year follow-up (n(%) for Yes, No, Unknown)
- Protocol treatment status (n(%) for Completed, Ongoing, Discontinued, Not Initiated, Unknown)
- Study completion status (n(%) for Completed, Ongoing, Discontinued, Not Initiated, Unknown)

RECRUITMENT - FOR ALL M-ITT PATIENTS (N = .)

RECRUITMENT BY COUNTRY

	m-ITT with Available Tissue[1]
	N = .[2]
**Country**	.
Australia	.
New Zealand	.
Singapore	.
Malaysia	.
Taiwan	.

[1]Modified intention-to-treat (mITT) with available tissue population consists of all subjects who signed the informed consent form for the study enrollment, commenced study treatment after the randomisation and have available tissue sample for assessments.

[2]n (%)

RECRUITMENT BY SITES

Country	m-ITT with Available Tissue
	Total patients = .[1]
**Australia**	.
AU - Austin Health Cancer Clinical Trials, VIC	.
BC - Bankstown Cancer Centre, NSW	.
BH - Barwon Health Andrew Love Cancer Centre, VIC	.
BM - Border Medical Oncology Research Unit, VIC	.
BR - Ballarat Regional Integrated Cancer Centre, NSW	.
CG - Central Coast Cancer Centre, NSW	.
CL - Chris O'Brien Lifehouse, NSW	.
CN - Calvary Mater Newcastle Hospital, NSW	.
GV - Goulburn Valley Health, VIC	.
LE - Northern Cancer Institute, St Leonards	.
LG - Launceston General Hospital, VIC	.
LM - Lyell McEwin Hospital, SA	.
MB - Mildura Base Hospital, VIC	.
MC - Macarthur Cancer Therapy Centre, NSW	.
MH - Monash Health Medical Oncology, VIC	.
NI - Coffs Harbour North Coast Cancer Institute, NSW	.
NP - Newcastle Private Hospital, NSW	.
NW - Northwest Cancer Centre Tamworth, NSW	.
OH - Orange Health Service, NSW	.
PM - North Coast Cancer Institute Port Macquarie, NSW	.
RB - Royal Brisbane and Women's Hospital, QLD	.
RD - Royal Darwin Hospital, Alan Walker Cancer Centre	.
RH - Royal Hobart Hospital, TAS	.
SC - Sir Charles Gairdner Hospital, WA	.
SJ - St John of God Subiaco, WA	.
SO - St John of God Healthcare Southwest Oncology	.
St Vincent's Hospital, The Kinghorn Cancer Centre, Sydney	.
SV - St Vincent's Hospital, The Kinghorn Cancer Centre, Sydney	.
TH - The Tweed Hospital, NSW	.
TO - Toowoomba Hospital Cancer Care Services, Queensland	.
TV - Townsville Hospital, QLD	.
**New Zealand**	.
CR - Christchurch Public Hospital, Christchurch	.
DU - Dunedin Hospital, Dunedin	.
**Singapore**	.
NC - National Cancer Centre (Singapore)	.
**Malaysia**	.
UM - University Malaya Medical Centre, KL (Malaysia)	.

Country	m-ITT with Available Tissue<br>Total patients = .[1]
**Taiwan**	.
Shuang Ho Hopistal (Taiwan)	.

[1]n / N (%)

STRATIFICATION FACTORS

	m-ITT with Available Tissue[1]		
	**Overall** N = .[2]	**Placebo** N = .[2]	**Aspirin** N = .[2]
**Chemotherapy type**	.	.	.
Exposed to oxaliplatin	.	.	.
Not exposed to oxaliplatin	.	.	.
**Tumour type**	.	.	.
Dukes c colon cancer	.	.	.
High risk dukes b colon cancer	.	.	.
Rectal cancer (dukes b or dukes c)	.	.	.
**Country**	.	.	.
Australia	.	.	.
New Zealand	.	.	.
Singapore	.	.	.
Malaysia	.	.	.
Taiwan	.	.	.

[1]Modified intention-to-treat (mITT) with available tissue population consists of all subjects who signed the informed consent form for the study enrollment, commenced study treatment after the randomisation and have available tissue sample for assessments.

[2]n (%)

BASELINE CHARACTERISTICS OF PATIENTS BY TREATMENT ARM

The baseline characteristics of the patients will be summarised descriptively The following variables will be included in the analysis(see tables)

	m-ITT with Available Tissue[1]		
	**Overall** N = .[2]	**Placebo** N = .[2]	**Aspirin** N = .[2]
**Age**	.	.	.
Mean (SD)	.	.	.
Median (Q1 - Q3)	.	.	.
Min - Max	.	.	.
**Age group**	.	.	.
<=70	.	.	.
>70	.	.	.
**Gender**	.	.	.
Female	.	.	.
Male	.	.	.
**Ethnicity**	.	.	.
East Asian	.	.	.
South Asian	.	.	.
Caucasian	.	.	.
Others	.	.	.
Unknown	.	.	.
**Country**	.	.	.
Australia	.	.	.
New Zealand	.	.	.
Singapore	.	.	.
Malaysia	.	.	.
Taiwan	.	.	.
**Diet**	.	.	.
Non-vegetarian	.	.	.
Vegetarian (including egg)	.	.	.
Unknown	.	.	.
**ECOG**	.	.	.
0	.	.	.
1	.	.	.
2	.	.	.
**Time from surgery to randomisation (months)**	.	.	.
Mean (SD)	.	.	.
Median (Q1 - Q3)	.	.	.
Min - Max	.	.	.
**Primary tumour location**	.	.	.
Ascending colon	.	.	.
Caecum	.	.	.
Descending colon	.	.	.
Hepatic flexure	.	.	.

	m-ITT with Available Tissue[1]		
	**Overall** N = .[2]	**Placebo** N = .[2]	**Aspirin** N = .[2]
Rectum	.	.	.
Sigmoid colon/ rectosigmoid colon	.	.	.
Splenic flexure	.	.	.
Transverse colon	.	.	.
Unknown	.	.	.
**T stage**	.	.	.
T1	.	.	.
T2	.	.	.
T3	.	.	.
T4	.	.	.
Unknown	.	.	.
**N stage**	.	.	.
0	.	.	.
1	.	.	.
2	.	.	.
Unknown	.	.	.
**Tumour type**	.	.	.
Dukes c colon cancer	.	.	.
High risk dukes b colon cancer	.	.	.
Rectal cancer (dukes b or dukes c)	.	.	.
**Chemotherapy regimen**	.	.	.
5-fu bolus	.	.	.
Capecitabine	.	.	.
Capecitabine - oxaliplatin	.	.	.
Folfox	.	.	.
-usional 5-fu	.	.	.
Others	.	.	.
Unknown	.	.	.
**Chemotherapy cycle duration (weeks)**	.	.	.
Mean (SD)	.	.	.
Median (Q1 - Q3)	.	.	.
Min - Max	.	.	.
Unknown	.	.	.
**No. of cycles completed**	.	.	.
Mean (SD)	.	.	.
Median (Q1 - Q3)	.	.	.
Min - Max	.	.	.
Unknown	.	.	.
**Time from surgery to first chemotherapy (days)**	.	.	.
Mean (SD)	.	.	.

	m-ITT with Available Tissue[1]		
	**Overall** N = .[2]	**Placebo** N = .[2]	**Aspirin** N = .[2]
Median (Q1 - Q3)	.	.	.
Min - Max	.	.	.
Unknown	.	.	.
**Radiotherapy regimen (for rectal cancer only)**	.	.	.
Adjuvant chemo rt	.	.	.
Neoadjuvant chemo rt	.	.	.
Neoadjuvant rt	.	.	.
No rt	.	.	.
Unknown	.	.	.
**Time from radiotherapy to randomisation (months)(for rectal cancer only)**	.	.	.
Mean (SD)	.	.	.
Median (Q1 - Q3)	.	.	.
Min - Max	.	.	.
Unknown	.	.	.
**Dose of radiotherapy (for rectal cancer only)**	.	.	.
Mean (SD)	.	.	.
Median (Q1 - Q3)	.	.	.
Min - Max	.	.	.
Unknown	.	.	.
**No. of radiotherapy fractions (for rectal cancer only)**	.	.	.
Mean (SD)	.	.	.
Median (Q1 - Q3)	.	.	.
Min - Max	.	.	.
Unknown	.	.	.
**Tumour type**	.	.	.
Dukes c colon cancer	.	.	.
High risk dukes b colon cancer	.	.	.
Rectal cancer (dukes b or dukes c)	.	.	.

[1]Modified intention-to-treat (mITT) with available tissue population consists of all subjects who signed the informed consent form for the study enrollment, commenced study treatment after the randomisation and have available tissue sample for assessments.

[2]n (%)

AUSTRALIA

Same table structure as above

NEW ZEALAND

Same table structure as above

SINGAPORE

Same table structure as above

MALAYSIA

Same table structure as above

TAIWAN

Same table structure as above

BASELINE CHARACTERISTICS OF PATIENTS BY BIOMARKER STATUS

	PIK3CA mutation[1]		Cox-2 Expression[1]		Cox-2 Expression (Ordinal scale)[1]				PIK3CA/PTEN mutation[1]		PIK3CA mutation (Exon 9, 20)[1]		PTEN mutation[1]		HLA class I expression[1]				
	**No or Unknown** N = .[2]	**Yes** N = .[2]	**Negative** N = .[2]	**Positive** N = .[2]	**Absent** N = .[2]	**Weak** N = .[2]	**Moderate** N = .[2]	**Strong** N = .[2]	**No or Unknown** N = .[2]	**Yes** N = .[2]	**No** N = .[2]	**Yes** N = .[2]	**No or Unknown** N = .[2]	**Yes** N = .[2]	**<5%** N = .[2]	**5-25%** N = .[2]	**25-50%** N = .[2]	**50-75%** N = .[2]	**>75%** N = .[2]
**Age**	.	.	.	.	.	.	.	.	.	.	.	.	.	.	.	.	.	.	.
Mean (SD)	.	.	.	.	.	.	.	.	.	.	.	.	.	.	.	.	.	.	.
Median (Q1 - Q3)	.	.	.	.	.	.	.	.	.	.	.	.	.	.	.	.	.	.	.
Min - Max	.	.	.	.	.	.	.	.	.	.	.	.	.	.	.	.	.	.	.
**Age group**	.	.	.	.	.	.	.	.	.	.	.	.	.	.	.	.	.	.	.
<=70	.	.	.	.	.	.	.	.	.	.	.	.	.	.	.	.	.	.	.
>70	.	.	.	.	.	.	.	.	.	.	.	.	.	.	.	.	.	.	.
**Gender**	.	.	.	.	.	.	.	.	.	.	.	.	.	.	.	.	.	.	.
Female	.	.	.	.	.	.	.	.	.	.	.	.	.	.	.	.	.	.	.
Male	.	.	.	.	.	.	.	.	.	.	.	.	.	.	.	.	.	.	.
**Ethnicity**	.	.	.	.	.	.	.	.	.	.	.	.	.	.	.	.	.	.	.
East Asian	.	.	.	.	.	.	.	.	.	.	.	.	.	.	.	.	.	.	.
South Asian	.	.	.	.	.	.	.	.	.	.	.	.	.	.	.	.	.	.	.
Caucasian	.	.	.	.	.	.	.	.	.	.	.	.	.	.	.	.	.	.	.
Others	.	.	.	.	.	.	.	.	.	.	.	.	.	.	.	.	.	.	.
Unknown	.	.	.	.	.	.	.	.	.	.	.	.	.	.	.	.	.	.	.
**Country**	.	.	.	.	.	.	.	.	.	.	.	.	.	.	.	.	.	.	.
Australia	.	.	.	.	.	.	.	.	.	.	.	.	.	.	.	.	.	.	.
New Zealand	.	.	.	.	.	.	.	.	.	.	.	.	.	.	.	.	.	.	.
Singapore	.	.	.	.	.	.	.	.	.	.	.	.	.	.	.	.	.	.	.
Malaysia	.	.	.	.	.	.	.	.	.	.	.	.	.	.	.	.	.	.	.
Taiwan	.	.	.	.	.	.	.	.	.	.	.	.	.	.	.	.	.	.	.
**Diet**	.	.	.	.	.	.	.	.	.	.	.	.	.	.	.	.	.	.	.

	PIK3CA mutation[1]		Cox-2 Expression[1]		Cox-2 Expression (Ordinal scale)[1]				PIK3CA/PTEN mutation[1]		PIK3CA mutation (Exon 9, 20)[1]		PTEN mutation[1]		HLA class I expression[1]				
	**No or Unknown** N = .[2]	**Yes** N = .[2]	**Negative** N = .[2]	**Positive** N = .[2]	**Absent** N = .[2]	**Weak** N = .[2]	**Moderate** N = .[2]	**Strong** N = .[2]	**No or Unknown** N = .[2]	**Yes** N = .[2]	**No** N = .[2]	**Yes** N = .[2]	**No or Unknown** N = .[2]	**Yes** N = .[2]	**<5%** N = .[2]	**5-25%** N = .[2]	**25-50%** N = .[2]	**50-75%** N = .[2]	**>75%** N = .[2]
Non-vegetarian	.	.	.	.	.	.	.	.	.	.	.	.	.	.	.	.	.	.	.
Vegetarian (including egg)	.	.	.	.	.	.	.	.	.	.	.	.	.	.	.	.	.	.	.
Unknown	.	.	.	.	.	.	.	.	.	.	.	.	.	.	.	.	.	.	.
**ECOG**	.	.	.	.	.	.	.	.	.	.	.	.	.	.	.	.	.	.	.
0	.	.	.	.	.	.	.	.	.	.	.	.	.	.	.	.	.	.	.
1	.	.	.	.	.	.	.	.	.	.	.	.	.	.	.	.	.	.	.
2	.	.	.	.	.	.	.	.	.	.	.	.	.	.	.	.	.	.	.
**Time from surgery to randomisation (months)**	.	.	.	.	.	.	.	.	.	.	.	.	.	.	.	.	.	.	.
Mean (SD)	.	.	.	.	.	.	.	.	.	.	.	.	.	.	.	.	.	.	.
Median (Q1 - Q3)	.	.	.	.	.	.	.	.	.	.	.	.	.	.	.	.	.	.	.
Min - Max	.	.	.	.	.	.	.	.	.	.	.	.	.	.	.	.	.	.	.
**Primary tumour location**	.	.	.	.	.	.	.	.	.	.	.	.	.	.	.	.	.	.	.
Ascending colon	.	.	.	.	.	.	.	.	.	.	.	.	.	.	.	.	.	.	.
Caecum	.	.	.	.	.	.	.	.	.	.	.	.	.	.	.	.	.	.	.
Descending colon	.	.	.	.	.	.	.	.	.	.	.	.	.	.	.	.	.	.	.
Hepatic flexure	.	.	.	.	.	.	.	.	.	.	.	.	.	.	.	.	.	.	.
Rectum	.	.	.	.	.	.	.	.	.	.	.	.	.	.	.	.	.	.	.
Sigmoid colon/ rectosigmoid colon	.	.	.	.	.	.	.	.	.	.	.	.	.	.	.	.	.	.	.
Splenic flexure	.	.	.	.	.	.	.	.	.	.	.	.	.	.	.	.	.	.	.

	PIK3CA mutation[1]		Cox-2 Expression[1]		Cox-2 Expression (Ordinal scale)[1]				PIK3CA/PTEN mutation[1]		PIK3CA mutation (Exon 9, 20)[1]		PTEN mutation[1]		HLA class I expression[1]				
	**No or Unknown** N = .[2]	**Yes** N = .[2]	**Negative** N = .[2]	**Positive** N = .[2]	**Absent** N = .[2]	**Weak** N = .[2]	**Moderate** N = .[2]	**Strong** N = .[2]	**No or Unknown** N = .[2]	**Yes** N = .[2]	**No** N = .[2]	**Yes** N = .[2]	**No or Unknown** N = .[2]	**Yes** N = .[2]	**<5%** N = .[2]	**5-25%** N = .[2]	**25-50%** N = .[2]	**50-75%** N = .[2]	**>75%** N = .[2]
Transverse colon	.	.	.	.	.	.	.	.	.	.	.	.	.	.	.	.	.	.	.
Unknown	.	.	.	.	.	.	.	.	.	.	.	.	.	.	.	.	.	.	.
**T stage**	.	.	.	.	.	.	.	.	.	.	.	.	.	.	.	.	.	.	.
T1	.	.	.	.	.	.	.	.	.	.	.	.	.	.	.	.	.	.	.
T2	.	.	.	.	.	.	.	.	.	.	.	.	.	.	.	.	.	.	.
T3	.	.	.	.	.	.	.	.	.	.	.	.	.	.	.	.	.	.	.
T4	.	.	.	.	.	.	.	.	.	.	.	.	.	.	.	.	.	.	.
Unknown	.	.	.	.	.	.	.	.	.	.	.	.	.	.	.	.	.	.	.
**N stage**	.	.	.	.	.	.	.	.	.	.	.	.	.	.	.	.	.	.	.
0	.	.	.	.	.	.	.	.	.	.	.	.	.	.	.	.	.	.	.
1	.	.	.	.	.	.	.	.	.	.	.	.	.	.	.	.	.	.	.
2	.	.	.	.	.	.	.	.	.	.	.	.	.	.	.	.	.	.	.
Unknown	.	.	.	.	.	.	.	.	.	.	.	.	.	.	.	.	.	.	.
**Tumour type**	.	.	.	.	.	.	.	.	.	.	.	.	.	.	.	.	.	.	.
Dukes c colon cancer	.	.	.	.	.	.	.	.	.	.	.	.	.	.	.	.	.	.	.
High risk dukes b colon cancer	.	.	.	.	.	.	.	.	.	.	.	.	.	.	.	.	.	.	.
Rectal cancer (dukes b or dukes c)	.	.	.	.	.	.	.	.	.	.	.	.	.	.	.	.	.	.	.
**Chemotherapy regimen**	.	.	.	.	.	.	.	.	.	.	.	.	.	.	.	.	.	.	.
5-fu bolus	.	.	.	.	.	.	.	.	.	.	.	.	.	.	.	.	.	.	.
Capecitabine	.	.	.	.	.	.	.	.	.	.	.	.	.	.	.	.	.	.	.

	PIK3CA mutation[1]		Cox-2 Expression[1]		Cox-2 Expression (Ordinal scale)[1]				PIK3CA/PTEN mutation[1]		PIK3CA mutation (Exon 9, 20)[1]		PTEN mutation[1]		HLA class I expression[1]				
	**No or Unknown** N = .[2]	**Yes** N = .[2]	**Negative** N = .[2]	**Positive** N = .[2]	**Absent** N = .[2]	**Weak** N = .[2]	**Moderate** N = .[2]	**Strong** N = .[2]	**No or Unknown** N = .[2]	**Yes** N = .[2]	**No** N = .[2]	**Yes** N = .[2]	**No or Unknown** N = .[2]	**Yes** N = .[2]	**<5%** N = .[2]	**5-25%** N = .[2]	**25-50%** N = .[2]	**50-75%** N = .[2]	**>75%** N = .[2]
Capecitabine - oxaliplatin	.	.	.	.	.	.	.	.	.	.	.	.	.	.	.	.	.	.	.
Folfox	.	.	.	.	.	.	.	.	.	.	.	.	.	.	.	.	.	.	.
-usional 5-fu	.	.	.	.	.	.	.	.	.	.	.	.	.	.	.	.	.	.	.
Others	.	.	.	.	.	.	.	.	.	.	.	.	.	.	.	.	.	.	.
Unknown	.	.	.	.	.	.	.	.	.	.	.	.	.	.	.	.	.	.	.
**Chemotherapy cycle duration (weeks)**	.	.	.	.	.	.	.	.	.	.	.	.	.	.	.	.	.	.	.
Mean (SD)	.	.	.	.	.	.	.	.	.	.	.	.	.	.	.	.	.	.	.
Median (Q1 - Q3)	.	.	.	.	.	.	.	.	.	.	.	.	.	.	.	.	.	.	.
Min - Max	.	.	.	.	.	.	.	.	.	.	.	.	.	.	.	.	.	.	.
Unknown	.	.	.	.	.	.	.	.	.	.	.	.	.	.	.	.	.	.	.
**No. of cycles completed**	.	.	.	.	.	.	.	.	.	.	.	.	.	.	.	.	.	.	.
Mean (SD)	.	.	.	.	.	.	.	.	.	.	.	.	.	.	.	.	.	.	.
Median (Q1 - Q3)	.	.	.	.	.	.	.	.	.	.	.	.	.	.	.	.	.	.	.
Min - Max	.	.	.	.	.	.	.	.	.	.	.	.	.	.	.	.	.	.	.
Unknown	.	.	.	.	.	.	.	.	.	.	.	.	.	.	.	.	.	.	.
**Time from surgery to first chemotherapy (days)**	.	.	.	.	.	.	.	.	.	.	.	.	.	.	.	.	.	.	.
Mean (SD)	.	.	.	.	.	.	.	.	.	.	.	.	.	.	.	.	.	.	.
Median (Q1 - Q3)	.	.	.	.	.	.	.	.	.	.	.	.	.	.	.	.	.	.	.
Min - Max	.	.	.	.	.	.	.	.	.	.	.	.	.	.	.	.	.	.	.

	PIK3CA mutation[1]		Cox-2 Expression[1]		Cox-2 Expression (Ordinal scale)[1]				PIK3CA/PTEN mutation[1]		PIK3CA mutation (Exon 9, 20)[1]		PTEN mutation[1]		HLA class I expression[1]				
	**No or Unknown** N = .[2]	**Yes** N = .[2]	**Negative** N = .[2]	**Positive** N = .[2]	**Absent** N = .[2]	**Weak** N = .[2]	**Moderate** N = .[2]	**Strong** N = .[2]	**No or Unknown** N = .[2]	**Yes** N = .[2]	**No** N = .[2]	**Yes** N = .[2]	**No or Unknown** N = .[2]	**Yes** N = .[2]	**<5%** N = .[2]	**5-25%** N = .[2]	**25-50%** N = .[2]	**50-75%** N = .[2]	**>75%** N = .[2]
Unknown	.	.	.	.	.	.	.	.	.	.	.	.	.	.	.	.	.	.	.
**Radiotherapy regimen (for rectal cancer only)**	.	.	.	.	.	.	.	.	.	.	.	.	.	.	.	.	.	.	.
Adjuvant chemo rt	.	.	.	.	.	.	.	.	.	.	.	.	.	.	.	.	.	.	.
Neoadjuvant chemo rt	.	.	.	.	.	.	.	.	.	.	.	.	.	.	.	.	.	.	.
Neoadjuvant rt	.	.	.	.	.	.	.	.	.	.	.	.	.	.	.	.	.	.	.
No rt	.	.	.	.	.	.	.	.	.	.	.	.	.	.	.	.	.	.	.
Unknown	.	.	.	.	.	.	.	.	.	.	.	.	.	.	.	.	.	.	.
**Time from radiotherapy to randomisation (months)(for rectal cancer only)**	.	.	.	.	.	.	.	.	.	.	.	.	.	.	.	.	.	.	.
Mean (SD)	.	.	.	.	.	.	.	.	.	.	.	.	.	.	.	.	.	.	.
Median (Q1 - Q3)	.	.	.	.	.	.	.	.	.	.	.	.	.	.	.	.	.	.	.
Min - Max	.	.	.	.	.	.	.	.	.	.	.	.	.	.	.	.	.	.	.
Unknown	.	.	.	.	.	.	.	.	.	.	.	.	.	.	.	.	.	.	.
**Dose of radiotherapy (for rectal cancer only)**	.	.	.	.	.	.	.	.	.	.	.	.	.	.	.	.	.	.	.
Mean (SD)	.	.	.	.	.	.	.	.	.	.	.	.	.	.	.	.	.	.	.
Median (Q1 - Q3)	.	.	.	.	.	.	.	.	.	.	.	.	.	.	.	.	.	.	.
Min - Max	.	.	.	.	.	.	.	.	.	.	.	.	.	.	.	.	.	.	.

	PIK3CA mutation[1]		Cox-2 Expression[1]		Cox-2 Expression (Ordinal scale)[1]				PIK3CA/PTEN mutation[1]		PIK3CA mutation (Exon 9, 20)[1]		PTEN mutation[1]		HLA class I expression[1]				
	**No or Unknown** N = .[2]	**Yes** N = .[2]	**Negative** N = .[2]	**Positive** N = .[2]	**Absent** N = .[2]	**Weak** N = .[2]	**Moderate** N = .[2]	**Strong** N = .[2]	**No or Unknown** N = .[2]	**Yes** N = .[2]	**No** N = .[2]	**Yes** N = .[2]	**No or Unknown** N = .[2]	**Yes** N = .[2]	**<5%** N = .[2]	**5-25%** N = .[2]	**25-50%** N = .[2]	**50-75%** N = .[2]	**>75%** N = .[2]
Unknown	.	.	.	.	.	.	.	.	.	.	.	.	.	.	.	.	.	.	.
**No. of radiotherapy fractions (for rectal cancer only)**	.	.	.	.	.	.	.	.	.	.	.	.	.	.	.	.	.	.	.
Mean (SD)	.	.	.	.	.	.	.	.	.	.	.	.	.	.	.	.	.	.	.
Median (Q1 - Q3)	.	.	.	.	.	.	.	.	.	.	.	.	.	.	.	.	.	.	.
Min - Max	.	.	.	.	.	.	.	.	.	.	.	.	.	.	.	.	.	.	.
Unknown	.	.	.	.	.	.	.	.	.	.	.	.	.	.	.	.	.	.	.
**Tumour type**	.	.	.	.	.	.	.	.	.	.	.	.	.	.	.	.	.	.	.
Dukes c colon cancer	.	.	.	.	.	.	.	.	.	.	.	.	.	.	.	.	.	.	.
High risk dukes b colon cancer	.	.	.	.	.	.	.	.	.	.	.	.	.	.	.	.	.	.	.
Rectal cancer (dukes b or dukes c)	.	.	.	.	.	.	.	.	.	.	.	.	.	.	.	.	.	.	.

[1]Modified intention-to-treat (mITT) with available tissue population consists of all subjects who signed the informed consent form for the study enrollment, commenced study treatment after the randomisation and have available tissue sample for assessments.

[2]n (%)

BASELINE CHARACTERISTICS ROW PERCENTAGES TABLES

PIK3CA MUTATIONS AND COX-2 EXPRESSION

	PIK3CA mutation[1]		Cox-2 Expression[1]	
	**No or Unknown** N = .	**Yes** N = .	**Negative** N = .	**Positive** N = .
**Age**	.	.	.	.
Median (Q1 - Q3)	.	.	.	.
**Gender**	.	.	.	.
Female	.	.	.	.
Male	.	.	.	.
**Ethnicity**	.	.	.	.
Asian	.	.	.	.
White	.	.	.	.
Others	.	.	.	.
**Region**	.	.	.	.
Australia & New Zealand	.	.	.	.
Singapore, Malaysia, Taiwan	.	.	.	.
**ECOG**	.	.	.	.
0	.	.	.	.
1	.	.	.	.
2	.	.	.	.
**Time from surgery to randomisation (months)**	.	.	.	.
Median (Q1 - Q3)	.	.	.	.
**Disease site**	.	.	.	.
Right side of colon	.	.	.	.
Left side of colon	.	.	.	.
Rectum	.	.	.	.
**T stage**	.	.	.	.
T1	.	.	.	.
T2	.	.	.	.
T3	.	.	.	.
T4	.	.	.	.
Unknown	.	.	.	.
**N stage**	.	.	.	.
0	.	.	.	.
1	.	.	.	.
2	.	.	.	.
**Chemotherapy type**	.	.	.	.
Exposed to oxaliplatin	.	.	.	.
Not exposed to oxaliplatin	.	.	.	.
**Radiotherapy given**	.	.	.	.
Yes	.	.	.	.
No	.	.	.	.
**Chemotherapy duration**	.	.	.	.

	PIK3CA mutation[1]		Cox-2 Expression[1]	
	**No or Unknown** N = .	**Yes** N = .	**Negative** N = .	**Positive** N = .
<=6 months	.	.	.	.
>6 months	.	.	.	.

[1]Modified intention-to-treat (mITT) with available tissue population consists of all subjects who signed the informed consent form for the study enrollment, commenced study treatment after the randomisation and have available tissue sample for assessments.

PIK3CA MUTATIONS EXON 9, 20 AND PTEN

Same table structure as above

CLINICAL EVENTS - FOR ALL M-ITT PATIENTS (N = .)

CLINICAL EVENTS BY COUNTRY

	m-ITT with Available Tissue[1]					
	**Overall** N = .[2]	**Australia** N = .[2]	**New Zealand** N = .[2]	**Singapore** N = .[2]	**Malaysia** N = .[2]	**Taiwan** N = .[2]
**Death**	.	.	.	.	.	.
**Recurrence**	.	.	.	.	.	.
**Recurrence or death**	.	.	.	.	.	.

[1]Modified intention-to-treat (mITT) with available tissue population consists of all subjects who signed the informed consent form for the study enrollment, commenced study treatment after the randomisation and have available tissue sample for assessments.

[2]n (%)

CLINICAL EVENTS BY TREATMENT GROUP (ANZ)

	m-ITT with Available Tissue[1]		
	**Overall** N = .[2]	**Placebo** N = .[2]	**Aspirin** N = .[2]
**Death**	.	.	.
**Recurrence**	.	.	.
**Recurrence or death**	.	.	.

[1]Modified intention-to-treat (mITT) with available tissue population consists of all subjects who signed the informed consent form for the study enrollment, commenced study treatment after the randomisation and have available tissue sample for assessments.

[2]n (%)

CLINICAL EVENTS BY TREATMENT GROUP (SINGAPORE)

Same table structure as above

CLINICAL EVENTS BY TREATMENT GROUP (TAIWAN)

Same table structure as above

CLINICAL EVENTS BY TREATMENT GROUP (MALAYSIA)

Same table structure as above

CLINICAL EVENTS BY SUBGROUP

PIK3CA MUTATION STATUS - FOR PATIENTS WITH AVAILABLE TISSUE RESULTS (N = .)

PIK3CA mutation status is identified by any of the following:

- PIK3CA mutation status (PIK3CA) (by NGS) (N = .)
- PIK3CA mutation status (PIK3CA) (by Sanger) (N = .)

	PIK3CA mutation: No or Unknown[1]			PIK3CA mutation: Yes[1]		
	Overall N = .[2]	Placebo N = .[2]	Aspirin N = .[2]	Overall N = .[2]	Placebo N = .[2]	Aspirin N = .[2]
**Death**	.	.	.	.	.	.
**Recurrence**	.	.	.	.	.	.
**Recurrence or death**	.	.	.	.	.	.

[1]m-ITT with available tissue patients

[2]n (%) [95% Confidence Interval]; CI: confidence interval for proportion based on Wilson score method. Wilson, E.B. (1927). 'Probable inference, the law of succession, and statistical inference'. Journal of the American Statistical Association. 22 (158): 209–212.

COX2 EXPRESSION STATUS - FOR PATIENTS WITH AVAILABLE TISSUE RESULTS (N = .)

Same table structure as above

COX2 EXPRESSION STATUS (CATEGORICAL) - FOR PATIENTS WITH AVAILABLE TISSUE RESULTS (N = .)

PIK3CA AND PTEN MUTATION STATUS - FOR PATIENTS WITH AVAILABLE TISSUE RESULTS (N = .)

PIK3CA/PTEN mutation status is identified by any of the following:

- PIK3CA mutation status (PIK3CA) (by NGS or Sanger) (N = .)
- PTEN mutation status (PTEN) (by NGS or Sanger)(N = .)

Same table structure as above

PIK3CA (EXON 9, 20) MUTATION STATUS - FOR PATIENTS WITH AVAILABLE TISSUE RESULTS (N = .)

PIK3CA (EXON 9, 20) mutation status is identified by any of the following:

- PIK3CA (EXON 9, 20) mutation status (PIK3CA) (by NGS or Sanger) (N = .)

Same table structure as above

TREATMENT EFFECT

DISEASE FREE SURVIVAL (DFS) - FOR ALL M-ITT PATIENTS (N = .)

	m-ITT with Available Tissue[1]				
	N	Event N	HR	95% CI	p-value
**Treatment allocation**	.	.	.	.	.
Placebo	.	.	.	.	.
Aspirin	.	.	.	.	.

[1]Modified intention-to-treat (mITT) with available tissue population consists of all subjects who signed the informed consent form for the study enrollment, commenced study treatment after the randomisation and have available tissue sample for assessments.

Abbreviations: CI = Confidence Interval, HR = Hazard Ratio

OVERALL SURVIVAL (OS) - FOR ALL M-ITT PATIENTS (N = .)

Same table structure as above

DISEASE FREE SURVIVAL (DFS) - FOR PATIENTS WITH AVAILABLE PIK3CA TISSUE RESULTS (N = .)

Same table structure as above

OVERALL SURVIVAL (OS) - FOR PATIENTS WITH AVAILABLE PIK3CA TISSUE RESULTS (N = .)

Same table structure as above

TREATMENT EFFECT BY BIOMARKER STATUS SUBGROUPS

DISEASE FREE SURVIVAL (DFS)

	m-ITT with Available Tissue[1]				
	N	Event N	HR	95% CI	p-value
**PIK3CA mutation: No or Unknown**					
**Treatment allocation**	.	.	.	.	.
Placebo	.	.	.	.	.
Aspirin	.	.	.	.	.
**PIK3CA mutation: Yes**					
**Treatment allocation**	.	.	.	.	.
Placebo	.	.	.	.	.
Aspirin	.	.	.	.	.
**Cox-2 Expression: Negative**					
**Treatment allocation**	.	.	.	.	.
Placebo	.	.	.	.	.
Aspirin	.	.	.	.	.
**Cox-2 Expression: Positive**					
**Treatment allocation**	.	.	.	.	.
Placebo	.	.	.	.	.
Aspirin	.	.	.	.	.
**Cox-2 Expression (Ordinal scale): Absent**					
**Treatment allocation**	.	.	.	.	.
Placebo	.	.	.	.	.
Aspirin	.	.	.	.	.
**Cox-2 Expression (Ordinal scale): Weak**					
**Treatment allocation**	.	.	.	.	.
Placebo	.	.	.	.	.
Aspirin	.	.	.	.	.
**Cox-2 Expression (Ordinal scale): Moderate**					
**Treatment allocation**	.	.	.	.	.
Placebo	.	.	.	.	.
Aspirin	.	.	.	.	.
**Cox-2 Expression (Ordinal scale): Strong**					
**Treatment allocation**	.	.	.	.	.
Placebo	.	.	.	.	.
Aspirin	.	.	.	.	.
**PIK3CA/PTEN mutation: No or Unknown**					
**Treatment allocation**	.	.	.	.	.
Placebo	.	.	.	.	.
Aspirin	.	.	.	.	.

	m-ITT with Available Tissue[1]				
	N	Event N	HR	95% CI	p-value
**PIK3CA/PTEN mutation: Yes**					
**Treatment allocation**	.	.	.	.	.
Placebo	.	.	.	.	.
Aspirin	.	.	.	.	.
**PIK3CA mutation (Exon 9, 20): No**					
**Treatment allocation**	.	.	.	.	.
Placebo	.	.	.	.	.
Aspirin	.	.	.	.	.
**PIK3CA mutation (Exon 9, 20): Yes**					
**Treatment allocation**	.	.	.	.	.
Placebo	.	.	.	.	.
Aspirin	.	.	.	.	.
**PIK3CA mutation (Non-Exon 9, 20): No or Unknown**					
**Treatment allocation**	.	.	.	.	.
Placebo	.	.	.	.	.
Aspirin	.	.	.	.	.
**PIK3CA mutation (Non-Exon 9, 20): Yes**					
**Treatment allocation**	.	.	.	.	.
Placebo	.	.	.	.	.
Aspirin	.	.	.	.	.
**HLA class I expression: <5%**					
**Treatment allocation**	.	.	.	.	.
Placebo	.	.	.	.	.
Aspirin	.	.	.	.	.
**HLA class I expression: 5-25%**					
**Treatment allocation**	.	.	.	.	.
Placebo	.	.	.	.	.
Aspirin	.	.	.	.	.
**HLA class I expression: 25-50%**					
**Treatment allocation**	.	.	.	.	.
Placebo	.	.	.	.	.
Aspirin	.	.	.	.	.
**HLA class I expression: 50-75%**					
**Treatment allocation**	.	.	.	.	.
Placebo	.	.	.	.	.
Aspirin	.	.	.	.	.

	m-ITT with Available Tissue[1]				
	N	Event N	HR	95% CI	p-value
**HLA class I expression: >75%**					
**Treatment allocation**	.	.	.	.	.
Placebo	.	.	.	.	.
Aspirin	.	.	.	.	.
**PTEN mutation: No or Unknown**					
**Treatment allocation**	.	.	.	.	.
Placebo	.	.	.	.	.
Aspirin	.	.	.	.	.
**PTEN mutation: Yes**					
**Treatment allocation**	.	.	.	.	.
Placebo	.	.	.	.	.
Aspirin	.	.	.	.	.
**PIK3CA/PTEN mutation (Non-Exon 9, 20): No or Unknown**					
**Treatment allocation**	.	.	.	.	.
Placebo	.	.	.	.	.
Aspirin	.	.	.	.	.
**PIK3CA/PTEN mutation (Non-Exon 9, 20): Yes**					
**Treatment allocation**	.	.	.	.	.
Placebo	.	.	.	.	.
Aspirin	.	.	.	.	.

[1]Modified intention-to-treat (mITT) with available tissue population consists of all subjects who signed the informed consent form for the study enrollment, commenced study treatment after the randomisation and have available tissue sample for assessments.

Abbreviations: CI = Confidence Interval, HR = Hazard Ratio

OVERALL SURVIVAL (OS)

Same table structure as above

KAPLAN-MEIER SURVIVAL ESTIMATES

DISEASE FREE SURVIVAL

DISEASE FREE SURVIVAL BY TREATMENT ARM

	m-ITT with Available Tissue		
	All	Placebo	Aspirin
N[1]	.[1]	.[1]	.[1]
Events[2]	.[2]	.[2]	.[2]
Median Follow-up months (95% CIs)	.	.	.
Median DFS months (95% CIs)	.	.	.
DFS proportion at: 36 months (95% CIs)	.	.	.
DFS proportion at: 60 months (95% CIs)	.	.	.

[1]m-ITT with available tissue patients

[2]Total events during follow-up period.

DISEASE FREE SURVIVAL BY PIK3CA MUTATION STATUS

	PIK3CA mutation: No or Unknown			PIK3CA mutation: Yes		
	All	Placebo	Aspirin	All	Placebo	Aspirin
N[1]	.[1]	.[1]	.[1]	.[1]	.[1]	.[1]
Events[2]	.[2]	.[2]	.[2]	.[2]	.[2]	.[2]
Median Follow-up months (95% CIs)	.	.	.	.	.	.
Median DFS months (95% CIs)	.	.	.	.	.	.
DFS proportion at: 36 months (95% CIs)	.	.	.	.	.	.
DFS proportion at: 60 months (95% CIs)	.	.	.	.	.	.

[1]m-ITT with available tissue patients

[2]Total events during follow-up period.

DISEASE FREE SURVIVAL BY COX2 EXPRESSION STATUS

Same table structure as above

Same table structure as above

DISEASE FREE SURVIVAL BY PIK3CA AND PTEN MUTATION STATUS

Same table structure as above

DISEASE FREE SURVIVAL BY PIK3CA (EXON 9, 20) MUTATION STATUS

Same table structure as above

DISEASE FREE SURVIVAL BY HLA CLASS I EXPRESSION STATUS

Same table structure as above

OVERALL SURVIVAL

OVERALL SURVIVAL BY TREATMENT ARM

	m-ITT with Available Tissue		
	All	Placebo	Aspirin
N[1]	.[1]	.[1]	.[1]
Events[2]	.[2]	.[2]	.[2]
Median Follow-up months (95% CIs)	.	.	.
Median OS months (95% CIs)	.	.	.
OS proportion at: 36 months (95% CIs)	.	.	.
OS proportion at: 60 months (95% CIs)	.	.	.

[1]m-ITT with available tissue patients

[2]Total events during follow-up period.

OVERALL SURVIVAL BY PIK3CA MUTATION STATUS

	PIK3CA mutation: No or Unknown			PIK3CA mutation: Yes		
	All	Placebo	Aspirin	All	Placebo	Aspirin
N[1]	.[1]	.[1]	.[1]	.[1]	.[1]	.[1]
Events[2]	.[2]	.[2]	.[2]	.[2]	.[2]	.[2]
Median Follow-up months (95% CIs)	.	.	.	.	.	.
Median OS months (95% CIs)	.	.	.	.	.	.
OS proportion at: 36 months (95% CIs)	.	.	.	.	.	.
OS proportion at: 60 months (95% CIs)	.	.	.	.	.	.

[1]m-ITT with available tissue patients

[2]Total events during follow-up period.

OVERALL SURVIVAL BY COX2 EXPRESSION STATUS

Same table structure as above

OVERALL SURVIVAL BY COX2 EXPRESSION LEVELS

Same table structure as above

OVERALL SURVIVAL BY PIK3CA AND PTEN MUTATION STATUS

Same table structure as above

OVERALL SURVIVAL BY PIK3CA (EXON 9, 20) MUTATION STATUS

Same table structure as above

OVERALL SURVIVAL BY HLA CLASS I EXPRESSION STATUS

Same table structure as above

META ANALYSIS OF TREATMENT EFFECT BY BIOMARKER STATUS SUBGROUPS

This section contains meta analysis of treatment effect in biomarker status subgroups, using data from ASCOLT TR, ALASSCA and SAKK 41/13.

PIK3CA MUTATIONS EXON 9, 20

Insert meta analysis forest plot for PIK3CA mutations exon 9, 20 here.

OTHER PI3K (OTHER PIK3CA, PIK3R1, PTEN)

Insert meta analysis forest plot for other PI3K (other PIK3CA, PIK3R1, PTEN) here.

ANY PI3K ALTERATIONS (PIK3CA, PIK3R1, PTEN)

Insert meta analysis forest plot for any PI3K alterations (PIK3CA, PIK3R1, PTEN) here.

Study Statistician: Isabel Li

Study Statistician Signature: ______________________

Trial Statistician: Val Gebski

Study Statistician Signature: ______________________
